# Restriction Landmark Genomic Scanning (RLGS) spot identification by second generation virtual RLGS in multiple genomes with multiple enzyme combinations

**DOI:** 10.1186/1471-2164-8-446

**Published:** 2007-11-30

**Authors:** Dominic J Smiraglia, Ramakrishnan Kazhiyur-Mannar, Christopher C Oakes, Yue-Zhong Wu, Ping Liang, Tahmina Ansari, Jian Su, Laura J Rush, Laura T Smith, Li Yu, Chunhui Liu, Zunyan Dai, Shih-Shih Chen, Shu-Huei Wang, Joseph Costello, Ilya Ioshikhes, David W Dawson, Jason S Hong, Michael A Teitell, Angela Szafranek, Marta Camoriano, Fei Song, Rosemary Elliott, William Held, Jacquetta M Trasler, Christoph Plass, Rephael Wenger

**Affiliations:** 1 Department of Cancer Genetics, and Comprehensive Cancer Center, Roswell Park Cancer Institute, Buffalo, NY, USA; 2 Department of Computer Science and Engineering, and Comprehensive Cancer Center, The Ohio State University, Columbus, Ohio, USA; 3 Department of Pharmacology and Therapeutics, McGill University, Montreal, Quebec, Canada; 4 Department of Molecular Virology, Immunology, and Medical Genetics, and Comprehensive Cancer Center, The Ohio State University, Columbus, Ohio, USA; 5 Department of Veterinary Biosciences, and Comprehensive Cancer Center, The Ohio State University, Columbus, Ohio, USA; 6 Department of Pharmacology, Davis Heart and Lung Research Institute, and Comprehensive Cancer Center, The Ohio State University, Columbus, Ohio, USA; 7 Department of Neurological Surgery and The Brain Tumor Research Center, and Comprehensive Cancer Center, University of California, San Francisco, San Francisco, California, USA; 8 Department of Biomedical Informatics, Davis Heart and Lung Research Institute, and Comprehensive Cancer Center, The Ohio State University, Columbus, Ohio, USA; 9 Department of Pathology and Laboratory Medicine, Jonsson Comprehensive Cancer Center, University of California, Los Angeles, Los Angeles, California, USA; 10 Department of Molecular Biology Institute, Jonsson Comprehensive Cancer Center, University of California, Los Angeles, Los Angeles, California, USA; 11 Department of Cell and Molecular Biology, and Comprehensive Cancer Center, Roswell Park Cancer Institute, Buffalo, NY, USA; 12 Department of Pediatrics, McGill University, Montreal, Quebec, Canada; 13 Department of Human Genetics, McGill University, Montreal, Quebec, Canada

## Abstract

**Background:**

Restriction landmark genomic scanning (RLGS) is one of the most successfully applied methods for the identification of aberrant CpG island hypermethylation in cancer, as well as the identification of tissue specific methylation of CpG islands. However, a limitation to the utility of this method has been the ability to assign specific genomic sequences to RLGS spots, a process commonly referred to as "RLGS spot cloning."

**Results:**

We report the development of a virtual RLGS method (vRLGS) that allows for RLGS spot identification in any sequenced genome and with any enzyme combination. We report significant improvements in predicting DNA fragment migration patterns by incorporating sequence information into the migration models, and demonstrate a median Euclidian distance between actual and predicted spot migration of 0.18 centimeters for the most complex human RLGS pattern. We report the confirmed identification of 795 human and 530 mouse RLGS spots for the most commonly used enzyme combinations. We also developed a method to filter the virtual spots to reduce the number of extra spots seen on a virtual profile for both the mouse and human genomes. We demonstrate use of this filter to simplify spot cloning and to assist in the identification of spots exhibiting tissue-specific methylation.

**Conclusion:**

The new vRLGS system reported here is highly robust for the identification of novel RLGS spots. The migration models developed are not specific to the genome being studied or the enzyme combination being used, making this tool broadly applicable. The identification of hundreds of mouse and human RLGS spot loci confirms the strong bias of RLGS studies to focus on CpG islands and provides a valuable resource to rapidly study their methylation.

## Background

Restriction landmark genomic scanning (RLGS) has been used to study aberrant CpG island methylation in cancer for more than ten years. This approach remains one of the most reliable ways to characterize CpG island hypermethylation in cancer and has been used both to characterize differences in aberrant methylation phenotypes and also to identify tumor suppressor genes. Not only have known tumor suppressor genes like Cdkn2a (p16), Itga4 (α 4-integrin) [1], and Igfbp7 [2] been identified as targets of aberrant methylation in cancer by RLGS, but also novel tumor suppressor genes such as TCF21 [3], SLC5A8 [4], ID4 [5], BMP3B [6], and SOCS1 [7] have been identified by RLGS.

RLGS is a two-dimensional gel electrophoresis method [8] that allows detection of DNA methylation if a methylation sensitive landmark enzyme such as NotI is used. Up to 2,000 end-labeled landmark sites are displayed in a single RLGS profile. The labeling of the sites is based on incorporation of radionucleotides into the NotI half-site by DNA polymerase. Methylated sites are not digested and are therefore not labeled, thus they do not contribute to the two-dimensional pattern of RLGS fragments. Spots present in a normal profile, but absent from a tumor profile are indicative of methylation of the landmark site. Furthermore, the profiles are quantitative such that spot intensity directly correlates with the degree of methylation of a locus, with partial methylation representing the cellular heterogeneity of the cancer both among the malignant cells and the associated non-malignant cells. RLGS profiles are highly reproducible allowing for comparison of different tissues (e.g. normal versus tumor tissue) or of different individuals. Although this approach does not cover every CpG island in the genome, as microarray-based approaches may achieve [9,10], RLGS provides highly reliable data due to the molecular simplicity of the assay. Unlike microarray-based techniques that depend upon molecularly complex manipulations such as whole genome PCR, hybridization, and immunoprecipitation of methylated DNA, RLGS simply relies upon restriction digestion of DNA and gel electrophoresis.

Crucial to the successful implementation of RLGS for a CpG island methylation profiling study is the ability to identify the sequence of the targets of aberrant methylation, which has been a bottleneck in the flow of these types of studies. Significant improvements were made in RLGS spot cloning ability with the advent of arrayed boundary libraries, where restriction fragments from the arrayed library were mapped to the pattern of RLGS spots [11-13]. Although highly successful, this method is limited by the tremendous investment in effort required for each genome of interest and each enzyme combination of interest.

With the completion of the human and mouse genome sequences, a bioinformatics approach to RLGS fragment identification became possible where each restriction fragment's migration in both dimensions could be predicted to create a virtual RLGS profile. Such an approach could be applied to any sequenced genome, using any enzyme combination, and is unaffected by any potential tissue specific methylation. Furthermore, such an approach should be far less labor intensive to develop and use than the arrayed boundary library spot cloning method. The relative ease of setup and use, plus the incredible flexibility in the virtual approach, makes such a development highly attractive, allowing any researcher the ability to identify RLGS spots without the creation of specialized reagents.

This idea was partially realized in 2001 with a first generation version of a virtual RLGS gel that was called "Virtual Genome Scan" and applied to the human genome [14]. In this system, the restriction fragment migration prediction formula was based solely on fragment length. Based on known fragment lengths and spot positions in both dimensions of rDNA and EBV genome derived spots, a cubic polynomial was derived giving fragment length as a function of spot location. By comparing the known positions of 22 previously identified spots with the positions of their corresponding fragments on the virtual gel, it was reported that 75% (16/22) of fragments were in a 12 × 30 pixel window (0.5 cm × 1.5 cm) around the predicted location, with the remaining fragments in a 24 × 128 pixel window (1.0 cm × 6.4 cm) [14]. This approach was used to identify one previously unknown RLGS spot methylated in neuroblastoma and found to be in the 5' end of the ALX3 gene [15]. Taking a similar approach, but incorporating the known chromosome of origin of the RLGS spots [16] into the predictions, Zardo et al (2002) [17] determined 96 previously unknown spot identities, and Hong et al (2003) [18] determined approximately 100 additional new spot identities.

A second virtual RLGS system (viRLGS) was reported in 2003 and applied to the arabidopsis and mouse genomes [19]. The migration algorithms used were similarly based solely on the length of the DNA fragments. In the mouse virtual profile it was observed that there were 710 more spots on the virtual profile than are found on the same region of the actual profile. Given that in the mouse genome, unlike the human genome, many of the landmark sites (NotI sites) are within repetitive elements like LTRs, Repeat Masker was able to remove nearly 500 of these extra spots from the virtual profile [19]. This approach was used to identify tissue specifically methylated RLGS spots in mouse [20] and to identify CpG islands that become hypomethylated in mouse ES cells when DNA methyltransferases are knocked out [21].

With approximately 2500 spots on a 35 × 43 cm gel in the human NotI-EcoRV-HinfI enzyme combination most commonly used, the average distance between neighboring spots is 0.7 cm. To generate a virtual image that approximates the true image, such that the relative predicted positions of any two neighboring spots does not flip-flop, the window of error for spot prediction should be, at most, half this average distance or 0.35 cm. One confounding issue is that others have demonstrated a considerable correlation between DNA fragment curvature and gel mobility [22-25]. Thus, it is clear that, while a simple fragment migration model based upon fragment length alone is a good start, it must be improved upon. In addition, since in the human genome NotI sites rarely cut in repetitive elements, use of a repeat masker does not help to remove the extra spots in a human virtual RLGS profile.

We have developed a novel system of virtual RLGS (vRLGS) using first and second dimension migration algorithms that use the sequence characteristics of each fragment along with the fragment length to determine spot position. These algorithms were determined based on the identification of 795 RLGS spots in the human NotI-EcoRV-HinfI profile. In addition, we have defined a filter to remove extra spots from both the human and mouse vRLGS profiles based on GC content. We report greatly improved accuracy of pattern prediction even when the migration algorithms are applied to different enzyme combinations and different genomes from which they were derived. These improvements are combined with a novel graphic interface that allows for visualization of virtual spots that 1) look like real spots, and 2) allow the user to overlap the virtual and real profiles. These changes make the bioinformatics approach to identifying RLGS spots of interest a highly viable and effective alternative to more complex RLGS spot cloning methods.

## Results and discussion

### Spot location prediction

**Figure 1:**
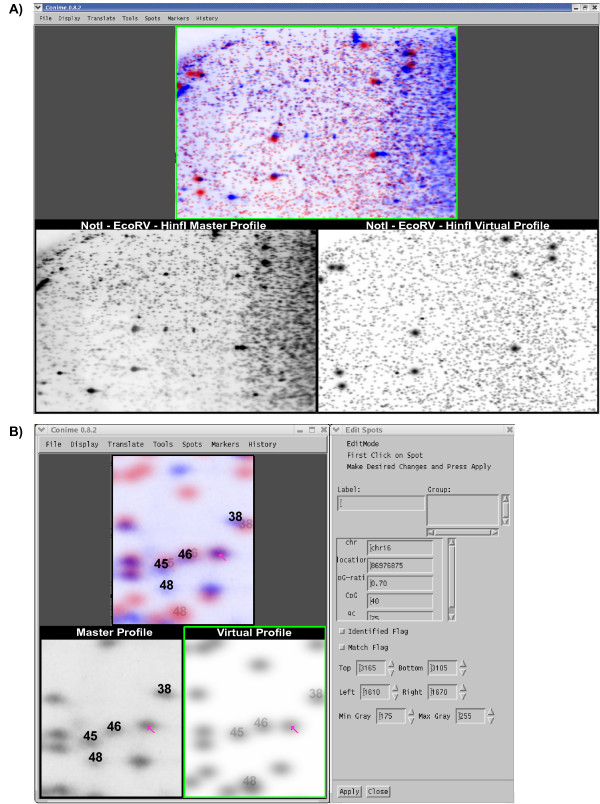
**vRLGS in the Conime interface**. Using the March 2006 freeze of the human genome we generated a vRLGS profile for the enzyme combination NotI-EcoRV-HinfI and loaded it into the Conime user interface along with the human master profile. **A) **Three veiwports in the Conime interface. The master profile is seen in the bottom left and the virtual profile is shown on the bottom right. The upper panel is a composite image of the two with the virtual profile contributing red and the master profile contributing blue. Areas of spot overlap between the two profiles appear as dark blue. **B) **Zoomed in view of the gels in A). Spots 38, 45, 46, and 48 are known sequences and are labeled on both the master profile and virtual profile with their overlap shown in the upper panel. The arrow in all three profiles indicates the spot of interest on the master profile, the top candidate sequence on the virtual profile, and their overlap. To the right is shown a window that can be selected to provide information about the virtual spot of interest (clicking on the spot with the arrow). This spot of interest is found at chr16:86976875.

vRLGS profiles for the human NotI-EcoRV-HinfI, AscI-EcoRV-HinfI, and the mouse NotI-EcoRV-HinfI genome and enzyme combinations were generated as described in the Materials and Methods. These virtual profiles were then compared to the laboratory gels that serve as our "Master Profiles" for each. Fig 1A contains a sample screen shot of Conime. The upper panel shows the overlap of the two gels and is colorized with the laboratory gel contributing blue and the virtual gel contributing red, while areas of overlap appear dark purple. The strongest spots on the laboratory gel arise from the high copy number rDNA sequences. Some of these spots line up quite well with the virtual spots, but others do not due to polymorphism in subsets of these highly repeated units. The virtual profile does not have polymorphic information for these subsets of the repeats, explaining why some of the darkest spots do not align. The image in Fig 1A is zoomed out to show the entire gel, while in Fig 1B the image is focused on a specific region of interest. This region of the gel is a portion of section 2D from the master PBL profile previously described [26]. Spots 38, 45, 46 and 48 have been identified and confirmed, and their positions on both the actual RLGS gel (left) and the virtual gel (right) are shown. The overlay in the top panel shows how closely the migration algorithms correctly predict the positions of the spots in both dimensions.

In order to identify a top candidate sequence for the spot adjacent to 46 with the arrow, the virtual profile is clickable. This clickable window presents information about the restriction fragment mapping to that position in the vRLGS profile, as shown in Fig 1B. Each vRLGS spot has a unique identifier, giving the chromosome followed by the base position on that chromosome. The unique identifier for each virtual spot is the position of either the NotI site or the HinfI site in the 2^nd ^dimension fragment, depending on the orientation of the sequence in the database with the 3' end of the restriction fragment used. Therefore, even though a single NotI site can potentially generate two spots, each will get a unique identifier with the spot generated by the fragment 5' to the NotI site named by the position of the NotI site, and the spot generated by the fragment 3' to the NotI site named by the position of the HinfI site. Additional information concerning the CpG characteristics of the region around the NotI site are also included and discussed in more detail below.

Using sets of confirmed cloned spots for each gel, we computed the distance between the predicted X and Y position and each spots' actual X and Y positions on the master profiles. We also computed the Euclidean distance between the spots' predicted and actual location on the gel. These distances represent the spot prediction error and give an objective measure of the accuracy of the DNA fragment migration algorithms in each dimension. Table 1 presents the results of the prediction formulas applied to the three gels. We give the median error, the mean error, and the root mean squared error for the X and Y dimensions as well as the Euclidean prediction errors, the 90'th percentile error, (the max error after dropping 10'th percentile of spots with the worst error,) and the root mean square error of the 90'th percentile of spots with the least error. The large improvement in the RMS of the 90^th ^percentile spots compared to the entire data set indicates that a relatively few number of outliers are strongly influencing the mean error. Importantly, the maximum difference between actual spot position and virtual spot position for the 75^th ^percentile of spots is 0.12 cm in the 1^st ^dimension and 0.24 cm in the 2^nd ^dimension. This represents a significant improvement over the maximum error for the 75^th ^percentile of spots of 0.5 cm and 1.5 cm previously reported for a virtual RLGS approach [14].

**Table 1:** Measure of migration prediction accuracy for all profiles

		**Human NotI-EcoRV-HinfI *(n = 795)***					**Human AscI-EcoRV-HinfI *(n = 133)***					**Mouse NotI-EcoRV-HinfI *(n = 530)***				
**Func**		**Median**	**Mean**	**RMS**^*c*^	**90%**^*d*^	**90%-RMS**	**Median**	**Mean**	**RMS**	**90%**	**90%-RMS**	**Median**	**Mean**	**RMS**	**90%**	**90%-RMS**

(1)^*a*^	X	0.17^*b*^	0.22	0.28	0.45	0.20	0.14	0.25	0.44	0.52	0.19	0.35	0.49	0.99	0.79	0.35
(2)	X	0.14	0.17	0.23	0.34	0.16	0.13	0.23	0.55	0.34	0.14	0.15	0.23	0.76	0.36	0.17
(3)	X	0.13	0.15	0.20	0.31	0.14	0.12	0.13	0.17	0.26	0.13	0.11	0.18	0.74	0.29	0.12
(4)	X	0.10	0.14	0.20	0.30	0.12	0.09	0.19	0.53	0.27	0.11	0.13	0.22	0.77	0.34	0.15
(5)	X	0.09	0.11	0.16	0.24	0.10	0.07	0.10	0.13	0.20	0.09	0.09	0.16	0.75	0.23	0.10
(6)	X	0.08	0.11	0.15	0.22	0.10	0.06	0.09	0.12	0.18	0.08	0.09	0.16	0.75	0.22	0.10
(7)	X	0.06	0.09	0.14	0.18	0.08	0.07	0.08	0.11	0.18	0.08	0.05	0.12	0.74	0.15	0.06
																

(8)	Y	0.26	0.48	0.85	1.08	0.36	0.26	0.37	0.53	0.82	0.32	0.28	0.49	0.94	1.04	0.37
(9)	Y	0.25	0.40	1.77	0.71	0.30	0.23	0.37	0.81	0.61	0.26	0.29	0.47	1.11	0.80	0.34
(10)	Y	0.21	0.45	4.44	0.55	0.24	0.19	0.30	0.44	0.64	0.25	0.25	0.38	0.83	0.65	0.28
(11)	Y	0.19	0.34	1.67	0.62	0.24	0.17	0.30	0.70	0.44	0.21	0.24	0.41	1.07	0.64	0.27
(12)	Y	0.16	0.27	1.42	0.43	0.18	0.17	0.23	0.33	0.40	0.19	0.17	0.30	0.77	0.52	0.21
(13)	Y	0.15	0.26	1.42	0.43	0.18	0.18	0.21	0.29	0.40	0.18	0.17	0.29	0.76	0.50	0.20
(14)	Y	0.14	0.25	1.43	0.39	0.17	0.12	0.18	0.28	0.37	0.15	0.15	0.28	0.75	0.50	0.19
																

(1/8)	Eucl.^*e*^	0.38	0.58	0.90	1.13	0.44	0.37	0.51	0.69	1.14	0.43	0.52	0.79	1.37	1.64	0.62
(2/9)	Eucl.	0.32	0.48	1.78	0.76	0.36	0.30	0.51	0.97	0.90	0.34	0.37	0.57	1.34	0.89	0.40
(3/10)	Eucl.	0.29	0.52	4.45	0.64	0.30	0.25	0.36	0.47	0.67	0.30	0.30	0.46	1.12	0.69	0.32
(4/11)	Eucl.	0.26	0.40	1.68	0.67	0.29	0.24	0.42	0.88	0.56	0.27	0.30	0.51	1.32	0.80	0.33
(5/12)	Eucl.	0.22	0.32	1.43	0.48	0.23	0.21	0.27	0.36	0.44	0.23	0.22	0.37	1.07	0.57	0.24
(6/13)	Eucl.	0.21	0.31	1.43	0.46	0.22	0.20	0.25	0.31	0.42	0.21	0.21	0.36	1.07	0.53	0.24
(7/14)	Eucl.	0.18	0.28	1.43	0.44	0.20	0.16	0.22	0.30	0.38	0.18	0.18	0.34	1.05	0.53	0.21

^*a*^See Materials and Methods for descriptions of functions according to the number; ^*b*^All values in cm; ^*c*^Root Mean Square; ^*d *^**Maximum **error of the 90^th ^percentile of spots (dropping the 10^th ^percentile of spots with the worst error). ^*e *^Euclidean distance.

### Factors other than length also contribute to migration

Spot locations in laboratory RLGS gels depend not only upon DNA sequence properties, but also upon laboratory conditions. Temperature, ionic strength of buffers, gel running times, and the physical handling of RLGS gels all affect the migration of DNA fragments. Exact spot locations on different gels vary slightly, even if the gels are produced from the same DNA specimen, although their relative positions do not change. Thus, our prediction formulas must model not only migration distance based on sequence properties, but also the effects of warping and distortion in the gel used to create the Master Profile.

Comparing the three formulas (see Materials and Methods) based on fragment length (1, 2, 3, and 8, 9, 10) there is marked improvement from the logL formula to the cubic to the quintic polynomial. Adding a linear curvature term to the cubic and quintic length polynomials improves both formulas (4, 5, 11, and 12), with the quintic polynomial retaining an advantage. Replacing curvature by the GC ratio (ratio of G or C base pairs to total fragment length) gives a slight, but non-significant, improvement (6 and 13). Fragment GC ratio is highly correlated with curvature, suggesting that these terms are interchangeable. Computing GC ratio is much simpler than computing curvature and is far preferable.

Each spot location is based on two fragments, one that migrates in the X-direction and a second that migrates in the Y-direction. The X-direction formulas discussed above depend upon the sequence of the first fragment in the pair. The last X-direction formula (7) adds a linear term proportional to the log of the length of the second sequence. This can be viewed as slightly adjusting the X-coordinate based on the Y-coordinate of the spot. Similarly, the Y-direction formulas depend upon the sequence of the second fragment in the pair. The last Y-direction formula (14) adds a linear term proportional to the log of the length of the first sequence. The last formula gives the best fit on all three gels.

**Figure 2:**
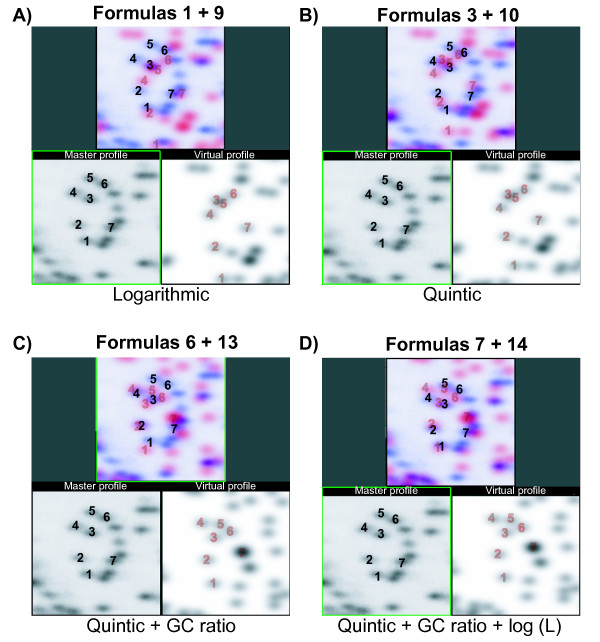
**First and second dimension migration formulas**. Master and vRLGS images for the human genome with the enzyme combination NotI-EcoRV-HinfI zoomed in on region 2D of the master profile. Spots labeled 1–7 are known sequences and are labeled on both the master and virtual profiles, with their overlap shown in the upper panels. The virtual spot labels are grey and outlined in red. **A) – D) **show the same window of the gels using the indicated migration formulas for the first and second dimensions (see materials and methods for formulas).

The improvement in prediction formula using the quintic polynomial with GC ratio and the Log of the other dimension fragment length can be easily seen by visually comparing computer generated vRLGS gels with laboratory gels. Figure 2, shows a region in section 2D of the master human NotI-EcoRV-HinfI profile and the corresponding regions in virtual profiles generated by various formulas. Spots labeled 1 through 7 are previously cloned RLGS spots allowing the correct identification of each of the corresponding virtual spots, as shown in Fig 2. The swirl shape seen in the master profile is much more evident in Fig 2C and 2D. Of particular importance are the relative positions of spots 3, 5, and 6. In Fig 2A and 2B, the position of spot 5 is below spots 3 and 6 in the virtual profiles, but in the master profile spot 5 is above spots 3 and 6. This is remedied in Fig 2C and 2D where adding in a sequence specific factor (GC ratio) corrects for the slower migration of spot 5. Finally in Fig 2D, although the absolute positions of spots 3, 4, 5, and 6 are not perfect in the virtual gel, their relative spacing is correct within a distance equal to the height of the spots.

More broadly, Table 1 shows that by using the migration formulas shown in Fig 2D, the median error between predicted position and actual position for all cloned spots is 0.06 cm in the 1^st ^dimension and 0.14 cm in the 2^nd^. To address whether these data were due merely to over-fitting of the migration formulas to the spot migration data, we randomly selected half of the cloned spots to be used to derive the migration formulas and measured the accuracy of the other half. We found a median error in the 1^st ^dimension of 0.06 cm and of 0.16 in the second dimension (data not shown), demonstrating that the data shown in Table 1 is not due to over-fitting of the data. Such improvement over previous virtual RLGS approaches dramatically improves the utility of virtual RLGS for spot identification since the error is less than half the average distance between spots. This makes it much more likely that the pattern of spots relative to each other will be faithfully preserved in the virtual image even if some small error remains in their absolute positions.

There are two confounding factors in modeling DNA fragment migration in these gels that make using a simple migration model based on the log of the fragment size insufficient. These are the physical imperfection of these gels as described above and the sequence dependent geometry of the DNA fragments. We hypothesize that the improvement from the logarithmic to cubic to quintic formulas probably reflects the better ability of the quintic polynomial to model physical gel distortion. The variability in physical gel distortion from one Master Profile to the other can be accounted for by independent derivation of the coefficients based upon known spots.

The improvements seen by adding in the GC ratio factor reflect the sequence dependent influence of DNA fragment geometry on fragment migration. Although this is an indirect measure of geometry, it performed similarly to the use of the geometry prediction program Curva. We believe that this factor is the most difficult to model properly and is the major reason some error remains in these models.

The improvement in the final formula, adding in the log of the length of the other dimension fragment, reflects the better ability of this formula to model physical gel distortion and possibly the influence of DNA fragment geometry. The fact that vertical migration in the Y-direction depends somewhat on the size of the horizontal fragment is not so surprising since vertical migration follows horizontal migration. More remarkable is that the X-location seems to depend upon the size of the vertical fragment, even though horizontal migration precedes vertical migration. We hypothesize that vertical fragments do not necessarily migrate in a perfect vertical line through a gel, but actually move slightly along the X-direction, explaining why the X-location would depend upon the vertical fragment size.

### Filtering extra spots from the vRLGS

**Figure 3:**
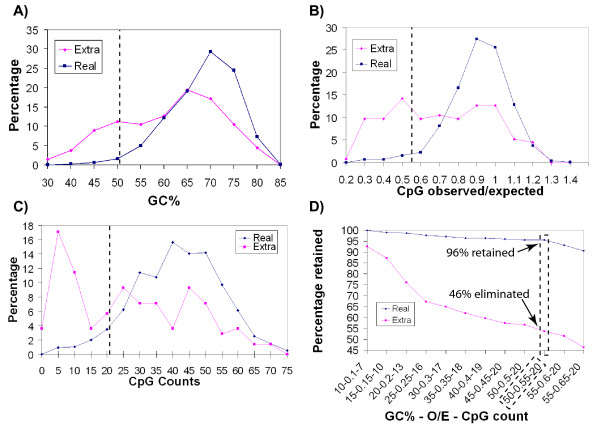
**Sequence characteristics surrounding the NotI site of extra spots and real spots**. Analysis of 200 bp + and – the NotI site for 120 extra spots and 600 real spots from the human NotI-EcoRV-HinfI RLGS profile was performed. In all four charts, data for the extra spots is shown in pink and data for real spots is shown in blue. The Y axes for all are the percentage of the class of spots showing the measure indicated on the X axis **A) **Measures of GC content. **B) **Observed/expected CpG ratio. **C) **Number of CpGs. **D) **The percentage of spots retain after eliminating all spots that fail to meet the three indicated criteria. The boxed data points show the percentage of real and extra spots retained when all spots that fail to meet all three of the following criteria are eliminate: 50%

RLGS is typically applied using a methylation sensitive landmark enzyme such as NotI or AscI, with the goal of identifying methylation differences at CpG islands between state A and state B (i.e.; normal vs. tumor, or tissue A vs. tissue B). Upon close examination of the vRLGS profile it became clear that there were significantly more virtual spots than real spots. This can be explained by the fact that certain NotI sites are methylated, even in normal tissues, and this may be tissue specific. The presence of these extra spots on the virtual profile makes the pattern correlations with the actual RLGS profile much more difficult to discern. We investigated whether a difference in the sequence surrounding the NotI sites could be found between real RLGS spots and extra spots seen only on the virtual profile. We identified 120 extra spots from the virtual profile in regions of the gel where most of the actual RLGS spots had already been identified. We compared the sequence of the NotI site +/- 200 bp (total of 400 bp) from the 120 extra virtual spots to 600 cloned and confirmed actual RLGS spots. Figure 3 shows the distributions of GC%, observed over expected CpG ratio, and CpG count in the 120 extra virtual spots and the 600 actual RLGS spots. We tested various combinations of these three factors (Fig 3D) that could best distinguish the two groups in order to maximize the number of extra spots identified, yet minimize the number of actual spots identified. We found that if we filtered out NotI sites whose surrounding 400 bp of sequence had less than 50% GC, a CpG ratio of less than 0.55, and a CpG count of less than 20, we could eliminate 46% of our 120 extra virtual spots, yet retain 96% of our 600 actual spots.

**Figure 4:**
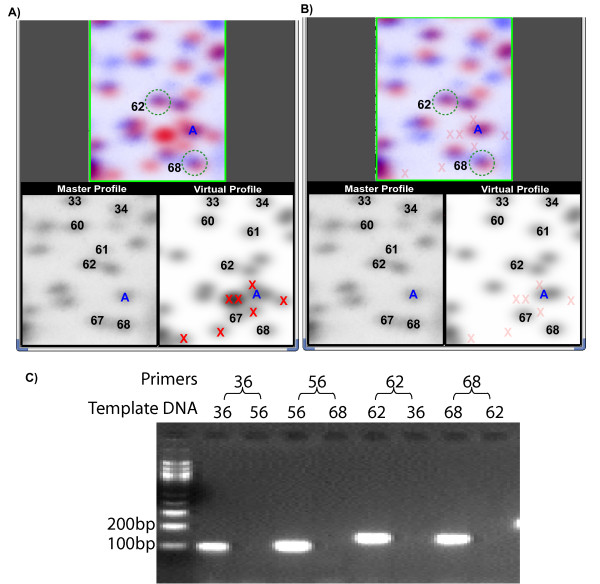
**vRLGS spot candidate identification and confirmation**. Human NotI-EcoRV-HinfI profiles zoomed into section 3D. Identified spots are labeled on the lower panels for A) and B), with the upper panels showing the overlap of the actual and virtual spots of interest – 62 and 68 – circled. All other labeled spots were previously identified. **A) **The vRLGS image with the extra spot filtering ***off***. Extra spots are indicated with red Xs. Spot A is a spot of interest for which the presence of extra spots makes the selection of the best candidate sequence difficult. **B) **The same vRLGS image with the extra spot filtering ***on***. Faded red Xs indicate the positions the extra spot occupied in A), but are not seen with the filtering ***on***. The candidate sequence for spot of interest A becomes immediately obvious. **C) **Example of PCR candidate sequence confirmation. DNA was eluted from the spots indicate as template DNA for PCR using primers derived from the candidate sequence for the spots of interest. In the last two lanes, primers for the candidate sequence for spot 68 were used to amplify DNA eluted from spot 62 and spot 68. Only the when the DNA eluted from spot 68 was used as template was a band observed. This is confirmation that the sequence predicted by the vRLGS in A) and B) is correct for spot 68. The same is true for spots 36 and 56 (not shown in A) and B)), and 62.

The results of this filtering can be seen in figure 4A and 4B. In each, the lower left is the master RLGS profile zoomed in to section 3D, with the corresponding region of the virtual gel shown in the right panel. The numbered spots are all identified and confirmed and are therefore noted on the virtual profile. Spot A (Fig 4A) represents a spot for which identification of a good candidate sequence is extremely difficult because of extra spots (indicated with red Xs) seen on the virtual profile complicating the virtual pattern near spot A. However, when we applied the filter described above to remove all extra spots based on the sequence environment of the NotI site, the best candidate sequence for spot A became immediately obvious (Fig 4B).

**Figure 5:**
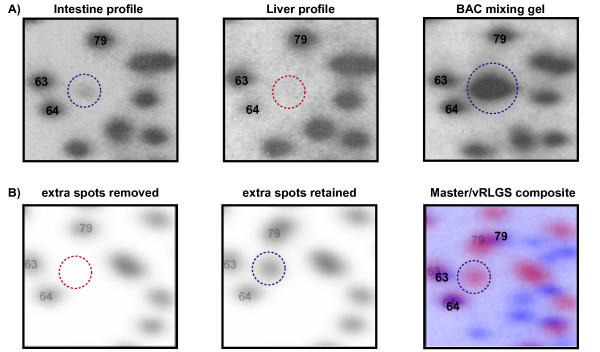
**Application of vRLGS to the mouse NotI-EcoRV-HinfI profile to identify a tissue specific methylation locus and confirmation by BAC clone mixing gel**. Mouse RLGS profiles zoomed in on section 2F. **A) **Actual mouse RLGS profiles from intestine and liver. The circled spot is present in the intestine profile but absent from the liver profile. **B) **vRLGS images of the same region with the extra spot filtering turned ***on ***in the first panel, but turned ***off ***in the second panel. The third panel shows the overlay of the mouse master profile where the spot of interest is not seen and the vRLGS profile with the extra spot filtering turned ***off***. The intestine specific spot of interest is only seen on the vRLGS profile in the extra spot filtering is turned ***off***. This candidate sequence from the vRLGS profile was confirmed to be correct by performing a BAC clone mixing gels shown in the third panel of A) where the spot of interest is greatly enhanced by the radio-labeled BAC.

We note that the filtering criteria identified are similar to criteria used to define CpG islands. We find that NotI sites in regions that look less like CpG islands are more likely to be extra spots. This is consistent with the idea that CpG islands are protected from methylation in normal cells in most cases but CpG dinucleotides outside CpG islands are highly methylated, and also explains why even though only 89% of NotI sites in the human genome reside in CpG islands, 96% of identified RLGS spots are in CpG islands (Table 2). Interestingly, as described below and shown in Figure 5, it is often true that RLGS loci found to exhibit tissue specific methylation arise from NotI sites that we would filter out (Fig 5B), and are therefore less "CpG island-like." It was recently found that there are many regions of hypomethylation in mouse testis DNA compared to DNA in somatic tissues and that these chromosomal regions are defined by lower GC content [27]. These observations suggest that there may be a relationship between the potential of a locus to exhibit tissue specific methylation and the "CpG island-like" characteristics of the sequence.

**Table 2:** Summary of RLGS spot characteristics

	**Human NotI-EcoRV-HinfI** **(n = 795)**		**Mouse NotI-EcoRV-HinfI** **(n = 530)**	
**CpG island**	760	96%	459	87%
				
**Gene Homology**	745	94%	503	95%
**Intergenic**	49	6%	27	5%
				
**5' end**	569	76%	439	87%
**Non-5' end**	176	24%	64	13%

### Efficacy of vRLGS for novel spot identification

Using the human NotI-EcoRV-HinfI vRLGS profile produced with the migration algorithms derived from 300 spots cloned from the library mixing gel approach and incorporating DNA curvature and GC ratio, we attempted to identify 337 unknown RLGS spots. We identified a top candidate sequence for each spot and designed PCR primers that fall within the 2^nd ^dimension fragment. For 20 spots, we chose 2 candidate sequences to test because one candidate appeared only slightly better than the other. We then used the primers for the candidate sequence to amplify DNA eluted from the spot in question, as well as a nearby spot. Accurate prediction of a spot sequence was determined if a strong PCR product was obtained when that spot was used as template, but no product or a very weak product was detected using a nearby spot as template.

Figure 4 shows an example of the vRLGS spot sequence verification experiment. We predicted the sequence of spots 36, 56, (not shown in this window) 62, and 68 from section 3D of the master profile based on the virtual spots indicated (Fig 4B). In all four cases, the PCR experiment described above showed a strong band when the primers from the predicted sequence were used with template DNA from the spot of interest, but no band when DNA from a nearby spot was used as template. This PCR confirmation experiment was successfully implemented for 293 of the candidate sequences and 246 were found to be correct by this method while 47 candidates were found to be incorrect. For the remaining 44 spots, the PCR experiment was inconclusive either due to a failed PCR reaction even in total genomic DNA, or because a positive band was found in both the expected spot DNA as well as the negative control spot DNA. For these, we tested the accuracy of the candidates either with BAC mixing gels or with mixing gels of the NotI-EcoRV fragments PCR cloned from genomic DNA (materials and methods). 30 of the 44 tested by this method were correct. For the 20 spots where we tested 2 candidate sequences, we found a clear result of only one of the two sequences correct in each case. Overall, 276 of the 337 RLGS spots we attempted to clone by the vRLGS approach were confirmed to be correct.

### Application of vRLGS to other genomes and enzyme combinations

We have demonstrated excellent utility and pattern correlation of the vRLGS profile with the human NotI-EcoRV-HinfI enzyme combination from which the migration algorithms and extra spot filtering were derived. To address whether these improvements to vRLGS were based on sound principles or were simply due to over fitting of the data, we applied the same migration factors to create a human AscI-EcoRV-HinfI vRLGS profile (Additional file 1). In addition we created vRLGS profiles for the mouse genome using the NotI-EcoRV-HinfI (Additional file 2). We used cloned spots as anchor points for each and calculated the formula coefficients as described in the materials and methods. Table 1 shows the error of the prediction formulas for the cloned spots in the two human profiles and the NotI-EcoRV-HinfI mouse profile with 795, 133, and 530 spots cloned, respectively. These data demonstrate that the migration algorithms developed for the human NotI-EcoRV-HinfI profile incorporating fragment length as well as information about the GC content of each fragment are based on sound principles and can be broadly applied to different enzyme combinations and genomes.

As a further test of the utility of the vRLGS system in the mouse genome, we used vRLGS to identify the sequence of 126 spots from the mouse NotI-EcoRV-HinfI master profile. In addition, turning on or off the filtering out of extra spots can allow for use of vRLGS to identify tissue specific methylation spots. Figure 5 shows section 2F from the mouse NotI-EcoRV-HinfI profile with spots 63, 64, and 79 previously identified and the indicated spot not present in the master profile or in a mouse liver profile. This spot was, however, found on a mouse intestine profile (Fig 5A first panel). When using the vRLGS profile where we filtered out extra spots, the intestine specific spot of interest was not present on the virtual profile (Fig 5B first panel), however, when we did not filter out the extra spots, the virtual spot was present (Fig 5B second panel). The third panel of Fig 5B shows the composite image of the vRLGS profile with the extra spots retained aligned with the master profile and the candidate virtual spot for the intestine specific spot of interest indicated. We confirmed the correct identification of this intestine specific spot by BAC clone mixing gel (Fig 5A third panel). These data demonstrate that although in most cases filtering out the extra spots helps with the cloning of a particular spot of interest by reducing the complexity of the virtual pattern (Fig 4), for some specific spots in special situations (i.e. – tissue specific methylation, hypomethylation in cancer genomes) it is necessary to keep the extra spots in order to identify the spot of interest.

### Virtual spot identification of genetically mapped loci

As a further test of the general applicability of our vRLGS system, we applied it to the mouse genome for two enzyme combinations where significant genetic mapping of RLGS spots had been previously published. Importantly, these were completely naïve data sets to test the vRLGS application since we had no previously cloned spots to use to derive coefficients for the DNA fragment migration formulas. We used the exact same formulas that were used for the mouse NotI-EcoRV-HinfI vRLGS profiles, again demonstrating that the accuracy of this system is due to the migration formulas being based on sound principles.

**Figure 6:**
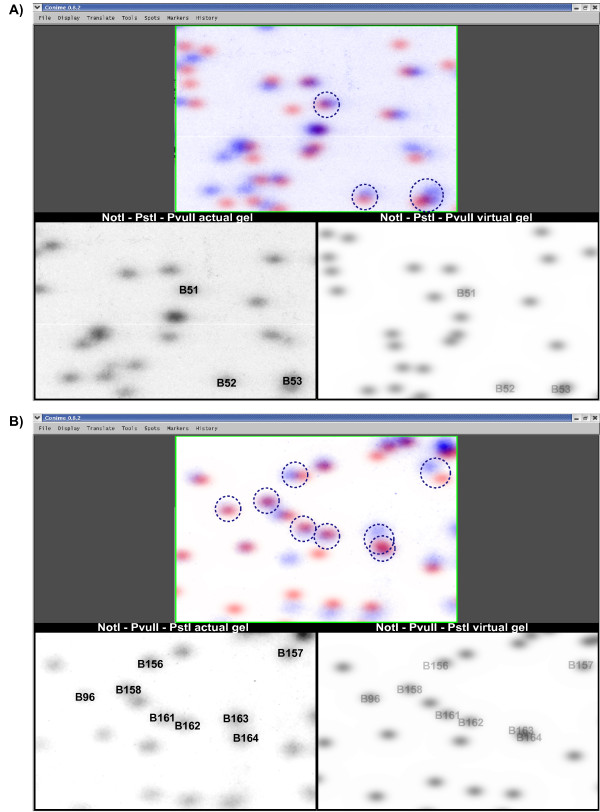
**Application of vRLGS to the mouse genome with alternative enzyme combinations**. **A) **A mouse vRLGS profile was generated for the enzyme combination NotI-PstI-PvuII (lower right) and compared with an actual gel (lower left). Spots of interest, previously mapped in BXD recombinant inbred strains are labeled on the actual gel and candidate vRLGS spots are labeled that are located in the same genomic region as the genetically mapped spots. The circles in the upper panel indicate the overlap between the actual and virtual spots of interest. **B) **Same as in A) except using the enzyme combination NotI-PvuII-PstI. The spots of interest are genetically mapped in a BSS interspecific backcross.

A set of 195 RLGS spots from the enzyme combination NotI-PstI-PvuII that vary between C57BL/6J and DBA/2J have been previously mapped using recombinant inbred strains (BXD) [28]. For the NotI-PvuII-PstI enzyme combination, a set of 575 RLGS loci were mapped in a C57BL/6 × M. Spretus interspecific backcross (BSS) [29]. We aligned the vRLGS profiles for both enzyme combinations with actual gels as shown in Figure 6. Excellent pattern matching was observed and we attempted to identify a total of 263 mapped C57BL/6 spots on the vRLGS profiles. We identified obvious candidate virtual spots for 60/96 spots mapped in the BXD data (Fig 6A) and 140/167 spots mapped with the BSS data (Fig 6B) for a total of 200 C57BL/6 spot candidate virtual sequences. We then looked at the position of the NotI site in the mouse genome for each of the candidate sequences and compared that with the genetic mapping data available for 184 of these RLGS spots (mapping data was not found for 16 spots). We found that 92% of the virtual RLGS spot candidate sequences were consistent with the genetic mapping BXD data and 98% were consistent with the genetic mapping BSS data (Additional files 3 and 4, respectively). These data provide an independent proof of the accuracy and utility of our vRLGS system and demonstrate that this is not merely due to over fitting of the migration algorithms.

### RLGS spot sequence characteristics

Through a combination of methods including boundary library mixing gels, BAC clone mixing gels, and vRLGS we have identified and confirmed the sequence of 795 human NotI-EcoRV-HinfI spots (Additional file 5) and 530 mouse NotI-EcoRV-HinfI spots (Additional file 6). To study the sequence characteristics and genomic context of each of these RLGS spots, we looked at the NotI site plus or minus 200 bp and asked if this 400 bp sequence fell within a CpG island and if the CpG island was within 5 kb of the transcriptional start site of a known gene or mRNA sequence (Table 2). We found that 96% of the human RLGS spots and 87% of the mouse RLGS spots were located in CpG islands. Greater than 90% of these were associated with genes in both genomes and were found at the 5' ends of genes for 76% in the human genome and 87% in the mouse genome. These data confirm the findings of multiple smaller data sets demonstrating the strong bias of RLGS data to focus on CpG islands in the regulatory regions of genes where the best understood biological consequences of aberrant hypermethylation arise.

## Conclusion

We have presented a novel bioinformatics tool to rapidly identify RLGS spot sequences by creating a virtual RLGS profile to connect the position of spots with genomic sequences. This system of vRLGS builds upon previous systems by incorporating migration algorithms for both dimension that include consideration of the sequence characteristics of every restriction fragment, as well as the size. In addition, this vRLGS system predicts which virtual spots will not be present on most normal profiles and can filter these extra spots out, or leave them in the profile if the research being done dictates a need to identify such loci.

The accuracy and utility of this vRLGS system was demonstrated on both the human and mouse genomes and for multiple enzyme combinations, demonstrating the broad applicability of the principals behind the migration algorithms. Although this system is still not able to perfectly mimic an actual gel, the data demonstrate an excellent ability to easily identify the most likely candidate sequence for a spot of interest in most cases. As more RLGS spot clone information becomes confirmed, the new data can be incorporated into the migration algorithms to improve the overall fitting of the virtual profile to the actual profile.

The reporting of the confirmed identification of 795 human RLGS spots and 530 mouse RLGS spots provides a valuable tool for researchers using RLGS on human samples or in mouse models. The characteristics of these identified RLGS spots confirm the value of the RLGS approach to focus aberrant methylation studies on CpG islands. The vRLGS tool can now be used to identify the top candidate sequences for RLGS spots whose pattern of methylation in cancers is of interest. Confirmation of the accuracy of the prediction allows for rapid identification of novel targets of CpG island hypermethylation.

## Methods

The software we used for vRLGS software is divided into four components: enzyme simulation, methylation prediction, location prediction and vRLGS image generation. We also used a software tool called Conime for visualizing vRLGs images and comparing them with laboratory RLGS gels. Dr. Stanislav Volik UCSF wrote the enzyme simulation software, rlgs.pl, although we made extensive modifications. Dr. Ping Liang at Roswell Park Cancer Institute developed the methylation prediction module. All other software, including Conime, was developed at The Ohio State University (OSU). Perl scripts are available on the web [30].

### Enzyme simulation (rlgs.pl)

In laboratory RLGS, three enzymes digest genomic DNA into fragments for gel electrophoresis. The program rlgs.pl, written in the programming language Perl, simulates this process using pattern matching on DNA sequences from genome databases. Input to rlgs.pl is a fasta file containing a chromosome DNA sequence and three strings representing the recognition sites of the three enzymes. The chromosome sequence and the recognition sites are represented as strings of the characters A, C, G, T. rlgs.pl splits the chromosome sequence at substrings which match the first recognition site, creating a set Frag1 of subsequences. The program then splits each subsequence at substrings which match the second recognition site creating set Frag2. Finally, it splits each resulting subsequence at substrings which match the third recognition site creating set Frag3.

Each fragment has two cleavage sites, one at each endpoint. (The two exceptions are the fragments corresponding from the very first and the very last subsequences of the chromosome.) A spot which appears on a laboratory RLGS gel is created by a radioactive nucleotide bound to a first enzyme cleavage site. Thus, rlgs.pl discards any subsequences which do not contain a first enzyme cleavage site.

RLGS fragments are separated along the first dimension after the application of the second enzyme. The third enzyme is applied after this first dimension separation and the resulting fragments are then separated in the second dimension. Thus two fragments determine the location of each spot on the gel, one from before and one from after application of the third enzyme. rlgs.pl produces pairs of subsequences corresponding to these two fragments for each spot. The first element of each pair is from set Frag2 while the second is from set Frag3. The second element is a subsequence of the first. The pairs of subsequences are used to determine the spots appearing on the vRLGS image as described below.

If a fragment is too long or too short, then it will fail to migrate or will migrate all the way across the gel. Such fragments do not create spots on the gel. We eliminated any fragment pairs whose first fragment contained less than 400 or more than 8000 base pairs or whose second fragment contained less than 100 or more than 3000 base pairs.

### Methylation prediction

Methylation sensitive enzymes cut DNA only at unmethylated binding sites. However, genome databases do not indicate whether a binding site is methylated or unmethylated. Thus, rlgs.pl treats all binding sites as unmethylated, creating many fragment pairs which do not correspond to laboratory RLGS spots. Eliminating such spots from the vRLGS image improves the quality and accuracy of our vRLGS image. We identified 120 extra spots from the virtual profile in regions of the gel where most of the actual RLGS spots had already been identified. We compared the sequence of +/-200 base pairs around each spot's NotI site with the sequence around the NotI sites of the 600 cloned and confirmed actual RGLS spots and predict the methylation status of the NotI sites as described in the Results and Discussion.

### Location prediction

The most critical component in virtual RLGS is the ability to accurately predict the location of each spot on the gel. This location is determined by two fragments, one from before and one from after the application of the third enzyme. rlgs7.pl produces pairs of subsequences corresponding to RLGS fragment pairs.

fff2cnm.pl is a Perl program which converts pairs of DNA sequences into spot locations on a virtual RLGS image. We experimented with a number of different prediction formulas and report on seven of them here. These formulas depended on three parameters: DNA sequence length, curvature and GC ratio. The DNA sequence length is simply the number of base pairs in the sequence. The curvature is the curvature of the DNA fragment. We used the CURVA [31] software to calculate a sequence-dependent spatial trajectory of the DNA molecule and respective curvature distribution therein for each virtual RLGS restriction fragment. The curvature in the position "n" is defined as normalized reciprocal radius of the arc approximating the arc-size DNA fragment centered in the position "n". The GC ratio is the percentage of G's and C's in the DNA sequence.

RLGS images are usually oriented so that DNA fragments migrate from right to left along the x-axis and then from top to bottom along the y-axis. Thus the formulas predict each spot's distance from the upper-right hand corner of the gel.

The formulas for predicting the horizontal distance from the right edge of the gel are:

1. Log: a_1 _+ a_2 _log(L_1 _+ a_3_)

2. Cubic polynomial: a_0 _+ a_1 _L_1 _+ a_2 _L12
 MathType@MTEF@5@5@+=feaafiart1ev1aaatCvAUfKttLearuWrP9MDH5MBPbIqV92AaeXatLxBI9gBaebbnrfifHhDYfgasaacPC6xNi=xH8viVGI8Gi=hEeeu0xXdbba9frFj0xb9qqpG0dXdb9aspeI8k8fiI+fsY=rqGqVepae9pg0db9vqaiVgFr0xfr=xfr=xc9adbaqaaeGacaGaaiaabeqaaeqabiWaaaGcbaGaeeitaW0aa0baaSqaaiabigdaXaqaaiabikdaYaaaaaa@2F01@ + a_3 _L13
 MathType@MTEF@5@5@+=feaafiart1ev1aaatCvAUfKttLearuWrP9MDH5MBPbIqV92AaeXatLxBI9gBaebbnrfifHhDYfgasaacPC6xNi=xH8viVGI8Gi=hEeeu0xXdbba9frFj0xb9qqpG0dXdb9aspeI8k8fiI+fsY=rqGqVepae9pg0db9vqaiVgFr0xfr=xfr=xc9adbaqaaeGacaGaaiaabeqaaeqabiWaaaGcbaGaeeitaW0aa0baaSqaaiabigdaXaqaaiabiodaZaaaaaa@2F03@

3. Quintic polynomial: a_0 _+ a_1 _L_1 _+ a_2 _L12
 MathType@MTEF@5@5@+=feaafiart1ev1aaatCvAUfKttLearuWrP9MDH5MBPbIqV92AaeXatLxBI9gBaebbnrfifHhDYfgasaacPC6xNi=xH8viVGI8Gi=hEeeu0xXdbba9frFj0xb9qqpG0dXdb9aspeI8k8fiI+fsY=rqGqVepae9pg0db9vqaiVgFr0xfr=xfr=xc9adbaqaaeGacaGaaiaabeqaaeqabiWaaaGcbaGaeeitaW0aa0baaSqaaiabigdaXaqaaiabikdaYaaaaaa@2F01@ + a_3 _L13
 MathType@MTEF@5@5@+=feaafiart1ev1aaatCvAUfKttLearuWrP9MDH5MBPbIqV92AaeXatLxBI9gBaebbnrfifHhDYfgasaacPC6xNi=xH8viVGI8Gi=hEeeu0xXdbba9frFj0xb9qqpG0dXdb9aspeI8k8fiI+fsY=rqGqVepae9pg0db9vqaiVgFr0xfr=xfr=xc9adbaqaaeGacaGaaiaabeqaaeqabiWaaaGcbaGaeeitaW0aa0baaSqaaiabigdaXaqaaiabiodaZaaaaaa@2F03@ + a_4 _L14
 MathType@MTEF@5@5@+=feaafiart1ev1aaatCvAUfKttLearuWrP9MDH5MBPbIqV92AaeXatLxBI9gBaebbnrfifHhDYfgasaacPC6xNi=xH8viVGI8Gi=hEeeu0xXdbba9frFj0xb9qqpG0dXdb9aspeI8k8fiI+fsY=rqGqVepae9pg0db9vqaiVgFr0xfr=xfr=xc9adbaqaaeGacaGaaiaabeqaaeqabiWaaaGcbaGaeeitaW0aa0baaSqaaiabigdaXaqaaiabisda0aaaaaa@2F05@ + a_5 _L15
 MathType@MTEF@5@5@+=feaafiart1ev1aaatCvAUfKttLearuWrP9MDH5MBPbIqV92AaeXatLxBI9gBaebbnrfifHhDYfgasaacPC6xNi=xH8viVGI8Gi=hEeeu0xXdbba9frFj0xb9qqpG0dXdb9aspeI8k8fiI+fsY=rqGqVepae9pg0db9vqaiVgFr0xfr=xfr=xc9adbaqaaeGacaGaaiaabeqaaeqabiWaaaGcbaGaeeitaW0aa0baaSqaaiabigdaXaqaaiabiwda1aaaaaa@2F07@

4. Cubic & curvature: a_0 _+ a_1 _L_1 _+ a_2 _L12
 MathType@MTEF@5@5@+=feaafiart1ev1aaatCvAUfKttLearuWrP9MDH5MBPbIqV92AaeXatLxBI9gBaebbnrfifHhDYfgasaacPC6xNi=xH8viVGI8Gi=hEeeu0xXdbba9frFj0xb9qqpG0dXdb9aspeI8k8fiI+fsY=rqGqVepae9pg0db9vqaiVgFr0xfr=xfr=xc9adbaqaaeGacaGaaiaabeqaaeqabiWaaaGcbaGaeeitaW0aa0baaSqaaiabigdaXaqaaiabikdaYaaaaaa@2F01@ + a_3 _L13
 MathType@MTEF@5@5@+=feaafiart1ev1aaatCvAUfKttLearuWrP9MDH5MBPbIqV92AaeXatLxBI9gBaebbnrfifHhDYfgasaacPC6xNi=xH8viVGI8Gi=hEeeu0xXdbba9frFj0xb9qqpG0dXdb9aspeI8k8fiI+fsY=rqGqVepae9pg0db9vqaiVgFr0xfr=xfr=xc9adbaqaaeGacaGaaiaabeqaaeqabiWaaaGcbaGaeeitaW0aa0baaSqaaiabigdaXaqaaiabiodaZaaaaaa@2F03@ + b C_1_

5. Quintic & curvature: a_0 _+ a_1 _L_1 _+ a_2 _L12
 MathType@MTEF@5@5@+=feaafiart1ev1aaatCvAUfKttLearuWrP9MDH5MBPbIqV92AaeXatLxBI9gBaebbnrfifHhDYfgasaacPC6xNi=xH8viVGI8Gi=hEeeu0xXdbba9frFj0xb9qqpG0dXdb9aspeI8k8fiI+fsY=rqGqVepae9pg0db9vqaiVgFr0xfr=xfr=xc9adbaqaaeGacaGaaiaabeqaaeqabiWaaaGcbaGaeeitaW0aa0baaSqaaiabigdaXaqaaiabikdaYaaaaaa@2F01@ + a_3 _L13
 MathType@MTEF@5@5@+=feaafiart1ev1aaatCvAUfKttLearuWrP9MDH5MBPbIqV92AaeXatLxBI9gBaebbnrfifHhDYfgasaacPC6xNi=xH8viVGI8Gi=hEeeu0xXdbba9frFj0xb9qqpG0dXdb9aspeI8k8fiI+fsY=rqGqVepae9pg0db9vqaiVgFr0xfr=xfr=xc9adbaqaaeGacaGaaiaabeqaaeqabiWaaaGcbaGaeeitaW0aa0baaSqaaiabigdaXaqaaiabiodaZaaaaaa@2F03@ + a_4 _L14
 MathType@MTEF@5@5@+=feaafiart1ev1aaatCvAUfKttLearuWrP9MDH5MBPbIqV92AaeXatLxBI9gBaebbnrfifHhDYfgasaacPC6xNi=xH8viVGI8Gi=hEeeu0xXdbba9frFj0xb9qqpG0dXdb9aspeI8k8fiI+fsY=rqGqVepae9pg0db9vqaiVgFr0xfr=xfr=xc9adbaqaaeGacaGaaiaabeqaaeqabiWaaaGcbaGaeeitaW0aa0baaSqaaiabigdaXaqaaiabisda0aaaaaa@2F05@ + a_5 _L15
 MathType@MTEF@5@5@+=feaafiart1ev1aaatCvAUfKttLearuWrP9MDH5MBPbIqV92AaeXatLxBI9gBaebbnrfifHhDYfgasaacPC6xNi=xH8viVGI8Gi=hEeeu0xXdbba9frFj0xb9qqpG0dXdb9aspeI8k8fiI+fsY=rqGqVepae9pg0db9vqaiVgFr0xfr=xfr=xc9adbaqaaeGacaGaaiaabeqaaeqabiWaaaGcbaGaeeitaW0aa0baaSqaaiabigdaXaqaaiabiwda1aaaaaa@2F07@ + b C_1_

6. Quintic & GC ratio: a_0 _+ a_1 _L_1 _+ a_2 _L12
 MathType@MTEF@5@5@+=feaafiart1ev1aaatCvAUfKttLearuWrP9MDH5MBPbIqV92AaeXatLxBI9gBaebbnrfifHhDYfgasaacPC6xNi=xH8viVGI8Gi=hEeeu0xXdbba9frFj0xb9qqpG0dXdb9aspeI8k8fiI+fsY=rqGqVepae9pg0db9vqaiVgFr0xfr=xfr=xc9adbaqaaeGacaGaaiaabeqaaeqabiWaaaGcbaGaeeitaW0aa0baaSqaaiabigdaXaqaaiabikdaYaaaaaa@2F01@ + a_3 _L13
 MathType@MTEF@5@5@+=feaafiart1ev1aaatCvAUfKttLearuWrP9MDH5MBPbIqV92AaeXatLxBI9gBaebbnrfifHhDYfgasaacPC6xNi=xH8viVGI8Gi=hEeeu0xXdbba9frFj0xb9qqpG0dXdb9aspeI8k8fiI+fsY=rqGqVepae9pg0db9vqaiVgFr0xfr=xfr=xc9adbaqaaeGacaGaaiaabeqaaeqabiWaaaGcbaGaeeitaW0aa0baaSqaaiabigdaXaqaaiabiodaZaaaaaa@2F03@ + a_4 _L14
 MathType@MTEF@5@5@+=feaafiart1ev1aaatCvAUfKttLearuWrP9MDH5MBPbIqV92AaeXatLxBI9gBaebbnrfifHhDYfgasaacPC6xNi=xH8viVGI8Gi=hEeeu0xXdbba9frFj0xb9qqpG0dXdb9aspeI8k8fiI+fsY=rqGqVepae9pg0db9vqaiVgFr0xfr=xfr=xc9adbaqaaeGacaGaaiaabeqaaeqabiWaaaGcbaGaeeitaW0aa0baaSqaaiabigdaXaqaaiabisda0aaaaaa@2F05@ + a_5 _L15
 MathType@MTEF@5@5@+=feaafiart1ev1aaatCvAUfKttLearuWrP9MDH5MBPbIqV92AaeXatLxBI9gBaebbnrfifHhDYfgasaacPC6xNi=xH8viVGI8Gi=hEeeu0xXdbba9frFj0xb9qqpG0dXdb9aspeI8k8fiI+fsY=rqGqVepae9pg0db9vqaiVgFr0xfr=xfr=xc9adbaqaaeGacaGaaiaabeqaaeqabiWaaaGcbaGaeeitaW0aa0baaSqaaiabigdaXaqaaiabiwda1aaaaaa@2F07@ + b GC_1_

7. Quintic & L_2_ & GC ratio: a_0 _+ a_1 _L_1 _+ a_2 _L12
 MathType@MTEF@5@5@+=feaafiart1ev1aaatCvAUfKttLearuWrP9MDH5MBPbIqV92AaeXatLxBI9gBaebbnrfifHhDYfgasaacPC6xNi=xH8viVGI8Gi=hEeeu0xXdbba9frFj0xb9qqpG0dXdb9aspeI8k8fiI+fsY=rqGqVepae9pg0db9vqaiVgFr0xfr=xfr=xc9adbaqaaeGacaGaaiaabeqaaeqabiWaaaGcbaGaeeitaW0aa0baaSqaaiabigdaXaqaaiabikdaYaaaaaa@2F01@ + a_3 _L13
 MathType@MTEF@5@5@+=feaafiart1ev1aaatCvAUfKttLearuWrP9MDH5MBPbIqV92AaeXatLxBI9gBaebbnrfifHhDYfgasaacPC6xNi=xH8viVGI8Gi=hEeeu0xXdbba9frFj0xb9qqpG0dXdb9aspeI8k8fiI+fsY=rqGqVepae9pg0db9vqaiVgFr0xfr=xfr=xc9adbaqaaeGacaGaaiaabeqaaeqabiWaaaGcbaGaeeitaW0aa0baaSqaaiabigdaXaqaaiabiodaZaaaaaa@2F03@ + a_4 _L14
 MathType@MTEF@5@5@+=feaafiart1ev1aaatCvAUfKttLearuWrP9MDH5MBPbIqV92AaeXatLxBI9gBaebbnrfifHhDYfgasaacPC6xNi=xH8viVGI8Gi=hEeeu0xXdbba9frFj0xb9qqpG0dXdb9aspeI8k8fiI+fsY=rqGqVepae9pg0db9vqaiVgFr0xfr=xfr=xc9adbaqaaeGacaGaaiaabeqaaeqabiWaaaGcbaGaeeitaW0aa0baaSqaaiabigdaXaqaaiabisda0aaaaaa@2F05@ + a_5 _L15
 MathType@MTEF@5@5@+=feaafiart1ev1aaatCvAUfKttLearuWrP9MDH5MBPbIqV92AaeXatLxBI9gBaebbnrfifHhDYfgasaacPC6xNi=xH8viVGI8Gi=hEeeu0xXdbba9frFj0xb9qqpG0dXdb9aspeI8k8fiI+fsY=rqGqVepae9pg0db9vqaiVgFr0xfr=xfr=xc9adbaqaaeGacaGaaiaabeqaaeqabiWaaaGcbaGaeeitaW0aa0baaSqaaiabigdaXaqaaiabiwda1aaaaaa@2F07@ + b_0 _Log(L_2_) + b_1 _GC_1_

where L_1_, C_1 _and GC_1 _are the length, curvature and GC ratio of the first sequence in the pair and L_2 _is the length of the second sequence.

The formulas for predicting the vertical distance from the top edge of the gel are similar to the previous formulas, but the roles of L_1 _and L_2 _are interchanged:

8. Log: a_1 _+ a_2 _log(L_2 _+ a_3_)

9. Cubic polynomial: a_0 _+ a_1 _L_2 _+ a_2 _L22
 MathType@MTEF@5@5@+=feaafiart1ev1aaatCvAUfKttLearuWrP9MDH5MBPbIqV92AaeXatLxBI9gBaebbnrfifHhDYfgasaacPC6xNi=xH8viVGI8Gi=hEeeu0xXdbba9frFj0xb9qqpG0dXdb9aspeI8k8fiI+fsY=rqGqVepae9pg0db9vqaiVgFr0xfr=xfr=xc9adbaqaaeGacaGaaiaabeqaaeqabiWaaaGcbaGaeeitaW0aa0baaSqaaiabikdaYaqaaiabikdaYaaaaaa@2F03@ + a_3 _L23
 MathType@MTEF@5@5@+=feaafiart1ev1aaatCvAUfKttLearuWrP9MDH5MBPbIqV92AaeXatLxBI9gBaebbnrfifHhDYfgasaacPC6xNi=xH8viVGI8Gi=hEeeu0xXdbba9frFj0xb9qqpG0dXdb9aspeI8k8fiI+fsY=rqGqVepae9pg0db9vqaiVgFr0xfr=xfr=xc9adbaqaaeGacaGaaiaabeqaaeqabiWaaaGcbaGaeeitaW0aa0baaSqaaiabikdaYaqaaiabiodaZaaaaaa@2F05@

10. Quintic polynomial: a_0 _+ a_1 _L_1 _+ a_2 _L22
 MathType@MTEF@5@5@+=feaafiart1ev1aaatCvAUfKttLearuWrP9MDH5MBPbIqV92AaeXatLxBI9gBaebbnrfifHhDYfgasaacPC6xNi=xH8viVGI8Gi=hEeeu0xXdbba9frFj0xb9qqpG0dXdb9aspeI8k8fiI+fsY=rqGqVepae9pg0db9vqaiVgFr0xfr=xfr=xc9adbaqaaeGacaGaaiaabeqaaeqabiWaaaGcbaGaeeitaW0aa0baaSqaaiabikdaYaqaaiabikdaYaaaaaa@2F03@ + a_3 _L23
 MathType@MTEF@5@5@+=feaafiart1ev1aaatCvAUfKttLearuWrP9MDH5MBPbIqV92AaeXatLxBI9gBaebbnrfifHhDYfgasaacPC6xNi=xH8viVGI8Gi=hEeeu0xXdbba9frFj0xb9qqpG0dXdb9aspeI8k8fiI+fsY=rqGqVepae9pg0db9vqaiVgFr0xfr=xfr=xc9adbaqaaeGacaGaaiaabeqaaeqabiWaaaGcbaGaeeitaW0aa0baaSqaaiabikdaYaqaaiabiodaZaaaaaa@2F05@ + a_4 _L24
 MathType@MTEF@5@5@+=feaafiart1ev1aaatCvAUfKttLearuWrP9MDH5MBPbIqV92AaeXatLxBI9gBaebbnrfifHhDYfgasaacPC6xNi=xH8viVGI8Gi=hEeeu0xXdbba9frFj0xb9qqpG0dXdb9aspeI8k8fiI+fsY=rqGqVepae9pg0db9vqaiVgFr0xfr=xfr=xc9adbaqaaeGacaGaaiaabeqaaeqabiWaaaGcbaGaeeitaW0aa0baaSqaaiabikdaYaqaaiabisda0aaaaaa@2F07@ + a_5 _L25
 MathType@MTEF@5@5@+=feaafiart1ev1aaatCvAUfKttLearuWrP9MDH5MBPbIqV92AaeXatLxBI9gBaebbnrfifHhDYfgasaacPC6xNi=xH8viVGI8Gi=hEeeu0xXdbba9frFj0xb9qqpG0dXdb9aspeI8k8fiI+fsY=rqGqVepae9pg0db9vqaiVgFr0xfr=xfr=xc9adbaqaaeGacaGaaiaabeqaaeqabiWaaaGcbaGaeeitaW0aa0baaSqaaiabikdaYaqaaiabiwda1aaaaaa@2F09@

11. Cubic & curvature: a_0 _+ a_1 _L_2 _+ a_2 _L22
 MathType@MTEF@5@5@+=feaafiart1ev1aaatCvAUfKttLearuWrP9MDH5MBPbIqV92AaeXatLxBI9gBaebbnrfifHhDYfgasaacPC6xNi=xH8viVGI8Gi=hEeeu0xXdbba9frFj0xb9qqpG0dXdb9aspeI8k8fiI+fsY=rqGqVepae9pg0db9vqaiVgFr0xfr=xfr=xc9adbaqaaeGacaGaaiaabeqaaeqabiWaaaGcbaGaeeitaW0aa0baaSqaaiabikdaYaqaaiabikdaYaaaaaa@2F03@ + a_3 _L23
 MathType@MTEF@5@5@+=feaafiart1ev1aaatCvAUfKttLearuWrP9MDH5MBPbIqV92AaeXatLxBI9gBaebbnrfifHhDYfgasaacPC6xNi=xH8viVGI8Gi=hEeeu0xXdbba9frFj0xb9qqpG0dXdb9aspeI8k8fiI+fsY=rqGqVepae9pg0db9vqaiVgFr0xfr=xfr=xc9adbaqaaeGacaGaaiaabeqaaeqabiWaaaGcbaGaeeitaW0aa0baaSqaaiabikdaYaqaaiabiodaZaaaaaa@2F05@ + b C_1_

12. Quintic & curvature: a_0 _+ a_1 _L_2 _+ a_2 _L22
 MathType@MTEF@5@5@+=feaafiart1ev1aaatCvAUfKttLearuWrP9MDH5MBPbIqV92AaeXatLxBI9gBaebbnrfifHhDYfgasaacPC6xNi=xH8viVGI8Gi=hEeeu0xXdbba9frFj0xb9qqpG0dXdb9aspeI8k8fiI+fsY=rqGqVepae9pg0db9vqaiVgFr0xfr=xfr=xc9adbaqaaeGacaGaaiaabeqaaeqabiWaaaGcbaGaeeitaW0aa0baaSqaaiabikdaYaqaaiabikdaYaaaaaa@2F03@ + a_3 _L23
 MathType@MTEF@5@5@+=feaafiart1ev1aaatCvAUfKttLearuWrP9MDH5MBPbIqV92AaeXatLxBI9gBaebbnrfifHhDYfgasaacPC6xNi=xH8viVGI8Gi=hEeeu0xXdbba9frFj0xb9qqpG0dXdb9aspeI8k8fiI+fsY=rqGqVepae9pg0db9vqaiVgFr0xfr=xfr=xc9adbaqaaeGacaGaaiaabeqaaeqabiWaaaGcbaGaeeitaW0aa0baaSqaaiabikdaYaqaaiabiodaZaaaaaa@2F05@ + a_4 _L24
 MathType@MTEF@5@5@+=feaafiart1ev1aaatCvAUfKttLearuWrP9MDH5MBPbIqV92AaeXatLxBI9gBaebbnrfifHhDYfgasaacPC6xNi=xH8viVGI8Gi=hEeeu0xXdbba9frFj0xb9qqpG0dXdb9aspeI8k8fiI+fsY=rqGqVepae9pg0db9vqaiVgFr0xfr=xfr=xc9adbaqaaeGacaGaaiaabeqaaeqabiWaaaGcbaGaeeitaW0aa0baaSqaaiabikdaYaqaaiabisda0aaaaaa@2F07@ + a_5 _L25
 MathType@MTEF@5@5@+=feaafiart1ev1aaatCvAUfKttLearuWrP9MDH5MBPbIqV92AaeXatLxBI9gBaebbnrfifHhDYfgasaacPC6xNi=xH8viVGI8Gi=hEeeu0xXdbba9frFj0xb9qqpG0dXdb9aspeI8k8fiI+fsY=rqGqVepae9pg0db9vqaiVgFr0xfr=xfr=xc9adbaqaaeGacaGaaiaabeqaaeqabiWaaaGcbaGaeeitaW0aa0baaSqaaiabikdaYaqaaiabiwda1aaaaaa@2F09@ + b C_2_

13. Quintic & GC ratio: a_0 _+ a_1 _L_2 _+ a_2 _L22
 MathType@MTEF@5@5@+=feaafiart1ev1aaatCvAUfKttLearuWrP9MDH5MBPbIqV92AaeXatLxBI9gBaebbnrfifHhDYfgasaacPC6xNi=xH8viVGI8Gi=hEeeu0xXdbba9frFj0xb9qqpG0dXdb9aspeI8k8fiI+fsY=rqGqVepae9pg0db9vqaiVgFr0xfr=xfr=xc9adbaqaaeGacaGaaiaabeqaaeqabiWaaaGcbaGaeeitaW0aa0baaSqaaiabikdaYaqaaiabikdaYaaaaaa@2F03@ + a_3 _L23
 MathType@MTEF@5@5@+=feaafiart1ev1aaatCvAUfKttLearuWrP9MDH5MBPbIqV92AaeXatLxBI9gBaebbnrfifHhDYfgasaacPC6xNi=xH8viVGI8Gi=hEeeu0xXdbba9frFj0xb9qqpG0dXdb9aspeI8k8fiI+fsY=rqGqVepae9pg0db9vqaiVgFr0xfr=xfr=xc9adbaqaaeGacaGaaiaabeqaaeqabiWaaaGcbaGaeeitaW0aa0baaSqaaiabikdaYaqaaiabiodaZaaaaaa@2F05@ + a_4 _L24
 MathType@MTEF@5@5@+=feaafiart1ev1aaatCvAUfKttLearuWrP9MDH5MBPbIqV92AaeXatLxBI9gBaebbnrfifHhDYfgasaacPC6xNi=xH8viVGI8Gi=hEeeu0xXdbba9frFj0xb9qqpG0dXdb9aspeI8k8fiI+fsY=rqGqVepae9pg0db9vqaiVgFr0xfr=xfr=xc9adbaqaaeGacaGaaiaabeqaaeqabiWaaaGcbaGaeeitaW0aa0baaSqaaiabikdaYaqaaiabisda0aaaaaa@2F07@ + a_5 _L25
 MathType@MTEF@5@5@+=feaafiart1ev1aaatCvAUfKttLearuWrP9MDH5MBPbIqV92AaeXatLxBI9gBaebbnrfifHhDYfgasaacPC6xNi=xH8viVGI8Gi=hEeeu0xXdbba9frFj0xb9qqpG0dXdb9aspeI8k8fiI+fsY=rqGqVepae9pg0db9vqaiVgFr0xfr=xfr=xc9adbaqaaeGacaGaaiaabeqaaeqabiWaaaGcbaGaeeitaW0aa0baaSqaaiabikdaYaqaaiabiwda1aaaaaa@2F09@ + b GC_2_

14. Quintic & L_1_& GC ratio: a_0 _+ a_1 _L_2 _+ a_2 _L22
 MathType@MTEF@5@5@+=feaafiart1ev1aaatCvAUfKttLearuWrP9MDH5MBPbIqV92AaeXatLxBI9gBaebbnrfifHhDYfgasaacPC6xNi=xH8viVGI8Gi=hEeeu0xXdbba9frFj0xb9qqpG0dXdb9aspeI8k8fiI+fsY=rqGqVepae9pg0db9vqaiVgFr0xfr=xfr=xc9adbaqaaeGacaGaaiaabeqaaeqabiWaaaGcbaGaeeitaW0aa0baaSqaaiabikdaYaqaaiabikdaYaaaaaa@2F03@ + a_3 _L23
 MathType@MTEF@5@5@+=feaafiart1ev1aaatCvAUfKttLearuWrP9MDH5MBPbIqV92AaeXatLxBI9gBaebbnrfifHhDYfgasaacPC6xNi=xH8viVGI8Gi=hEeeu0xXdbba9frFj0xb9qqpG0dXdb9aspeI8k8fiI+fsY=rqGqVepae9pg0db9vqaiVgFr0xfr=xfr=xc9adbaqaaeGacaGaaiaabeqaaeqabiWaaaGcbaGaeeitaW0aa0baaSqaaiabikdaYaqaaiabiodaZaaaaaa@2F05@ + a_4 _L24
 MathType@MTEF@5@5@+=feaafiart1ev1aaatCvAUfKttLearuWrP9MDH5MBPbIqV92AaeXatLxBI9gBaebbnrfifHhDYfgasaacPC6xNi=xH8viVGI8Gi=hEeeu0xXdbba9frFj0xb9qqpG0dXdb9aspeI8k8fiI+fsY=rqGqVepae9pg0db9vqaiVgFr0xfr=xfr=xc9adbaqaaeGacaGaaiaabeqaaeqabiWaaaGcbaGaeeitaW0aa0baaSqaaiabikdaYaqaaiabisda0aaaaaa@2F07@ + a_5 _L25
 MathType@MTEF@5@5@+=feaafiart1ev1aaatCvAUfKttLearuWrP9MDH5MBPbIqV92AaeXatLxBI9gBaebbnrfifHhDYfgasaacPC6xNi=xH8viVGI8Gi=hEeeu0xXdbba9frFj0xb9qqpG0dXdb9aspeI8k8fiI+fsY=rqGqVepae9pg0db9vqaiVgFr0xfr=xfr=xc9adbaqaaeGacaGaaiaabeqaaeqabiWaaaGcbaGaeeitaW0aa0baaSqaaiabikdaYaqaaiabiwda1aaaaaa@2F09@ + b_0 _Log(L_1_) + b_1 _GC_1_

where L_2_, C_2 _and GC_2 _are the length, curvature and GC ratio of the second sequence in the pair and L_1 _is the length of the first sequence.

Formula coefficients were derived by regression analysis using the Matlab robustfit procedure applied to laboratory RLGS spots with known sequences. Matlab robustfit is a robust version of least squares. To avoid numerical instability, coefficients for the quintic polynomials were derived using Chebyshev polynomials The two sequences determining the horizontal and vertical migration of a laboratory spot are the two sequences associated with its matching virtual spot. Confirmed matches produced using vRLGS produced new spots with known sequences that were then used to improve the prediction formula. Thus, vRLGS was used to improve its own accuracy.

### Image generation

A 300-dpi virtual RLGS image is generated from the spot locations produced by fff2cnm.pl. Each spot is modeled as a Gaussian distribution, exp(-(dx)^2^/400-(dy)^2^/225), where dx and dy are the horizontal and vertical distance from the spot location. RLGS spots have greater width than height, and the parameters in the Gaussian distribution reflect this difference. Ribosomal DNA generates multiple fragments with the same DNA sequence. These fragments migrate to the same location on the gel, creating spots which are significantly larger and darker than others. Virtual spots from ribosomal DNA are modeled as the Gaussian distribution, exp(-(dx)^2^/4500-(dy)^2^/2500).

If two or more spots overlap, then their combined intensity at a pixel is computed as 1 - (1-I_1_) × (1-I_2_) × (1-I_3_) × ... where I_1_, I_2_, I_3_, ... is the intensity contribution from each of the spots at the given pixel. Intensities are measured from 0 to 1 with 0 as white and 1 as black.

### Conime

Conime is a software program developed at The Ohio State University for the comparison and analysis of RLGS gels [32]. It allows users to view, filter and register RLGS images, identify and match image spots and measure differences between spots. We used conime to compare laboratory and virtual RLGS gels.

### RLGS spot sequence confirmation

Spots identified through use of any of the three previously published arrayed boundary librarys (human NotI-EcoRV-HinfI [11], and AscI-EcoRV-HinfI [12] and mouse NotI-EcoRV-HinfI [13]) were confirmed for accuracy by the clone mixing gels approach previously described. Candidate sequences for spots as determined by vRLGS were confirmed using three different methods:

**A) **Candidate sequences were confirmed by designing PCR primers with the NotI-HinfI fragment of the RLGS spot. The spot of interest and a nearby negative control spot was eluted from the acrylamide gel as described [17] and these eluted spot DNAs were used as template for PCR. A candidate was deemed correct if a strong PCR product was obtained when DNA eluted from the spot of interest was used and either no band or a very weak band was detected when DNA eluted from the negative control spot was used as template. In cases where the spot of interest produced a strong band and the negative control spot produced a moderate intensity or greater band, such a candidate was deemed to be ambiguous and is not reported as the correct identify of the spot of interest. In cases where the spot of interest did not amplify, the candidate was deemed as incorrect.

**B) **Using the BLAT search tool we identified a BAC clone containing the full sequence of the candidate for the spot of interest. The BAC clone was then used in a typical RLGS mixing gel as previously described [3]. The candidate was deemed as correct if the spot of interest was enhanced in the BAC mixing gel.

**C) **We designed PCR primers outside the positions of the NotI site and EcoRV site so that the amplified product would contain the entirety of the 1^st ^dimension NotI- EcoRV fragment. Normal PBL genomic DNA (Promega) was used as template for PCR and the entire PCR product was cloned using the TOPO cloning kit (Invitrogen). The clone was then used in typical RLGS mixing gels as previously described [11] and the candidate was deemed correct if the spot of interest was enhanced on the PCR clone mixing gel.

### Genome database

We used UCSC Genome Bioinformatics site [33] as our source for the human and mouse fasta files containing DNA sequences. The human sequence is from the March 2006 release (hg18). The mouse sequence is from the February 2006 release (mm8) of the C57BL/6J mouse strain.

### RLGS gels

We tested vRLGS on three different laboratory gels: a human NotI-EcoRV-HinfI gel, a human AscI-EcoRV-HinfI gel and a mouse NotI-EcoRV-HinfI gel that serve as our "Master Profiles" as previously described [12,13,26]. For each of the three gels, we derived a separate set of formula coefficients predicting spot locations. Formulas for the human NotI-EcoRV-HinfI gel were derived from a set of 683 identified spots on that gel. Formulas for the human AscI-EcoRV-HinfI gel were derived from a set of 133 identified spots on the AscI-EcoRV-HinfI gel. Formulas for the mouse NotI-EcoRV-HinfI gel were derived from a set of 476 spots on the mouse gel.

## Authors' contributions

DJS, CP, and RW conceived of the work, participated in its design and coordination and contributed significantly to the drafting of the manuscript. RW, RKM, and TH developed the image software used. RW developed the PERL scripts associated with the migration algorithms. PL and JS created the methylation prediction software and generated the data for Additional files 5 and 6 II participated in the migration algorithm improvements based on sequence characteristics. DJS, CCO, YZW, LY, CL, SSC, SHW, DWD, JHS, MAT, and JMT participated in the cloning and confirmation of hundreds of mouse RLGS spots. DJS, LJR, LTS, JC, AS, MC, and CP participated in the cloning and confirmation of hundreds of human RLGS spots. WH, RE, and FS performed the comparison studies between vRLGS spot mapping of mouse spots and previous genetic mapping. All authors have read and approved the final version of the manuscript.

## Supplementary Material

Additional File 1 vRLGS for the human genome using the enzyme combination AscI-EcoRV-HinfI. Virtual profile for the human genome using the enzyme combination AscI-EcoRV-HinfI. Click here for file

Additional File 2 vRLGS for the mouse genome using the enzyme combination NotI-EcoRV-HinfI. Virtual profile for the mouse genome using the enzyme combination NotI-EcoRV-HinfI. Click here for file

Additional File 3 Correspondence of vRLGS spot prediction and genetic mapping in the BXD recombinant inbred mapping. Correspondence of vRLGS spot prediction and genetic mapping in the BXD recombinant inbred mapping. Click here for file

Additional File 4 Correspondence of vRLGS spot prediction and genetic mapping in the BSS interspecific backcross mapping. Correspondence of vRLGS spot prediction and genetic mapping in the BSS interspecific backcross mapping. Click here for file

Additional File 5 Human RLGS spot clones (hg18). Annotation of all cloned human RLGS spots from enzyme combination NotI-EcoRV-HinfI. Click here for file

Additional File 6 Mouse RLGS spot clones (mm8). Annotation of all cloned mouse RLGS spots from enzyme combination NotI-EcoRV-HinfI. Click here for file
