# Supplementary figures and images for: Restriction Landmark Genomic Scanning (RLGS) spot identification by second generation virtual RLGS in multiple genomes with multiple enzyme combinations
Source: BMC Genomics. 2007 Nov 30;8:446. doi: 10.1186/1471-2164-8-446 (PMC2235865; doi:10.1186/1471-2164-8-446)

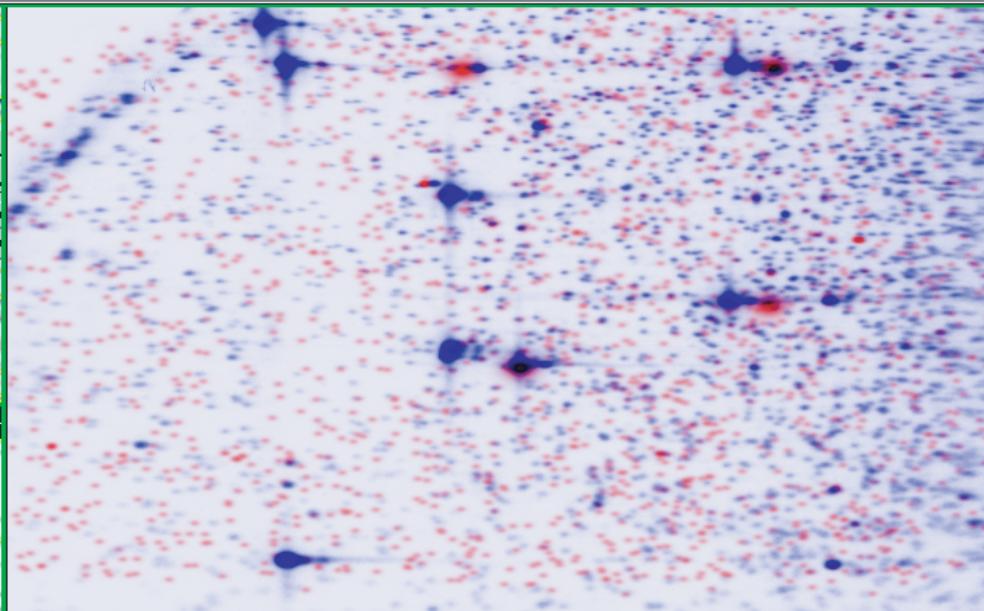

Human Ascl - EcoRV - HinfI actual gel

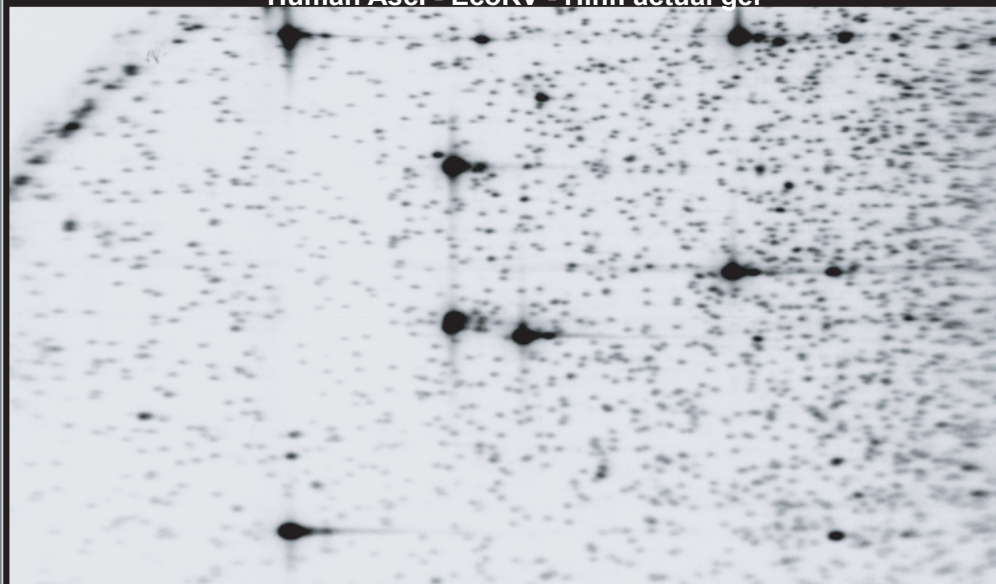

Human Ascl - EcoRV - HinfI virtual gel

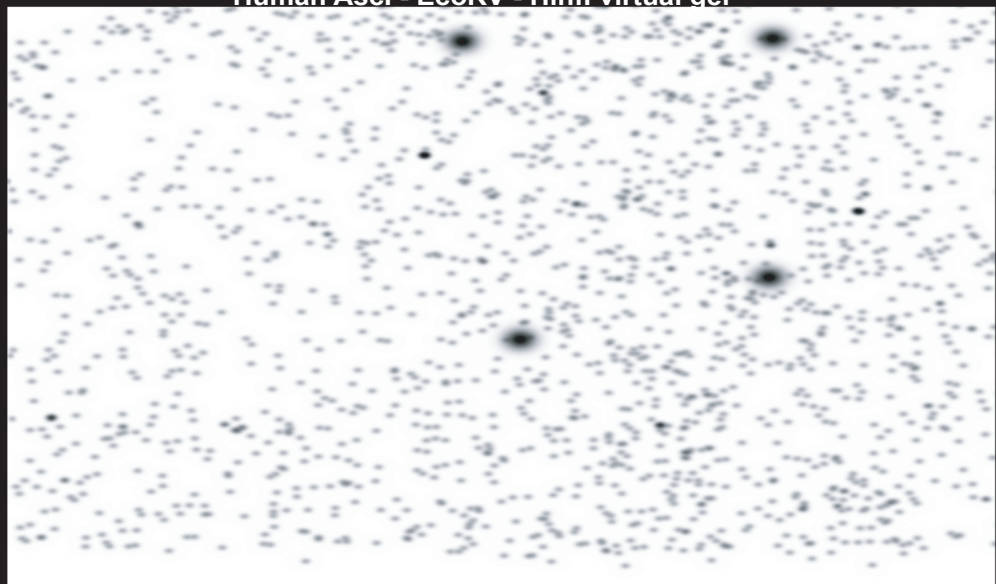

Supplement: Additional File 1 — vRLGS for the human genome using the enzyme combination AscI-EcoRV-HinfI. Virtual profile for the human genome using the enzyme combination AscI-EcoRV-HinfI. [file 1471-2164-8-446-S1.pdf]

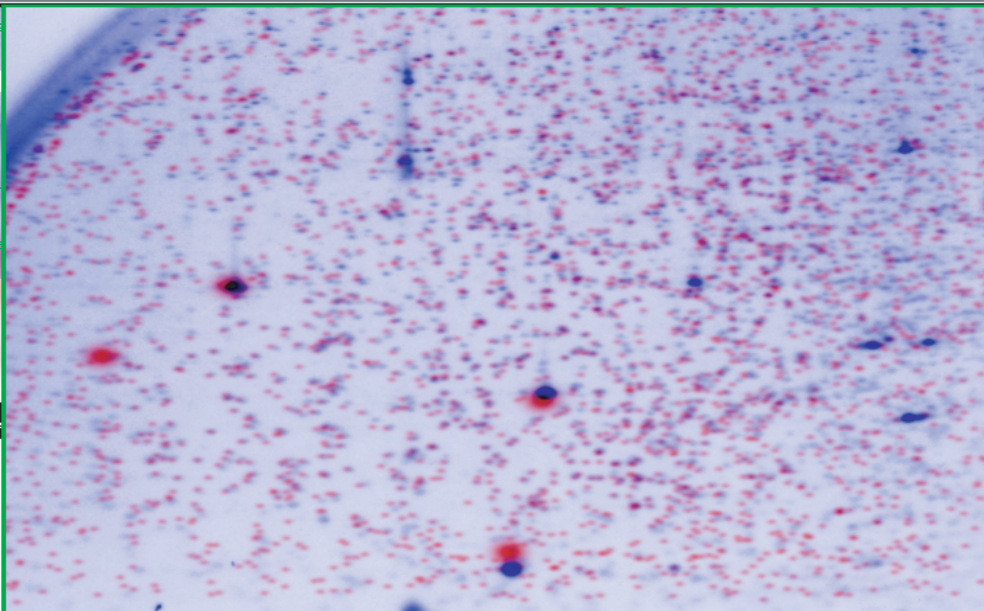

Mouse Not1 - EcoRV - HinfI actual gel

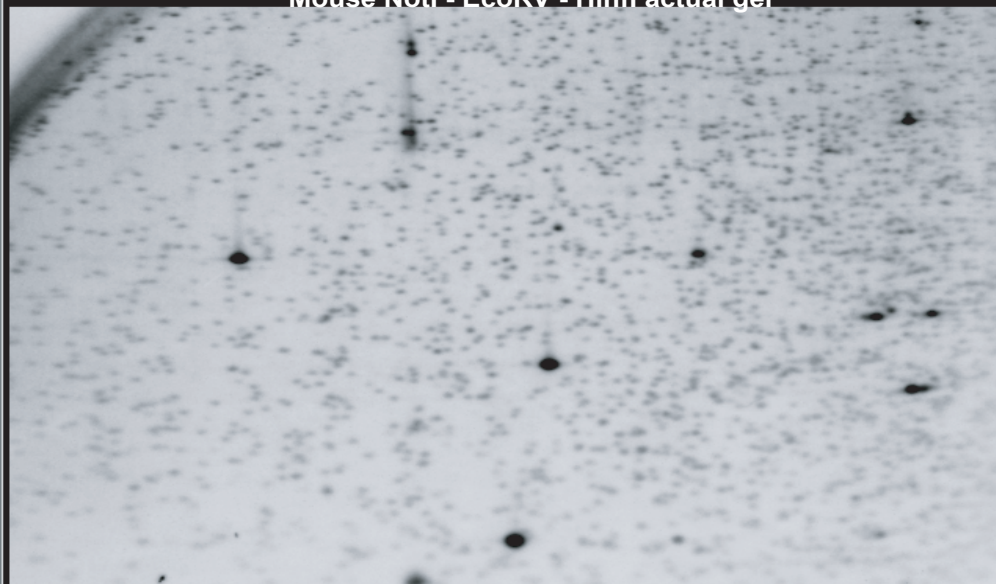

Mouse Not1 - EcoRV - HinfI virtual gel

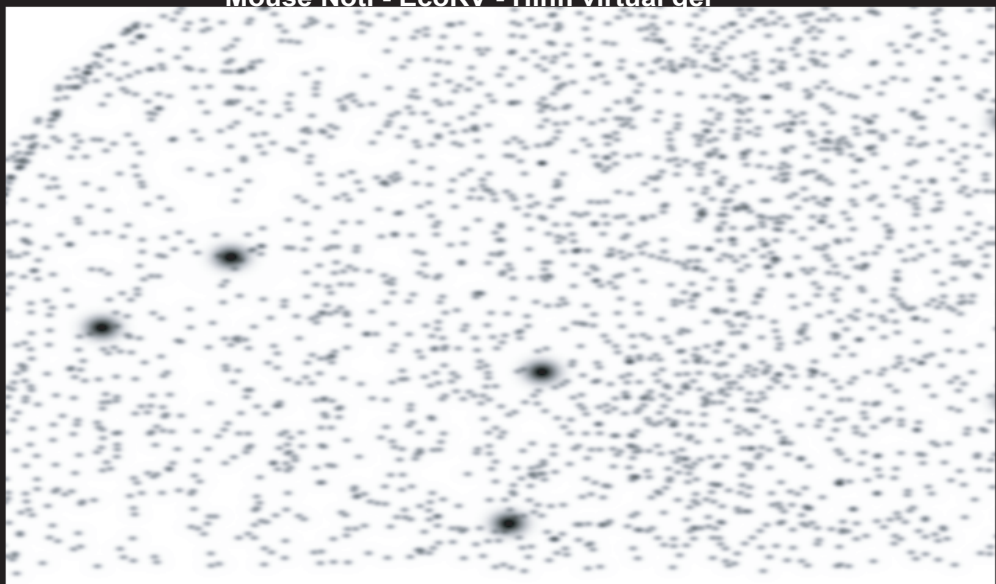

Supplement: Additional File 2 — vRLGS for the mouse genome using the enzyme combination NotI-EcoRV-HinfI. Virtual profile for the mouse genome using the enzyme combination NotI-EcoRV-HinfI. [file 1471-2164-8-446-S2.pdf]
