# Supplementary material for: Restriction Landmark Genomic Scanning (RLGS) spot identification by second generation virtual RLGS in multiple genomes with multiple enzyme combinations
Source: BMC Genomics. 2007 Nov 30;8:446. doi: 10.1186/1471-2164-8-446 (PMC2235865; doi:10.1186/1471-2164-8-446)
Supplement: Additional File 3 — Correspondence of vRLGS spot prediction and genetic mapping in the BXD recombinant inbred mapping. Correspondence of vRLGS spot prediction and genetic mapping in the BXD recombinant inbred mapping. [file 1471-2164-8-446-S3.doc]

Supplemental data Table 1: Correspondence of vRLGS spot prediction and genetic mapping in the BXD recombinant inbred mapping

| **BXD Locus** | **BXD Spot** | **1kb region of vRLGS locus**  **Feb 2006 Freeze (mm8)** |
| --- | --- | --- |
| D1Rik90 | B51 | Chr1:131001441-131002441 |
| D1Rik103 | B54 | Chr1:135173040-135174040 |
| D1Rik92 | B6 | Chr1:136026154-136027154 |
| D1Rik96 | B25 | Chr1:154758782-154759782 |
| D1Rik87 | B89 | Chr1:159193717-159194717 |
| D1Rik101 | B55 | Chr1:191690674-191691674 |
| D11Rik102 | B118 | Chr11:50557585-50558585 |
| D11Rik104 | B92 | Chr11:75759971-75760971 |
| D11Rik87 | B35 | Chr11:76575135-76576135 |
| D11Rik89 | B76 | Chr11:84588597-84589597 |
| D11Rik92 | B56 | Chr11:84589752-84590752 |
| D11Rik88 | B107 | Chr11:88168570-88169570 |
| D11Rik91 | B69 | Chr11:88168854-88169854 |
| D12Rik56 | B100 | Chr12:19229436-19230436 |
| D12Rik54 | B34 | Chr12:73693634-73694634 |
| D13Rik52 | B94 | Chr13:66444151-66445151 |
| D13Rik54 | B82 | Chr13:54038193-54039193 |
| D14Rik50 | B109 | Chr14:42509431-42510431 |
| D15Rik37 | B43 | Chr15:3822863-3823863 |
| D15Rik36 | B88 | Chr15:85005508-85006508 |
| D16Rik32 | B60 | Chr16:91305208-91306208 |
| D17Rik47 | B47 | Chr17:72092625-72093625 |
| D18Rik30 | B44 | Chr18:68192148-68193148 |
| D19Rik37 | B125 | Chr19:17094159-17095159 |
| D19Rik39 | B71 | Chr19:23977712-23978712 |
| D19Rik43 | B17 | Chr19:43509091-43510091 |
| D2Rik66 | B84 | Chr2:152410007-152411007 |
| D2Rik74 | B38 | Chr2:30422459-30423459 |
| D2Rik71 | B36 | Chr2:24770084-24771084 |
| D3Rik61 | B42 | Chr3:137675067-137676067 |
| D4Rik122 | B52 | Chr4:119707810-119708810 |
| D4Rik117 | B116 | Chr4:140746093-140747093 |
| D4Rik123 | B59 | Chr4:21934353-21935353 |
| D4Rik105 | B90 | Chr4:43412774-43413774 |
| D4Rik107 | B113 | Chr4:75852677-75853677 |
| D5Rik78 | B108 | Chr5:145738867-145739867 |
| D5Rik77 | B37 | Chr5:145739123-145740123 |
| D5Rik83 | B83 | Chr5:23947335-23948335 |
| D5Rik68 | B85 | Chr5:73612587-73613587 |
| D5Rik82 | B46 | Chr5:8404701-8405701 |
| D6Rik65 | B10 | Chr6:48560692-48561692 |
| D6Rik59 | B50 | Chr6:91198218-91199218 |
| D6Rik61 | B115 | Chr6:92793483-92794483 |
| D7Rik72 | B120 | Chr7:108343071-108344071 |
| D7Rik82 | B13 | Chr7:59688329-59689329 |
| D7Rik81 | B80 | Chr7:61228651-61229651 |
| D7Rik75 | B112 | Chr7:96205434-96206434 |
| D8Rik78 | B65 | Chr8:105656282-105657282 |
| D8Rik80 | B57 | Chr8:34352999-34353999 |
| D8Rik84 | B11 | Chr8:69935735-69936735 |
| D8Rik79 | B53 | Chr8:81900819-81901819 |
| D9Rik65 | B49 | Chr9:105955155-105956155 |
| D9Rik67 | B114 | Chr9:109572371-109573371 |
| D9Rik68 | B101 | Chr9:121557381-121558381 |
| D9Rik63 | B106 | Chr9:90983938-90984938 |
