# Supplementary material for: Restriction Landmark Genomic Scanning (RLGS) spot identification by second generation virtual RLGS in multiple genomes with multiple enzyme combinations
Source: BMC Genomics. 2007 Nov 30;8:446. doi: 10.1186/1471-2164-8-446 (PMC2235865; doi:10.1186/1471-2164-8-446)
Supplement: Additional File 4 — Correspondence of vRLGS spot prediction and genetic mapping in the BSS interspecific backcross mapping. Correspondence of vRLGS spot prediction and genetic mapping in the BSS interspecific backcross mapping. [file 1471-2164-8-446-S4.doc]

Supplemental data Table 2: Correspondence of vRLGS spot prediction and genetic mapping in the BSS interspecific backcross mapping

| **BSS Locus** | **BSS Spot** | **1kb region of vRLGS locus**  **Feb 2006 Freeze (mm8)** |
| --- | --- | --- |
| D1 Ncvs9 | B 26 | Chr1:136025927-136026927 |
| D1 Ncvs13 | B 315 | Chr1:154164683-154165683 |
| D1 Ncvs15 | B 321 | Chr1:163273087-163274087 |
| D1 Ncvs2 | B 297 | Chr1:39817612-39818612 |
| D1 Ncvs3 | B 91 | Chr1:52864666-52865666 |
| D10Ncvs10 | B 14 | Chr10:118847595-118848595 |
| D10Ncvs3 | B 275 | Chr10:41697632-41698632 |
| D10Ncvs4 | B 71 | Chr10:58840990-58841990 |
| D10Ncvs1 | B 57 | Chr10:7655462-7656462 |
| D10Ncvs7 | B 229 | Chr10:80074435-80075435 |
| D10Ncvs9 | B 351 | Chr10:99972997-99973997 |
| D11Ncvs18 | B 68 | Chr11:115967178-115968178 |
| (D11Ncvs85) | B 433 | Chr11:115968257-115969257 |
| D11Ncvs21 | B 85 | Chr11:120658041-120659041 |
| D11Ncvs4 | B 6 | Chr11:49668712-49669712 |
| D11Ncvs8 | B 261 | Chr11:70104597-70105597 |
| D11Ncvs13 | B 245 | Chr11:78245922-78246922 |
| D11Ncvs10 | B 298 | Chr11:79672433-79673433 |
| D11Ncvs15 | B 232 | Chr11:88117557-88118557 |
| D12Ncvs10 | B 302 | Chr12:101025561-101026561 |
| D12Ncvs11 | B 313 | Chr12:101025992-101026992 |
| D12Ncvs12 | B 148 | Chr12:109092190-109093190 |
| D12Ncvs7 | B 236 | Chr12:110452892-110453892 |
| D12Ncvs1 | B 12 | Chr12:17594799-17595799 |
| D12Ncvs2 | B 25 | Chr12:22936392-22937392 |
| D12Ncvs9 | B 429 | Chr12:98523692-98524692 |
| D13Ncvs11 | B 30 | Chr13:110776886-110777886 |
| D13Ncvs9 | B 152 | Chr13:92687218-92688218 |
| (D14Ncvs12) | B 156 | Chr14:112839118-112840118 |
| D14Ncvs3 | B 23 | Chr14:27150320-27151320 |
| D14Ncvs16 | B 166 | Chr14:40848186-40849186 |
| D14Ncvs15 | B 465 | Chr14:46258043-46259043 |
| D14Ncvs14 | B 493 | Chr14:51752398-51753398 |
| D14Ncvs6 | B 154 | Chr14:52693776-52694776 |
| D14Ncvs13 | B 491 | Chr14:65535197-65536197 |
| (D14Ncvs11) | B 159 | Chr14:98897308-98898308 |
| D15Ncvs2 | B 290 | Chr15:38410178-38411178 |
| D15Ncvs35 | B 88 | Chr15:99073364-99074364 |
| D17Ncvs2 | B 291 | Chr17:24058493-24059493 |
| D17Ncvs4 | B 252 | Chr17:26514142-26515142 |
| D17Ncvs5 | B 164 | Chr17:42858243-42859243 |
| D17Ncvs9 | B 13 | Chr17:69462691-69463691 |
| D18Ncvs3 | B 150 | Chr18:53854563-53855563 |
| D18Ncvs6 | B 135 | Chr18:68033078-68034078 |
| D19Ncvs4 | B 63 | Chr19:29347617-29348617 |
| D19Ncvs6 | B 95 | Chr19:44448361-44449361 |
| D2Ncvs12 | B 70 | Chr2:155122228-155123228 |
| D2Ncvs13 | B 149 | Chr2:155852040-155853040 |
| D2Ncvs20 | B 304 | Chr2:181539241-181540241 |
| D2Ncvs19 | B 42 | Chr2:182093066-182094066 |
| D2Ncvs2 | B 158 | Chr2:27016823-27017823 |
| D2Ncvs5 | B 301 | Chr2:30718532-30719532 |
| D2Ncvs4 | B 34 | Chr2:32988679-32989679 |
| D2Ncvs8 | B 28 | Chr2:71716782-71717782 |
| D2Ncvs9 | B 448 | Chr2:76249472-76250472 |
| D3Ncvs6 | B 36 | Chr3:108940918-108941918 |
| D3Ncvs9 | B 346 | Chr3:125775108-125776108 |
| D3Ncvs3 | B 163 | Chr3:84371931-84372931 |
| D4Ncvs8 | B 1 | Chr4:107995935-107996935 |
| D4Ncvs13 | B 80 | Chr4:120106403-120107403 |
| D4Ncvs15 | B 347 | Chr4:130006476-130007476 |
| D4Ncvs18 | B 244 | Chr4:133665488-133666488 |
| D4Ncvs16 | B 75 | Chr4:133929938-133930938 |
| D4Ncvs1 | B 303 | Chr4:25035603-25036603 |
| D4Ncvs2 | B 157 | Chr4:26416154-26417154 |
| D4Ncvs5 | B 33 | Chr4:88125366-88126366 |
| D5Ncvs12 | B 238 | Chr5:103099531-103100531 |
| D5Ncvs15 | B 292 | Chr5:116349583-116350583 |
| (D5Ncvs17) | B 8 | Chr5:122929655-122930655 |
| D5Ncvs19 | B 323 | Chr5:134823096-134824096 |
| D5Ncvs22 | B 43 | Chr5:142559773-142560773 |
| D5Ncvs4 | B 18 | Chr5:29654718-29655718 |
| D5Ncvs8 | B 407 | Chr5:33431427-33432427 |
| D5Ncvs2 | B 255 | Chr5:3549220-3550220 |
| D5Ncvs9 | B 440 | Chr5:37884011-37885011 |
| D6Ncvs7 | B 318 | Chr6:113355314-113356314 |
| D6Ncvs2 | B 145 | Chr6:34711397-34712397 |
| D6Ncvs3 | B 257 | Chr6:52138316-52139316 |
| D7Ncvs17 | B 437 | Chr7:107938194-107939194 |
| D7Ncvs18 | B 134 | Chr7:122583422-122584422 |
| D7Ncvs19 | B 342 | Chr7:126430943-126431943 |
| D7Ncvs23 | B 398 | Chr7:138522129-138523129 |
| D7Ncvs3 | B 394 | Chr7:14035014-14036014 |
| D7Ncvs4 | B 436 | Chr7:24593276-24594276 |
| D7Ncvs10 | B 73 | Chr7:45998617-45999617 |
| D7Ncvs11 | B 350 | Chr7:61967238-61968238 |
| D7Ncvs14 | B 360 | Chr7:77701328-77702328 |
| D7Ncvs13 | B 331 | Chr7:77702342-77703342 |
| D8Ncvs9 | B 294 | Chr8:102995758-102996758 |
| D8Ncvs11 | B 59 | Chr8:105535404-105536404 |
| D8Ncvs13 | B 131 | Chr8:105656044-105657044 |
| D8Ncvs14 | B 96 | Chr8:115344419-115345419 |
| D8Ncvs17 | B 414 | Chr8:122240570-122241570 |
| D8Ncvs18 | B 434 | Chr8:122817871-122818871 |
| D8Ncvs16 | B 17 | Chr8:124885767-124886767 |
| D8Ncvs2 | B 258 | Chr8:34260341-34261341 |
| D8Ncvs3 | B 487 | Chr8:56035001-56036001 |
| D8Ncvs4 | B 72 | Chr8:69360578-69361578 |
| D8Ncvs7 | B 94 | Chr8:86792087-86793087 |
| (D9Ncvs14) | B 24 | Chr9:102915324-102916324 |
| D9Ncvs16 | B 343 | Chr9:106182880-106183880 |
| D9Ncvs2 | B 50 | Chr9:52146517-52147517 |
| D9Ncvs6 | B 430 | Chr9:66622278-66623278 |
| D9Ncvs5 | B 338 | Chr9:66976599-66977599 |
| D9Ncvs10 | B 276 | Chr9:71249776-71250776 |
| D9Ncvs12 | B 443 | Chr9:88427798-88428798 |
| D1Ncvs11 | B 408 | Chr1:152727028-152728028 |
| D1 Ncvsl4 | B 379 | Chr1:155059455-155060455 |
| D11Ncvs9 | B 418 | Chr11:74804446-74805446 |
| D12Ncvs4 | B 386 | Chr12:22516761-22517761 |
| D19Ncvs1 | B 391 | Chr19:14492049-14493049 |
| D4Ncvs22 | B 385 | Chr4:151167686-151168686 |
| D5Ncvs7 | B 406 | Chr5:33431427-33432427 |
| D7Ncvs22 | B 405 | Chr7:130113796-130114796 |
| D8Ncvs5 | B 380 | Chr8:70841815-70842815 |
| D10Ncvs6 | B 47 | Chr10:81243740-81244740 |
| D11Ncvs17 | B 170 | Chr11:96516445-96517445 |
| D16Ncvs2 | B 259 | Chr16:16783389-16784389 |
| D2Ncvs14 | B 3 | Chr2:167020973-167021973 |
| D7Ncvs9 | B 239 | Chr7:45859781-45860781 |
| D9Ncvs13 | B 27 | Chr9_random:301651-302651 |
| DXNcvs2 | B 447 | ChrX:130460896-130461896 |
