# Supplementary material for: Restriction Landmark Genomic Scanning (RLGS) spot identification by second generation virtual RLGS in multiple genomes with multiple enzyme combinations
Source: BMC Genomics. 2007 Nov 30;8:446. doi: 10.1186/1471-2164-8-446 (PMC2235865; doi:10.1186/1471-2164-8-446)
Supplement: Additional File 5 — Human RLGS spot clones (hg18). Annotation of all cloned human RLGS spots from enzyme combination NotI-EcoRV-HinfI. [file 1471-2164-8-446-S5.doc]

Supplementary data Table 3 Human RLGS spot clones (hg18)

| **Spot** | **NotI +/- 200bp (March 2006)** | **NotI site (March 2006)** | **%GC** | **O:E** | **CpG island** | **Cytogeneitc mapping** | **Gene Homology** | **Context** |
| --- | --- | --- | --- | --- | --- | --- | --- | --- |
| 1B01 | chr15:54072563-54072963 | 54072763 | 72 | 0.87 | Y | 15q21.3 | NEDD4 | Body |
| 1B02 | chr12:75983533-75983933 | 75983733 | 72 | 0.91 | Y | 12q21.2 | E2F7 | 5' end |
| 1C01 | chr18:46061644-46062044 | 46061844 | 68 | 0.90 | Y | 18q21.1 | MBD1 | 5' end |
| 1C03_D13 | chr9:99785109-99785509 | 99785309 | 72 | 0.96 | Y | 9q22.33 | ANP32B | 5' end |
| 1C04 | chr22:49048356-49048756 | 49048556 | 72 | 0.86 | Y | 22q13.33 | MAPK11 | Body |
| 1C05_06 | chr16:78190586-78190986 | 78190786 | 74 | 0.94 | Y | 16q23.1 | MAF | Body |
| 1C07 | chr17:39651517-39651917 | 39651717 | 78 | 0.79 | Y | 17q21.31 | UBTF | 5' end |
| 1C08 | chr2:64734509-64734909 | 64734709 | 76 | 0.90 | Y | 2p14 | TRIP-Br2 | 5' end |
| 1C09 | chr7:138371051-138371451 | 138371251 | 75 | 0.84 | Y | 7q34 | MGC14289 | 5' end |
| 1C12 | chr11:63338002-63338402 | 63338202 | 67 | 0.70 | Y | 11q13.1 | LOC144097 | Body |
| 1D04 | chr4:141638421-141638821 | 141638621 | 69 | 0.91 | Y | 4q31.1 | AK128168 | 5' end |
| 1D05 | chr9:94986853-94987253 | 94987053 | 77 | 0.84 | Y | 9q22.31 | PRKWNK2 | 5' end |
| 1D06 | chr16:47873760-47874160 | 47873960 | 72 | 1.00 | Y | 16q12.1 | CBLN1 | 5' end |
| 1D07 | chr11:33354196-33354596 | 33354396 | 78 | 0.96 | Y | 11p13 | NA | Intergenic |
| 1D09 | chr14:74813360-74813760 | 74813560 | 75 | 0.84 | Y | 14q24.3 | FOS | 5' end |
| 1D10 | chr6:12119808-12120208 | 12120008 | 83 | 0.85 | Y | 6p24.1 | HIVEP1 | 5' end |
| 1D11 | chr8:22354613-22355013 | 22354813 | 75 | 0.87 | Y | 8p21.3 | PPP3CC | 5' end |
| 1D14 | chr16:9091883-9092283 | 9092083 | 75 | 0.87 | Y | 16p13.2 | PRO0149 | 5' end |
| 1D16 | chr20:45563682-45564082 | 45563882 | 61 | 0.88 | Y | 20q13.12 | NCOA3 | 5' end |
| 1D17 | chr13:94417790-94418190 | 94417990 | 67 | 0.85 | Y | 13q32.1 | kleydarby | 5' end |
| 1D19 | chr13:49596239-49596639 | 49596439 | 74 | 0.97 | Y | 13q14.2 | DLEU2 | 5' end |
| 1D21 | chr17:20886811-20887211 | 20887011 | 74 | 0.88 | Y | 17p11.2 | USP22 | 5' end |
| 1E02 | chr21:29367465-29367865 | 29367665 | 63 | 0.85 | Y | 21q21.3 | CCT8 | Body |
| 1E05 | chr22:22529771-22530171 | 22529971 | 66 | 1.00 | Y | 22q11.23 | SLC2A11 | Body |
| 1E08 | chr12:108634230-108634630 | 108634430 | 76 | 0.95 | Y | 12q24.11 | BC020193 | 5' end |
| 1E09 | chr13:99418391-99418791 | 99418591 | 71 | 0.84 | Y | 13q32.3 | ZIC5 | Body |
| 1E10 | chr15:75711497-75711897 | 75711697 | 71 | 0.74 | Y | 15q24.3 | LRRN6A | Body |
| 1E11 | chr19:34709075-34709475 | 34709275 | 62 | 0.93 | Y | 19q12 | AK094793 | 5' end |
| 1E13 | chr7:107430734-107431134 | 107430934 | 69 | 0.88 | Y | 7q31.1 | LAMB1 | 5' end |
| 1E14 | chr19:11454391-11454791 | 11454591 | 70 | 0.76 | Y | 19p13.2 | ELAVL3 | 5' end |
| 1E18 | chr11:74740068-74740468 | 74740268 | 77 | 0.89 | Y | 11q13.4 | ARRB1 | Body |
| 1F01 | chr6:97192594-97192994 | 97192794 | low | low | N | 6q16.1 | AK055697 | Body |
| 1F03 | chr6:125664336-125664736 | 125664536 | 72 | 0.87 | Y | 6q22.31 | C6orf74 | Body |
| 1F07 | chr9:91115158-91115558 | 91115358 | 69 | 0.85 | Y | 9q22.2 | CKS2 | 5' end |
| 1F09 | chr13:46268646-46269046 | 46268846 | 57 | 0.90 | Y | 13q14.2 | ESD | 5' end |
| 1F10 | chr4:37569222-37569622 | 37569422 | 74 | 0.83 | Y | 4p14 | TBC1D1 | 5' end |
| 1F14 | chr20:43469141-43469541 | 43469341 | 66 | 0.53 | N | 20q13.12 | C20orf35 | Body |
| 1F15 | chr1:45249218-45249618 | 45249418 | 75 | 0.87 | Y | 1p34.1 | FLJ21156 | 5' end |
| 1F22 | chr9:125817263-125817663 | 125817463 | 66 | 0.98 | Y | 9q33.3 | LHX2 | Body |
| 1F30 | chr4:41579033-41579433 | 41579233 | 57 | 0.98 | Y | 4p13 | BC025350 | 5' end |
| 1G20 | chr3:182912605-182913005 | 182912805 | 69 | 0.97 | Y | 3q26.33 | SOX2 | 5' end |
| 1G30 | chr17:7248711-7249111 | 7248911 | 76 | 0.82 | Y | 17p13.1 | MGC40107 | 5' end |
| 2A01 | chr4:170427820-170428220 | 170428020 | 64 | 0.77 | Y | 4q33 | SH3MD2 | 5' end |
| 2A02 | chr10:116687942-116688342 | 116688142 | 62 | 0.77 | Y | 10q25.3 | TRUB1 | 5' end |
| 2B02 | chr11:124175294-124175694 | 124175494 | 73 | 0.93 | Y | 11q24.2 | FLJ23342 | 5' end |
| 2B03 | chr12:74763830-74764230 | 74764030 | 63 | 0.69 | Y | 12q21.2 | NAP1L1 | 5' end |
| 2B04 | chr13:49264010-49264410 | 49264210 | 75 | 0.88 | Y | 13q14.2 | KPNA3 | Body |
| 2B08 | chr2:102338361-102338761 | 102338561 | 66 | 0.80 | Y | 2q11.2 | IL18R1 | 5' end |
| 2B09 | chr12:109046307-109046707 | 109046507 | 72 | 1.02 | Y | 12q24.11 | CDV-1 | 5' end |
| 2B10 | chr10:13668827-13669227 | 13669027 | 61 | 0.73 | Y | 10p13 | PRPF18 | 5' end |
| 2B20 | chr6:34219964-34220364 | 34220164 | 73 | 0.83 | Y | 6p21.31 | GRM4 | Body |
| 2B23 | chr7:151763664-151764064 | 151763864 | 74 | 0.88 | Y | 7q36.1 | MLL3 | 5' end |
| 2B24 | chr16:85100832-85101232 | 85101032 | 65 | 0.82 | Y | 16q24.1 | FOXF1 | 5' end |
| 2B25 | chr17:22645213-22645613 | 22645413 | low | low | N | 17q11.1 | WSB1 | 5' end |
| 2B30 | chr17:72218333-72218733 | 72218533 | 77 | 0.95 | Y | 2q24.2 | TMAP1 | 5' end |
| 2B33 | chr5:151130624-151131024 | 151130824 | 64 | 0.92 | Y | 5q33.1 | G3BP | 5' end |
| 2B36 | chr12:114374035-114374435 | 114374235 | 80 | 0.94 | Y | 12q24.21 | NA | Intergenic |
| 2B38 | chr20:1894331-1894731 | 1894531 | 58 | 0.74 | Y | 20p13 | AK090681 | Body |
| 2B46 | chr15:29406195-29406595 | 29406395 | 78 | 1.00 | Y | 15q13.3 | KLF13 | 5' end |
| 2B47 | chr2:106048354-106048754 | 106048554 | 77 | 0.79 | Y | 2q12.2 | ECRG4 | 5' end |
| 2B48 | chr8:144399683-144400083 | 144399883 | 61 | 0.98 | Y | 8q24.3 | FLJ38705 | 5' end |
| 2B51 | chr5:73972031-73972431 | 73972231 | 72 | 1.01 | Y | 5q13.3 | ENC1 | 5' end |
| 2B53 | chr22:17658760-17659160 | 17658960 | 75 | 0.75 | Y | 22q11.21 | CLTCL1 | Body |
| 2B54 | chr10:131659907-131660307 | 131660107 | 64 | 1.27 | Y | 10q26.3 | DKFZp667B0210 | 5' end |
| 2B56 | chr7:107954105-107954505 | 107954305 | 55 | 0.66 | Y | 7q31.1 | IPLA2(GAMMA) | 5' end |
| 2B57 | chr14:103058729-103059129 | 103058929 | 78 | 1.02 | Y | 14q32.32 | CKB | 5' end |
| 2B60 | chr17:59280310-59280710 | 59280510 | 68 | 1.07 | Y | 17q23.3 | sposa | 5' end |
| 2C01 | chr5:76541795-76542195 | 76541995 | 79 | 0.92 | Y | 5q13.3 | PDE8B | 5' end |
| 2C10 | chr15:66896919-66897319 | 66897119 | 67 | 0.89 | Y | 15q23 | ANP32A | Body |
| 2C12 | chr2:186311808-186312208 | 186312008 | 71 | 0.77 | Y | 2q32.1 | BC039382 | 5' end |
| 2C13 | chr8:102032938-102033338 | 102033138 | 63 | 0.84 | Y | 8q22.3 | YWHAZ | 5' end |
| 2C19 | chr11:17712786-17713186 | 17712986 | 77 | 0.90 | Y | 11p15.1 | KCNC1 | 5' end |
| 2C23 | chr1:234511359-234511759 | 234511559 | 70 | 0.88 | Y | 1q42.3 | ERO1LB | Body |
| 2C24 | chr3:9570143-9570543 | 9570343 | 78 | 0.88 | Y | 3p25.3 | LOC375323 | 5' end |
| 2C25 | chr10:126127237-126127637 | 126127437 | 73 | 0.89 | Y | 10q26.13 | NA | Intergenic |
| 2C29 | chr6:100075462-100075862 | 100075662 | 58 | 0.79 | Y | 6q16.2 | BX647075 | 5' end |
| 2C30 | chr1:199108900-199109300 | 199109100 | 71 | 0.84 | Y | 1q32.1 | GPR25 | 5' end |
| 2C32 | chr10:121475922-121476322 | 121476122 | 68 | 0.74 | Y | 10q26.11 | INPP5F | Body |
| 2C33 | chr7:2850142-2850542 | 2850342 | 83 | 1.11 | Y | 7p22.2 | GNA12 | 5' end |
| 2C34 | chr12:102982498-102982898 | 102982698 | 74 | 0.88 | Y | 12q23.3 | HCFC2 | Body |
| 2C35 | chr10:23502265-23502665 | 23502465 | 65 | 1.11 | Y | 10p12.2 | NA | Intergenic |
| 2C37 | chr9:72223719-72224119 | 72223919 | 64 | 0.94 | Y | 9q21.11 | KLF9 | 5' end |
| 2C39 | chr16:12903167-12903567 | 12903367 | 79 | 1.04 | Y | 16p13.12 |  | 5' end |
| 2C40 | chr11:627333-627733 | 627533 | 74 | 0.90 | Y | 11p15.5 | DRD4 | 5' end |
| 2C42 | chr18:18002204-18002604 | 18002404 | 55 | 0.88 | Y | 18q11.2 | GATA6 | 5' end |
| 2C44 | chr14:24589021-24589421 | 24589221 | 75 | 0.87 | Y | 14q12 | STXBP6 | 5' end |
| 2c46 | chr1:26895706-26896106 | 26895906 | 61 | 1.00 | Y | 1p36.11 | ARID1A | 5' end |
| 2C47 | chr21:44351491-44351891 | 44351691 | 75 | 1.13 | Y | 21q22.3 | PWP2H | 5' end |
| 2C49 | chr12:67613026-67613426 | 67613226 | 78 | 0.89 | Y | 12q15 | CPM | 5' end |
| 2C51 | chr17:12861693-12862093 | 12861893 | 71 | 1.14 | Y | 17p12 | ELAC2 | 5' end |
| 2C53 | chr2:238264560-238264960 | 238264760 | 71 | 0.86 | Y | 2q37.3 | LRRFIP1 | 5' end |
| 2C54 | chr2:225158261-225158661 | 225158461 | 72 | 0.85 | Y | 2q36.2 | CUL3 | 5' end |
| 2C57 | chr10:103579943-103580343 | 103580143 | 62 | 0.64 | Y | 10q24.32 | KCNIP2 | Body |
| 2C58 | chr18:30058291-30058691 | 30058491 | 60 | 0.83 | Y | 18q12.1 | NOL4 | 5' end |
| 2C59 | chr19:42776897-42777297 | 42777097 | 58 | 0.69 | Y | 19q13.13 | ZNF540 | 5' end |
| 2C60 | chr2:64831286-64831686 | 64831486 | 64 | 0.69 | Y | 2p14 | AK098596 | 5' end |
| 2C62 | chr3:47595080-47595480 | 47595280 | 82 | 1.06 | Y | 3p21.31 | CSPG5 | 5' end |
| 2C64 | chr10:47124281-47124681 | 47124481 | 64 | 0.67 | Y | 10q11.22 | BC031882 | 5' end |
| 2C65L | chr15:26832741-26833141 | 26832941 | 62 | 0.88 | Y | 15q13.1 | slopor | 5' end |
| 2C65R | chr7:43764781-43765181 | 43764981 | 70 | 1.00 | Y | 7p13 | BLVRA | 5' end |
| 2C66 | chr14:55654263-55654663 | 55654463 | 57 | 0.81 | Y | 14q22.3 | PELI2 | 5' end |
| 2D09 | chr1:232416751-232417151 | 232416951 | 73 | 0.71 | Y | 1q42.2 | SLC35F3 | Body |
| 2D10 | chr5:979471-979871 | 979671 | 54 | 0.95 | Y | 5p15.33 | NA | Intergenic |
| 2D12 | chr10:111757756-111758156 | 111757956 | 68 | 0.90 | Y | 10q25.1 | ADD3 | 5' end |
| 2D13 | chr16:79626671-79627071 | 79626871 | 68 | 0.98 | Y | 16q23.2 | KIAA0431 | 5' end |
| 2D14 | chr2:86942297-86942697 | 86942497 | 76 | 0.74 | Y | 2p11.2 | CD8B1 | 5' end |
| 2D15 | chr1:27025927-27026327 | 27026127 | 76 | 0.95 | Y | 1p36.11 | ZDHHC18 | 5' end |
| 2D16 | chr5:72768218-72768618 | 72768418 | 76 | 0.88 | Y | 5q13.2 | NA | Intergenic |
| 2D17 | chr6:3402268-3402668 | 3402468 | 78 | 0.84 | Y | 6p25.2 | C6orf85 | 5' end |
| 2D20 | chr2:63136177-63136577 | 63136377 | 67 | 0.86 | Y | 2p15 | OTX1 | Body |
| 2D21_22 | chr1:234096559-234096959 | 234096759 | 75 | 0.90 | Y | 1q42.3 | LYST | 5' end |
| 2D24 | chr8:21979901-21980301 | 21980101 | 73 | 0.92 | Y | 8p21.3 | EPB49 | 5' end |
| 2D25 | chr10:135086929-135087329 | 135087129 | 61 | 0.65 | Y | 10q26.3 | Sprn | 5' end |
| 2D27 | chr4:174326824-174327224 | 174327024 | 80 | 0.89 | Y | 4q34.1 | GALNT7 | Body |
| 2D28 | chr15:29950029-29950429 | 29950229 | 78 | 1.19 | Y | 15q13.3 | AK126002 | 5' end |
| 2D30 | chr8:22044338-22044738 | 22044538 | 74 | 0.77 | Y | 8p21.3 | HR | 5' end |
| 2D31 | chr7:65084204-65084604 | 65084404 | 74 | 0.81 | Y | 7q11.21 | GUSB | 5' end |
| 2D33 | chr16:45476014-45476414 | 45476214 | 71 | 0.96 | Y | 16q11.2 | GPT2 | Body |
| 2D34 | chr8:81947988-81948388 | 81948188 | 75 | 0.87 | Y | 8q21.13 |  | 5' end |
| 2D38 | chr20:60131055-60131455 | 60131255 | 76 | 0.95 | Y | 20q13.33 | C20orf40 | 5' end |
| 2D40 | chr6:44203086-44203486 | 44203286 | 75 | 0.90 | Y | 6p21.1 | MRPL14 | 5' end |
| 2D45 | chr19:36534086-36534486 | 36534286 | 72 | 0.95 | Y | 19q12 | ZNF537 | 5' end |
| 2D46 | chr5:170669561-170669961 | 170669761 | 68 | 0.98 | Y | 5q35.1 | TLX3 | Body |
| 2D48 | chr7:27231338-27231738 | 27231538 | 58 | 0.64 | Y | 7p15.2 | NA | Intergenic |
| 2D50 | chr22:41245369-41245769 | 41245569 | 73 | 0.73 | Y | 22q13.2 | CGI-96 | Body |
| 2D51 | chr8:32525478-32525878 | 32525678 | 72 | 1.07 | Y | 8p12 | NRG1 | 5' end |
| 2D53 | chr16:4105987-4106387 | 4106187 | 85 | 1.07 | Y | 16p13.3 | ADCY9 | 5' end |
| 2D55 | chr22:42589591-42589991 | 42589791 | 78 | 1.25 | Y | 22q13.31 | SULT4A1 | 5' end |
| 2D56 | chr17:28227690-28228090 | 28227890 | 75 | 0.88 | Y | 17q11.2 | MYO1D | Body |
| 2D57 | chr22:15896927-15897327 | 15897127 | 63 | 0.67 | Y | 22q11.1 | LOC391290 | 5' end |
| 2D61 | chr17:582109-582509 | 582309 | 68 | 1.03 | Y | 17p13.3 | FAM57A | 5' end |
| 2D65 | chr19:39916665-39917065 | 39916865 | 61 | 0.85 | Y | 19q13.12 | ZNF181 | Body |
| 2D66 | chr17:52030015-52030415 | 52030215 | 61 | 0.78 | Y | 17q23.2 | NA | Intergenic |
| 2D67 | chr15:64921687-64922087 | 64921887 | 72 | 0.82 | Y | 15q22.31 | CCAR1 | 5' end |
| 2D68 | chr10:104394156-104394556 | 104394356 | 72 | 0.83 | Y | 10q24.32 | TRIM8 | 5' end |
| 2D69 | chr17:58877004-58877404 | 58877204 | 75 | 0.78 | Y | 17q23.3 | CYB561 | 5' end |
| 2D70 | chr1:218334015-218334415 | 218334215 | 72 | 0.77 | Y | 1q41 | FLJ10326 | 5' end |
| 2D71 | chr16:84390324-84390724 | 84390524 | 72 | 0.95 | Y | 16q24.1 | COX4I1; NOC4 | 5' end |
| 2D72 | chr4:99798548-99798948 | 99798748 | 79 | 1.17 | Y | 4q23 | TM4SF9 | 5' end |
| 2D74 | chr3:26639210-26639610 | 26639410 | 70 | 0.94 | Y | 3p24.2 | LRP15 | 5' end |
| 2E01 | chr12:102982505-102982905 | 102982705 | 74 | 0.88 | Y | 12q23.3 | HCFC2 | Body |
| 2E02 | chr9:114289106-114289506 | 114289306 | 78 | 0.95 | Y | 9q32 | AK131020 | 5' end |
| 2E03 | chr3:43707661-43708061 | 43707861 | 73 | 0.94 | Y | 3p21.33 | ABHD5 | Body |
| 2E06 | chr12:95318095-95318495 | 95318295 | 71 | 1.17 | Y | 12q23.1 | PCTK2 | 5' end |
| 2E09 | chr2:233058586-233058986 | 233058786 | 70 | 0.96 | Y | 2q37.1 | ECEL1 | Body |
| 2E10 | chr9:98456875-98457275 | 98457075 | 72 | 1.04 | Y | 9q22.32 | C9orf21.eNov04 | 5' end |
| 2E12 | chr21:44544786-44545186 | 44544986 | 73 | 0.63 | Y | 21q22.3 | PFKL | 5' end |
| 2E14 | chr11:64640979-64641379 | 64641179 | 70 | 1.02 | Y | 11q13.1 | C11orf5 | 5' end |
| 2E15 | chr19:52055532-52055932 | 52055732 | 71 | 1.12 | Y | 19q13.32 | SAE1 | Body |
| 2E16 | chr2:98427818-98428218 | 98428018 | 78 | 0.92 | Y | 2q11.2 | INPP4A | 5' end |
| 2E17 | chr17:52026137-52026537 | 52026337 | 74 | 0.93 | Y | 17q23.2 | NOG | 5' end |
| 2E19 | chr14:61348875-61349275 | 61349075 | 78 | 1.03 | Y | 14q23.2 | spacheybu | 5' end |
| 2E20 | chr10:26542837-26543237 | 26543037 | low | low | N | 10p12.1 | GAD2 | 5' end |
| 2E21 | chr20:21054906-21055306 | 21055106 | 60 | 0.59 | N | 20p11.23 | C20orf19 | Body |
| 2E22 | chr1:11673687-11674087 | 11673887 | 70 | 0.88 | Y | 1p36.22 | LOC374946 | 5' end |
| 2E23 | chr4:48037991-48038391 | 48038191 | 77 | 0.83 | Y | 4p12 | BC040993 | 5' end |
| 2E24 | chr22:36806839-36807239 | 36807039 | 71 | 1.06 | Y | 22q13.1 | SLC16A8 | Body |
| 2E25 | chr19:18515158-18515558 | 18515358 | 79 | 0.79 | Y | 19p13.11 | FKBP8 | 5' end |
| 2E26 | chr6:168244826-168245226 | 168245026 | 76 | 0.83 | Y | 6q27 | dyflarby | 5' end |
| 2E27 | chr3:114412986-114413386 | 114413186 | 66 | 0.94 | Y | 3q13.2 | BOC | 5' end |
| 2E28 | chr13:27261732-27262132 | 27261932 | 70 | 0.69 | Y | 13q12.2 | GSH1 | 5' end |
| 2E30 | chr18:43027591-43027991 | 43027791 | 79 | 0.90 | Y | 18q21.1 | AF119875 | Body |
| 2E33 | chr1:110412400-110412800 | 110412600 | 57 | 0.90 | Y | 1p13.3 | ALX3 | Body |
| 2E34 | chr4:6627576-6627976 | 6627776 | 73 | 0.68 | Y | 4p16.1 | KIAA0935 | 5' end |
| 2E35 | chr1:50659488-50659888 | 50659688 | 71 | 1.00 | Y | 1p32.3 | DMRTA2 | 5' end |
| 2E36 | chr19:10597081-10597481 | 10597281 | 74 | 0.73 | Y | 19p13.2 | CTL2 | 5' end |
| 2E38 | chr9:125810419-125810819 | 125810619 | 66 | 0.93 | Y | 9q33.3 | LHX2 | 5' end |
| 2E40 | chr3:37878590-37878990 | 37878790 | 78 | 0.84 | Y | 3p22.3 | CTDSPL | Body |
| 2E47 | chr11:94439853-94440253 | 94440053 | 69 | 1.06 | Y | 11q21 | SRP46 | 5' end |
| 2E48 | chr1:209818515-209818915 | 209818715 | 78 | 0.94 | Y | 1q32.3 | SLC30A1 | 5' end |
| 2E49 | chr5:129268114-129268514 | 129268314 | 76 | 0.95 | Y | 5q23.3 | CSS3 | 5' end |
| 2E50 | chr12:30245666-30246066 | 30245866 | 64 | 0.76 | Y | 12p11.22 | spafy | 5' end |
| 2E52 | chr14:102611575-102611975 | 102611775 | 74 | 0.84 | Y | 14q32.32 | NA | Intergenic |
| 2E53 | chr15:54072556-54072956 | 54072756 | 72 | 0.87 | Y | 15q21.3 | NEDD4 | Body |
| 2E54 | chr20:56899506-56899906 | 56899706 | 52 | 1.19 | Y | 20q13.32 | GNAS | 5' end |
| 2E55 | chr14:93710290-93710690 | 93710490 | 77 | 0.87 | Y | 14q32.12 | KIAA1622 | 5' end |
| 2E56 | chr7:156485403-156485803 | 156485603 | 66 | 0.92 | Y | 7q36.3 | chawzerby | 5' end |
| 2E58 | chr2:176751052-176751452 | 176751252 | 63 | 0.94 | Y | 2q31.1 | BC030713 | 5' end |
| 2E64 | chr12:113370724-113371124 | 113370924 | 65 | 0.77 | Y | 12q24.21 | NA | Intergenic |
| 2E66 | chr6:132170801-132171201 | 132171001 | 79 | 1.00 | Y | 6q23.2 | ENPP1 | 5' end |
| 2E68 | chr2:72226257-72226657 | 72226457 | 69 | 0.73 | Y | 2p13.2 | P450RAI2 | Body |
| 2E69 | chr22:29082700-29083100 | 29082900 | 74 | 1.04 | Y | 22q12.2 | SF3A1 | 5' end |
| 2F01 | chr9:138414381-138414781 | 138414581 | 71 | 0.97 | Y | 9q34.3 | SNAPC4 | 5' end |
| 2F02 | chr11:7965545-7965945 | 7965745 | 62 | 0.68 | Y | 11p15.4 | EIF3S5 | 5' end |
| 2F04 | chr3:53054574-53054974 | 53054774 | 53 | 1.32 | Y | 3p21.1 | SFMBT1 | 5' end |
| 2F10 | chr2:237658896-237659296 | 237659096 | 61 | 1.15 | Y | 2q37.3 | COPS8 | 5' end |
| 2F11 | chr17:44024885-44025285 | 44025085 | 55 | 0.56 | N | 17q21.32 | HOXB5 | Body |
| 2F14 | chr4:167013947-167014347 | 167014147 | 70 | 0.89 | Y | 4q32.3 | TLL1 | 5' end |
| 2F15 | chr17:75398454-75398854 | 75398654 | 73 | 1.10 | Y | 17q25.3 | CBX2 | Body |
| 2F17 | chr6:43036552-43036952 | 43036752 | 69 | 0.70 | Y | 6p21.1 | GNMT | Body |
| 2F24 | chr9:99656511-99656911 | 99656711 | 80 | 1.08 | Y | 9q22.33 | FOXE1 | 5' end |
| 2F25 | chr17:34157252-34157652 | 34157452 | 76 | 0.76 | Y | 17q12 | PCGF2 | 5' end |
| 2F26 | chr20:10602067-10602467 | 10602267 | 66 | 1.03 | Y | 20p12.2 | JAG1 | 5' end |
| 2F29 | chr5:156209838-156210238 | 156210038 | 68 | 0.85 | Y | 5q33.3 |  | 5' end |
| 2F31 | chr12:47396922-47397322 | 47397122 | 59 | 1.08 | Y | 12q13.11 | CCNT1 | 5' end |
| 2F32 | chr1:233880286-233880686 | 233880486 | 73 | 0.91 | Y | 1q42.3 | GNG4 | 5' end |
| 2F34 | chr2:225615548-225615948 | 225615748 | 71 | 0.83 | Y | 2q36.2 | DOCK10 | 5' end |
| 2F36 | chr17:7152065-7152465 | 7152265 | 66 | 1.01 | Y | 17p13.1 | EIF5A | 5' end |
| 2F38 | chr15:63365592-63365992 | 63365792 | 70 | 0.81 | Y | 15q22.31 | FLJ20509 | 5' end |
| 2F40 | chr12:48026583-48026983 | 48026783 | 67 | 0.74 | Y | 12q13.12 | FLJ13236 | 5' end |
| 2F41_42 | chr12:3818306-3818706 | 3818506 | 74 | 1.00 | Y | 12p13.32 | C12orf6 | 5' end |
| 2F43 | chr19:63599542-63599942 | 63599742 | 79 | 1.13 | Y | 19q13.43 | FLJ39005 | 5' end |
| 2F44_45 | chr6:112300848-112301248 | 112301048 | 80 | 1.04 | Y | 6q21 | FYN | 5' end |
| 2F47 | chr1:22341839-22342239 | 22342039 | 57 | 1.12 | Y | 1p36.12 | WNT4 | 5' end |
| 2F48 | chr15:47700485-47700885 | 47700685 | 60 | 0.92 | Y | 15q21.2 | MDS009 | 5' end |
| 2F50 | chr1:38284700-38285100 | 38284900 | 80 | 1.07 | Y | 1p34.3 | POU3F1 | 5' end |
| 2F51 | chr11:69165791-69166191 | 69165991 | 61 | 0.82 | Y | 11q13.3 | CCND1 | 5' end |
| 2F54 | chr5:76961992-76962392 | 76962192 | 71 | 0.86 | Y | 5q14.1 | OTP | Body |
| 2F55 | chr6:152170740-152171140 | 152170940 | 67 | 0.76 | Y | 6q25.1 | ESR1 | 5' end |
| 2F57 | chr2:95556139-95556539 | 95556339 | 73 | 0.65 | Y | 2q11.1 | LOC390234 | 5' end |
| 2F58 | chr19:52307255-52307655 | 52307455 | 72 | 0.87 | Y | 19q13.32 | C19orf7 | 5' end |
| 2F59 | chr3:36961005-36961405 | 36961205 | 75 | 0.90 | Y | 3p22.3 | KIAA0342.bNov04 | 5' end |
| 2F60 | chr2:232353769-232354169 | 232353969 | 73 | 0.84 | Y | 2q37.1 | PDE6D | Body |
| 2F62 | chr8:61754633-61755033 | 61754833 | 78 | 1.20 | Y | 8q12.2 | CHD7 | 5' end |
| 2F63_64 | chr11:115135732-115136132 | 115135932 | 72 | 0.79 | Y | 11q23.3 | AK090602 | Body |
| 2F68 | chr14:23850460-23850860 | 23850660 | 67 | 0.69 | Y | 14q11.2 | CIDEB | 5' end |
| 2F68 | chr14:23850460-23850860 | 23850660 | -- | -- | N | 19p13.3 | BC048290 | Body |
| 2F69 | chr15:63457240-63457640 | 63457440 | 78 | 0.85 | Y | 15q22.31 | PUNC | 5' end |
| 2F70 | chr2:161809737-161810137 | 161809937 | 64 | 0.82 | Y | 2q24.2 | AK027541 | Body |
| 2F71 | chr16:85157926-85158326 | 85158126 | 76 | 0.91 | Y | 16q24.1 | FOXC2 | 5' end |
| 2F72 | chr3:185217837-185218237 | 185218037 | 73 | 0.77 | Y | 3q27.1 | ABCC5 | 5' end |
| 2F74 | chr9:19220517-19220917 | 19220717 | 75 | 0.92 | Y | 9p22.1 | AK022061 | 5' end |
| 2F75 | chr2:210344882-210345282 | 210345082 | 72 | 0.64 | Y | 2q34 | C2orf21 | 5' end |
| 2F76 | chr3:44011462-44011862 | 44011662 | 72 | 0.80 | Y | 3p21.33 | NA | Intergenic |
| 2F77 | chr13:101850696-101851096 | 101850896 | 64 | 0.66 | Y | 13q33.1 | FGF14 | Body |
| 2F79 | chr22:37569757-37570157 | 37569957 | 83 | 0.93 | Y | 22q13.1 | NPTXR | 5' end |
| 2F81 | chr6:78230199-78230599 | 78230399 | 67 | 0.68 | Y | 6q14.1 | HTR1B | 5' end |
| 2G06 | chr6:24603207-24603607 | 24603407 | 76 | 0.86 | Y | 6p22.2 | ALDH5A1 | 5' end |
| 2G10 | chr7:35973484-35973884 | 35973684 | 68 | 0.74 | Y | 7p14.2 | SEPT7 | Body |
| 2G100 | chr3:18461540-18461940 | 18461740 | 81 | 0.92 | Y | 3p24.3 | TBC1D5 | 5' end |
| 2G102 | chr11:20365594-20365994 | 20365794 | 75 | 1.00 | Y | 11p15.1 | HRMT1L3 | 5' end |
| 2G108 | chr3:151963492-151963892 | 151963692 | 77 | 0.89 | Y | 3q25.1 | SIAH2 | 5' end |
| 2G42 | chr9:87745574-87745974 | 87745774 | 67 | 1.10 | Y | 9q21.33 | FLJ21613 | 5' end |
| 2G44 | chr5:96023663-96024063 | 96023863 | 76 | 0.74 | Y | 5q15 | CAST | 5' end |
| 2G54 | chr7:7572942-7573342 | 7573142 | 77 | 0.98 | Y | 7p21.3 | FLJ20323 | 5' end |
| 2G72 | chr9:76893027-76893427 | 76893227 | 73 | 1.05 | Y | 9q21.13 | OSTF1;C9orf95 | 5' end |
| 2G80 | chr10:102473840-102474240 | 102474040 | 55 | 1.03 | Y | 10q24.31 | NA | Intergenic |
| 2G89 | chr11:27450237-27450637 | 27450437 | 78 | 0.95 | Y | 11p14.1 | LGR4 | 5' end |
| 2G93 | chr12:111340718-111341118 | 111340918 | 64 | 1.14 | Y | 12q24.13 | PTPN11 | 5' end |
| 2G97 | chr12:13322344-13322744 | 13322544 | 57 | 0.69 | Y | 12p13.1 | NA | Intergenic |
| 3A05 | chr19:56535059-56535459 | 56535259 | 75 | 1.04 | Y | 19q13.41 | BC032383 | 5' end |
| 3A15 | chr2:236810253-236810653 | 236810453 | 61 | 0.72 | Y | 2q37.2 | AK123854 | Body |
| 3A24 | chr11:128654548-128654948 | 128654748 | 81 | 1.00 | Y | 11q24.3 | AK127847 | Body |
| 3A25 | chr11:57000311-57000711 | 57000511 | 75 | 1.04 | Y | 11q12.1 | RTN4RL2 | Body |
| 3B04 | chr21:44543910-44544310 | 44544110 | 73 | 0.71 | Y | 21q22.3 | PFKL | 5' end |
| 3B05 | chr2:238813174-238813574 | 238813374 | 77 | 1.01 | Y | 2q37.3 | HES6 | 5' end |
| 3B06 | chr7:148787388-148787788 | 148787588 | 73 | 0.70 | Y | 7q36.1 | KIAA1285 | 5' end |
| 3B07 | chr7:154975702-154976102 | 154975902 | -- | -- | N | 7q36.3 | AW297462 | 5' end |
| 3B09 | chr1:54726570-54726970 | 54726770 | 69 | 0.66 | Y | 1p32.3 | AF416921 | Body |
| 3B10 | chr7:64853141-64853541 | 64853341 | 69 | 0.87 | Y | 7q11.21 | CCT6A | 5' end |
| 3B19 | chr2:23461579-23461979 | 23461779 | 53 | 1.11 | Y | 2p24.1 | BC013982 | 5' end |
| 3B20 | chr9:94860655-94861055 | 94860855 | 76 | 0.67 | Y | 9q22.31 | MGC26847 | 5' end |
| 3B21 | chr13:31503468-31503868 | 31503668 | 67 | 0.78 | Y | 13q13.1 | 13CDNA73 | 5' end |
| 3B22 | chr11:57091840-57092240 | 57092040 | 70 | 0.95 | Y | 11q12.1 | UBE2L6 | 5' end |
| 3B24 | chr2:118784031-118784431 | 118784231 | 51 | 1.36 | Y | 2q14.2 | NA | Intergenic |
| 3B25 | chr1:224661797-224662197 | 224661997 | 65 | 0.81 | Y | 1q42.12 | PARP1 | Body |
| 3B27 | chr2:61550932-61551332 | 61551132 | 71 | 0.98 | Y | 2p15 | USP34 | Body |
| 3B30 | chr15:40816055-40816455 | 40816255 | 74 | 0.79 | Y | 15q15.2 | CDAN1 | Body |
| 3B31 | chr19:3012128-3012528 | 3012328 | 83 | 0.92 | Y | 19p13.3 | AES | Body |
| 3B32 | chr3:127880742-127881142 | 127880942 | 65 | 0.68 | Y | 3q21.3 | NA | Intergenic |
| 3B35 | chr8:128818613-128819013 | 128818813 | 57 | 0.76 | Y | 8q24.21 | MYC | 5' end |
| 3B36 | chr2:38155453-38155853 | 38155653 | 72 | 1.02 | Y | 2p22.2 | CYP1B1 | Body |
| 3B37 | chr7:121300263-121300663 | 121300463 | 69 | 0.97 | Y | 7q31.32 | PTPRZ1 | 5' end |
| 3B38 | chr5:132183126-132183526 | 132183326 | 61 | 0.97 | Y | 5q23.3 | -- | Intergenic |
| 3B41 | chr9:99303557-99303957 | 99303757 | 83 | 0.92 | Y | 9q22.33 | BC002660 | 5' end |
| 3B42 | chr9:102154811-102155211 | 102155011 | 71 | 0.92 | Y | 9q31.1 | TEX10 | 5' end |
| 3B44 | chr2:128138318-128138718 | 128138518 | 75 | 0.91 | Y | 2q14.3 | LIMS2 | 5' end |
| 3B45 | chr19:61571326-61571726 | 61571526 | 66 | 0.90 | Y | 19q13.43 | ZNF542 | 5' end |
| 3B46 | chr2:71952217-71952617 | 71952417 | low | low | N | 2p13.2 | NA | Intergenic |
| 3B47 | chr18:74839624-74840024 | 74839824 | 52 | 1.16 | Y | 18q.23 | SALL3 | 5' end |
| 3B48 | chr20:30635817-30636217 | 30636017 | 75 | 0.94 | Y | 20q11.21 | BC023657 | 5' end |
| 3B50 | chr17:41053766-41054166 | 41053966 | 77 | 0.75 | Y | 17q21.31 | BC012843 | 5' end |
| 3B52 | chr1:153201119-153201519 | 153201319 | 65 | 0.80 | Y | 1q22 | PYGO2 | 5' end |
| 3B53 | chr1:41480106-41480506 | 41480306 | 80 | 0.85 | Y | 1p34.2 | SCMH1 | 5' end |
| 3B54 | chr9:113699078-113699478 | 113699278 | 73 | 0.86 | Y | 9q31.3 | UGCG | 5' end |
| 3B55 | chr2:161988450-161988850 | 161988650 | 73 | 1.04 | Y | 2q24.2 | TBR1 | Body |
| 3B58 | chr18:19972714-19973114 | 19972914 | 64 | 0.83 | Y | 18q11.2 | CABYR | 5' end |
| 3B60 | chr17:1029790-1030190 | 1029990 | 77 | 0.88 | Y | 17p13.3 | ABR | 5' end |
| 3C01 | chr18:11741452-11741852 | 11741652 | 70 | 0.81 | Y | 18p11.21 | GNAL | 5' end |
| 3C02 | chr5:3649107-3649507 | 3649307 | 77 | 1.03 | Y | 5p15.33 | IRX1 | 5' end |
| 3C05 | chr16:51723130-51723530 | 51723330 | 69 | 0.82 | Y | 16q12.2 | CHD9 | Body |
| 3C07 | chr4:105631967-105632367 | 105632167 | 74 | 0.90 | Y | 4q24 | CXXC4 | 5' end |
| 3C08_09 | chr9:132961291-132961691 | 132961491 | 68 | 0.71 | Y | 9q34.13 | C9orf58 | 5' end |
| 3C10 | chr12:100748556-100748956 | 100748756 | 79 | 1.02 | Y | 12q23.2 | MGC4170 | 5' end |
| 3C11 | chr6:152999480-152999880 | 152999680 | 74 | 0.88 | Y | 6q25.2 | SYNE1 | 5' end |
| 3C14 | chr22:30670286-30670686 | 30670486 | 76 | 1.04 | Y | 22q12.3 | HSN44A4A | 5' end |
| 3C16 | chr7:44754661-44755061 | 44754861 | 79 | 0.93 | Y | 7p13 | DKFZp761I2123 | 5' end |
| 3C17 | chr6:72186484-72186884 | 72186684 | 77 | 1.09 | Y | 6q13 | C6orf155 | 5' end |
| 3C19 | chr4:184255403-184255803 | 184255603 | 56 | 0.58 | N | 4q35.1 | FLJ30277 | Body |
| 3C25 | chr2:10969555-10969955 | 10969755 | 80 | 0.91 | Y | 2p25.1 | KCNF1 | 5' end |
| 3C30 | chr10:74676913-74677313 | 74677113 | 62 | 0.97 | Y | 10q22.2 | DNAJC9 | 5' end |
| 3C31 | chr10:28071036-28071436 | 28071236 | 70 | 0.84 | Y | 10p12.1 | C10orf48 | Body |
| 3C32 | chr1:179148235-179148635 | 179148435 | 77 | 0.86 | Y | 1q25.3 | AB046834 | 5' end |
| 3C34 | chr12:56545392-56545792 | 56545592 | 72 | 0.83 | Y | 12q14.1 | NA | Intergenic |
| 3C35 | chr22:34754382-34754782 | 34754582 | 81 | 1.12 | Y | 22q12.3 | RBM9 | 5' end |
| 3C36 | chr19:14445506-14445906 | 14445706 | 76 | 0.91 | Y | 19p13.12 | PTGER1 | Body |
| 3C38 | chr7:130662983-130663383 | 130663183 | 69 | 0.84 | Y | 7q32.3 | MKLN1 | 5' end |
| 3C40 | chr7:157176087-157176487 | 157176287 | 77 | 1.03 | Y | 7q36.3 | PTPRN2 | Body |
| 3C42 | chr4:1390130-1390530 | 1390330 | 79 | 1.05 | Y | 4p16.3 | HSPX153 | 5' end |
| 3C45 | chr15:81666877-81667277 | 81667077 | 75 | 0.97 | Y | 15q25.2 | HDGFRP3 | Body |
| 3C52 | chr10:70257469-70257869 | 70257669 | 73 | 0.99 | Y | 10q21.3 | STOX1 | 5' end |
| 3C53 | chr12:48025197-48025597 | 48025397 | 67 | 0.74 | Y | 12q13.12 | FLJ13236 | 5' end |
| 3C54 | chr14:74029574-74029974 | 74029774 | 70 | 0.69 | Y | 14q24.3 | HBLD1 | 5' end |
| 3C55 | chr10:47125632-47126032 | 47125832 | 70 | 0.76 | Y | 10q11.22 | BC031882 | 5' end |
| 3C57 | chr13:39644234-39644634 | 39644434 | 64 | 0.67 | Y | 13q14.11 | NA | Intergenic |
| 3C60 | chr16:67039658-67040058 | 67039858 | 65 | 0.73 | Y | 16q22.1 | SMPD3 | 5' end |
| 3C61 | chr7:87343530-87343930 | 87343730 | 70 | 1.16 | Y | 7q21.12 | ASK;MCFP | 5' end |
| 3C62 | chr4:83513913-83514313 | 83514113 | 64 | 1.25 | Y | 4q21.22 | HNRPD | 5' end |
| 3C63 | chr4:186554439-186554839 | 186554639 | 64 | 0.99 | Y | 4q35.1 | ANKRD37 | 5' end |
| 3C64 | chr7:1253663-1254063 | 1253863 | 75 | 0.67 | Y | 7p22.3 | NA | Intergenic |
| 3C65 | chr17:46213605-46214005 | 46213805 | 62 | 0.91 | Y | 17q21.33 | NA | Intergenic |
| 3C67 | chr9:129537101-129537501 | 129537301 | 74 | 0.90 | Y | 9q34.11 | TOR2A | 5' end |
| 3C68 | chr20:42947873-42948273 | 42948073 | 72 | 0.85 | Y | 20q13.12 | YWHAB | 5' end |
| 3C70 | chr12:122321009-122321409 | 122321209 | 73 | 0.66 | Y | 12q24.31 | CDK2AP1 | Body |
| 3C71 | chr22:20335888-20336288 | 20336088 | 60 | 0.71 | Y | 22q11.21 | THC2153079 | 5' end |
| 3C73 | chr19:51844567-51844967 | 51844767 | 72 | 1.04 | Y | 19q13.32 | MGC15476 | Body |
| 3C74 | chr5:81082153-81082553 | 81082353 | 70 | 0.81 | Y | 5q14.1 | SSBP2 | Body |
| 3C75 | chr7:157174969-157175369 | 157175169 | 60 | 0.68 | Y | 7q36.3 | PTPRN2 | Body |
| 3C76 | chr12:122322149-122322549 | 122322349 | 83 | 1.09 | Y | 12q24.31 | CDK2AP1 | 5' end |
| 3C77 | chr1:3556083-3556483 | 3556283 | 70 | 0.70 | Y | 1p36.32 | WDR8 | 5' end |
| 3D01_2D73 | chr5:140978793-140979193 | 140978993 | 64 | 0.81 | Y | 5q31.3 | DIAPH1 | 5' end |
| 3D04 | chr15:65908474-65908874 | 65908674 | 60 | 0.90 | Y | 15q23 | LOC390598 | 5' end |
| 3D05_06 | chr8:102034274-102034674 | 102034474 | 63 | 0.84 | Y | 8q22.3 | YWHAZ | 5' end |
| 3D07 | chr17:1848254-1848654 | 1848454 | 77 | 0.88 | Y | 17p13.3 | RTN4RL1 | Body |
| 3D09 | chr19:50270718-50271118 | 50270918 | 67 | 0.56 | N | 19q13.32 | ZNF342 | 5' end |
| 3D10 | chr1:26671711-26672111 | 26671911 | 76 | 0.88 | Y | 1p36.11 | HMGN2 | Body |
| 3D11 | chr1:21850784-21851184 | 21850984 | 82 | 1.03 | Y | 1p36.12 | RAP1GA1 | 5' end |
| 3D12 | chr5:61638040-61638440 | 61638240 | 65 | 0.99 | Y | 5q12.1 | KIF2 | 5' end |
| 3D13 | chr4:981145-981545 | 981345 | 79 | 0.92 | Y | 4p16.3 | IDUA | 5' end |
| 3D14 | chr14:28305596-28305996 | 28305796 | 66 | 0.79 | Y | 14q12 | FOXG1B | 5' end |
| 3D15 | chr2:166358057-166358457 | 166358257 | 58 | 1.16 | Y | 5q35.2 | HRH2 | 5' end |
| 3D15 | chr2:166358057-166358457 | 166358257 | 61 | 0.72 | Y | 2q24.3 | GALNT3 | Body |
| 3D16 | chr1:160797719-160798119 | 160797919 | 72 | 0.90 | Y | 1q23.3 | UAP1 | 5' end |
| 3D17 | chr3:178398005-178398405 | 178398205 | 81 | 0.98 | Y | 3q26.32 | TBL1XR1 | 5' end |
| 3D18 | chr17:33982366-33982766 | 33982566 | 80 | 0.91 | Y | 17q12 | SNIP | Body |
| 3D19 | chr2:70166720-70167120 | 70166920 | 55 | 0.86 | Y | 2p13.3 | PCBP1 | 5' end |
| 3D21 | chr3:180523362-180523762 | 180523562 | 81 | 0.87 | Y | 3q26.32 | ZNF639 | 5' end |
| 3D23 | chr10:46333883-46334283 | 46334083 | 69 | 1.09 | Y | 10q11.22 | RHEBP1.cNov04 | 5' end |
| 3D24 | chr17:55534607-55535007 | 55534807 | 54 | 0.89 | Y | 17q23.2 | THC2173136_2 | 5' end |
| 3D25 | chr18:19523459-19523859 | 19523659 | 76 | 0.86 | Y | 18q11.2 | LAMA3 | 5' end |
| 3D28 | chr18:55515425-55515825 | 55515625 | 69 | 0.67 | Y | 18q21.32 | CCBE1 | 5' end |
| 3D29 | chr8:118601959-118602359 | 118602159 | 66 | 0.79 | Y | 8q24.11 | THRAP6 | 5' end |
| 3D31 | chr13:28900392-28900792 | 28900592 | 70 | 0.60 | Y | 13q12.3 | BC032481 | 5' end |
| 3D33 | chr7:27175662-27176062 | 27175862 | 66 | 0.72 | Y | 7p15.2 | HOXA10; HOXA9 | 5' end |
| 3D34 | chr7:72907792-72908192 | 72907992 | 66 | 0.68 | Y | 7q11.23 | NA | Intergenic |
| 3D35 | chr6:85529922-85530322 | 85530122 | 63 | 0.92 | Y | 6q14.3 | TBX18 | 5' end |
| 3D36 | chr19:5518643-5519043 | 5518843 | 77 | 0.99 | Y | 19p13.3 | PLAC2 | 5' end |
| 3D40 | chr18:4445197-4445597 | 4445397 | 77 | 0.94 | Y | 18p11.31 | LOC388458.aNov04 | 5' end |
| 3D41 | chr12:100127408-100127808 | 100127608 | 63 | 0.85 | Y | 12q23.2 | SLC5A8 | 5' end |
| 3D42 | chr20:60131048-60131448 | 60131248 | 76 | 0.95 | Y | 20q13.33 | C20orf40 | 5' end |
| 3D44 | chr6:143289464-143289864 | 143289664 | 73 | 0.85 | Y | 6q24.2 | HIVEP2 | Body |
| 3D46 | chr17:69861884-69862284 | 69862084 | 68 | 0.67 | Y | 17q25.1 | AK090911 | 5' end |
| 3D48 | chr17:26322351-26322751 | 26322551 | 70 | 0.90 | Y | 17q11.2 | RNF135 | 5' end |
| 3D48 | chr17:26322351-26322751 | 26322551 | 68 | 0.87 | Y | 17q11.2 | RNF135 | 5' end |
| 3D49 | chr19:1305880-1306280 | 1306080 | 82 | 0.95 | Y | 19p13.3 | MUM1 | 5' end |
| 3D50 | chr13:41512335-41512735 | 41512535 | 77 | 0.91 | Y | 13q14.11 | NA | Intergenic |
| 3D51 | chr13:79813232-79813632 | 79813432 | 70 | 0.88 | Y | 13q31.1 | SPRY2 | 5' end |
| 3D52 | chr8:140785266-140785666 | 140785466 | 80 | 1.03 | Y | 8q24.3 | KCNK9 | 5' end |
| 3D55 | chr9:81376421-81376821 | 81376621 | 54 | 0.87 | Y | 9q21.31 | TLE4 | 5' end |
| 3D56 | chr20:39379668-39380068 | 39379868 | 75 | 0.85 | Y | 20q12 | NA | Intergenic |
| 3D57 | chr10:124899009-124899409 | 124899209 | 61 | 0.87 | Y | 10q26.13 | BUB3 | 5' end |
| 3D58 | chr12:12769012-12769412 | 12769212 | 67 | 0.81 | Y | 12p13.2 | AL049239 | 5' end |
| 3D60 | chr1:100777989-100778389 | 100778189 | 71 | 0.88 | Y | 1p21.2 | GPR88 | Body |
| 3D61 | chr10:131824618-131825018 | 131824818 | 75 | 0.83 | Y | 10q26.3 | TXNL2 | Body |
| 3D62 | chr20:35589362-35589762 | 35589562 | 68 | 0.72 | Y | 20q11.23 | BLCAP | 5' end |
| 3D67 | chr14:70345924-70346324 | 70346124 | 77 | 0.89 | Y | 14q24.2 | MAP3K9 | 5' end |
| 3D68 | chr10:101284987-101285387 | 101285187 | 76 | 1.08 | Y | 10q24.2 | NKX2-3 | Body |
| 3D70 | chr13:27439016-27439416 | 27439216 | 74 | 0.95 | Y | 13q12.2 | CDX2 | Body |
| 3E03 | chr15:74425809-74426209 | 74426009 | 71 | 0.95 | Y | 15q24.3 | ZNF291 | Body |
| 3E04 | chr11:93917216-93917616 | 93917416 | 76 | 0.98 | Y | 11q21 | FUT4 | 5' end |
| 3E05 | chr1:24066909-24067309 | 24067109 | 71 | 0.91 | Y | 1p36.33 | LOC391202 | 5' end |
| 3E07 | chr2:171725557-171725957 | 171725757 | 79 | 1.03 | Y | 2q31.1 | TLK1 | 5' end |
| 3E12 | chr17:46692913-46693313 | 46693113 | 70 | 1.14 | Y | 17q21.33 | CGI-48 | 5' end |
| 3E13 | chr2:131230190-131230590 | 131230390 | 76 | 0.88 | Y | 2q21.1 | FLJ38377 | 5' end |
| 3E14 | chr11:74119881-74120281 | 74120081 | 77 | 0.95 | Y | 11q13.4 | BNF1 | 5' end |
| 3E15 | chr11:77963083-77963483 | 77963283 | 66 | 0.75 | Y | 11q14.1 | FLJ23441 | 5' end |
| 3E17 | chr7:106472127-106472527 | 106472327 | 82 | 0.96 | Y | 7q22.3 | PRKAR2B | 5' end |
| 3E22 | chr14:93710283-93710683 | 93710483 | 77 | 0.87 | Y | 14q32.12 | KIAA1622 | 5' end |
| 3E24 | chr3:185561758-185562158 | 185561958 | 72 | 0.84 | Y | 3q27.1 | CLCN2; POLR2H | 5' end |
| 3E25 | chr16:47402274-47402674 | 47402474 | 73 | 1.02 | Y | 16q12.1 | NA | Intergenic |
| 3E27 | chr10:131824618-131825018 | 131824818 | 75 | 0.83 | Y | 10q26.3 | TXNL2 | Body |
| 3E29 | chr22:49025798-49026198 | 49025998 | 71 | 0.78 | Y | 22q13.33 | HDAC10;TUBGCP6 | 5' end |
| 3E30 | chr6:166586758-166587158 | 166586958 | 61 | 0.86 | Y | 6q27 | BC040873 | Body |
| 3E31 | chr6:149109751-149110151 | 149109951 | 72 | 0.95 | Y | 6q25.1 | UST | 5' end |
| 3E33 | chr2:128175440-128175840 | 128175640 | 75 | 1.01 | Y | 2q14.3 | MGC5391 | 5' end |
| 3E34 | chr22:17814909-17815309 | 17815109 | 71 | 1.00 | Y | 22q11.21 | LOC128977 | Body |
| 3E35 | chr5:131774387-131774787 | 131774587 | 74 | 0.95 | Y | 5q23.3 | AK096941 | 5' end |
| 3E39 | chr3:15348914-15349314 | 15349114 | 72 | 0.98 | Y | 3p25.1 | SH3BP5 | 5' end |
| 3E40 | chr10:88381543-88381943 | 88381743 | 55 | 0.58 | N | 10q23.2 | NA | Intergenic |
| 3E42_43 | chr9:91115151-91115551 | 91115351 | 69 | 0.85 | Y | 9q22.2 | CKS2 | 5' end |
| 3E45 | chr8:25956764-25957164 | 25956964 | 60 | 0.84 | Y | 8p21.2 | COE2 | Body |
| 3E48 | chr11:33848437-33848837 | 33848637 | 67 | 0.72 | Y | 11p13 | LMO2 | 5' end |
| 3E50 | chr16:3447720-3448120 | 3447920 | 80 | 0.87 | Y | 16p13.3 | FLJ14154 | 5' end |
| 3E52 | chr3:42995658-42996058 | 42995858 | 64 | 0.75 | Y | 3p22.1 | DKFZP434B172 | 5' end |
| 3E53 | chr9:16860504-16860904 | 16860704 | 72 | 0.97 | Y | 9p22.2 | BNC2 | 5' end |
| 3E54 | chr2:104825594-104825994 | 104825794 | 64 | 0.82 | Y | 2q12.1 | AK096498 | Body |
| 3E55 | chr13:27396617-27397017 | 27396817 | 73 | 0.77 | Y | 13q12.2 | IPF1 | Body |
| 3E56 | chr7:43912544-43912944 | 43912744 | 81 | 0.76 | Y | 7p13 | URG4 | 5' end |
| 3E57 | chr5:155087924-155088324 | 155088124 | 73 | 0.80 | Y | 5q33.2 | NA | Intergenic |
| 3E58 | chr3:185217830-185218230 | 185218030 | 73 | 0.77 | Y | 3q27.1 | ABCC5 | 5' end |
| 3E59 | chr19:54636323-54636723 | 54636523 | 76 | 0.74 | Y | 19q13.33 | SLC17A7 | 5' end |
| 3E60 | chr8:1909230-1909630 | 1909430 | 81 | 0.91 | Y | 8p23.3 | KIAA0711 | 5' end |
| 3E65 | chr4:48187570-48187970 | 48187770 | 78 | 1.06 | Y | 4p12 | ZAR1 | 5' end |
| 3E66 | chr14:49399323-49399723 | 49399523 | 57 | 0.96 | Y | 14q21.3 | AF068289 | 5' end |
| 3E67 | chr1:11255703-11256103 | 11255903 | 75 | 0.72 | Y | 1p36.22 | TERE1 | 5' end |
| 3E68 | chr12:51554145-51554545 | 51554345 | 74 | 0.75 | Y | 12q13.13 | NA | Intergenic |
| 3E69 | chr1:210847095-210847495 | 210847295 | 72 | 0.81 | Y | 1q32.3 | ATF3 | 5' end |
| 3E70 | chr9:124030971-124031371 | 124031171 | 67 | 0.65 | Y | 9q33.2 | LHX6 | 5' end |
| 3E71 | chr9:113285297-113285697 | 113285497 | 78 | 0.90 | Y | 9q31.3 | AK122712 | Body |
| 3F01 | chr1:19863846-19864246 | 19864046 | 82 | 0.93 | Y | 1p36.13 | HTR6 | 5' end |
| 3F02 | chr6:28818575-28818975 | 28818775 | 51 | 0.98 | Y | 6p22.1 | NA | Intergenic |
| 3F04 | chr15:92245393-92245793 | 92245593 | low | low | N | 15q26.2 | BC021741 | 5' end |
| 3F05 | chr3:100102600-100103000 | 100102800 | 73 | 0.77 | Y | 3q12.1 | ESDN | 5' end |
| 3F07 | chr19:63508307-63508707 | 63508507 | 63 | 0.76 | Y | 19q13.43 | LOC113386 | 5' end |
| 3F10 | chr2:236752479-236752879 | 236752679 | 49 | 1.06 | N | 2q37.2 | NA | Intergenic |
| 3F11 | chr3:125786019-125786419 | 125786219 | 71 | 0.69 | Y | 3q21.2 | TRAD | 5' end |
| 3F13 | chr1:69810232-69810632 | 69810432 | 66 | 0.83 | Y | 1p31.1 | LRRC7 | Body |
| 3F16 | chr10:131652421-131652821 | 131652621 | 78 | 1.12 | Y | 10q26.3 | AL832828 | 5' end |
| 3F17 | chr5:72779270-72779670 | 72779470 | 69 | 1.03 | Y | 5q13.2 | FOXD1 | 5' end |
| 3F18 | chr1:149697860-149698260 | 149698060 | 80 | 1.11 | Y | 1q21.3 | POGZ | 5' end |
| 3F20 | chr11:63809966-63810366 | 63810166 | 69 | 0.59 | N | 11q13.1 | C11ORF4 | 5' end |
| 3F24 | chr18:20260758-20261158 | 20260958 | 66 | 0.92 | Y | 18q11.2 | IMPACT | Body |
| 3F26 | chr12:439589-439989 | 439789 | 60 | 0.98 | Y | 12p13.33 | B4GALNT3 | 5' end |
| 3F30 | chr22:23837182-23837582 | 23837382 | 66 | 0.62 | Y | 22q11.23 | AF015910 | 5' end |
| 3F33 | chr2:227736886-227737286 | 227737086 | 76 | 0.80 | Y | 2q36.3 | COL4A4 | 5' end |
| 3F34 | chr4:141896721-141897121 | 141896921 | 76 | 0.92 | Y | 4q31.21 | AB020689 | 5' end |
| 3F44 | chr20:47965105-47965505 | 47965305 | 73 | 0.75 | Y | 20q13.13 | SPATA2 | 5' end |
| 3F48 | chr15:98698657-98699057 | 98698857 | 60 | 1.08 | Y | 15q26.3 | ADAMTS17 | 5' end |
| 3F50 | chr10:102891669-102892069 | 102891869 | 50 | 0.64 | Y | 10q24.31 | NA | Intergenic |
| 3F53 | chr18:40512916-40513316 | 40513116 | 59 | 0.89 | Y | 18q12.3 | SETBP1 | 5' end |
| 3F55 | chr8:66916146-66916546 | 66916346 | 76 | 0.89 | Y | 8q13.1 | PDE7A | 5' end |
| 3F62 | chr12:52709540-52709940 | 52709740 | 58 | 0.88 | Y | 12q13.13 | HOXC6 | 5' end |
| 3F63 | chr1:154593220-154593620 | 154593420 | 71 | 0.61 | Y | 1q22 | NA | Intergenic |
| 3F64 | chr4:915596-915996 | 915796 | 73 | 0.88 | Y | 4p16.3 | GAK | 5' end |
| 3F68 | chr17:14145223-14145623 | 14145423 | 62 | 0.83 | Y | 17p12 | HS3ST3B1 | 5' end |
| 3F71 | chr12:51371082-51371482 | 51371282 | 65 | 0.87 | Y | 12q13.13 | KRT1B | Body |
| 3F72 | chr4:119492606-119493006 | 119492806 | 64 | 0.74 | Y | 4q26 | PRSS12 | Body |
| 3F73 | chr11:44905223-44905623 | 44905423 | low | low | N | 11p11.2 | TP53I11 | Body |
| 3F74 | chr2:108769450-108769850 | 108769650 | 67 | 0.95 | Y | 2q12.3 | FLJ32745 | 5' end |
| 3F75 | chr6:83834048-83834448 | 83834248 | 71 | 0.88 | Y | 6q14.1 | KIAA1117 | 5' end |
| 3F80 | chr22:44643153-44643553 | 44643353 | low | low | N | 22q13.31 | AI673633 | Intergenic |
| 3F81 | chr5:66335590-66335990 | 66335790 | 71 | 0.72 | Y | 5q12.3 | MAST4 | 5' end |
| 3F82 | chr16:31487921-31488321 | 31488121 | 63 | 0.99 | Y | 16p11.2 | kershey | Body |
| 3F83 | chr5:44425325-44425725 | 44425525 | 64 | 0.64 | Y | 5p12 | FGF10 | 5' end |
| 3G08 | chr4:101034506-101034906 | 101034706 | 63 | 0.89 | Y | 4q23 | MAP2K1IP1 | 5' end |
| 3G15 | chr1:179341020-179341420 | 179341220 | 75 | 0.94 | Y | 1q25.3 | THC2227874 | 5' end |
| 3G29 | chr2:232969097-232969497 | 232969297 | 51 | 0.61 | Y | 2q37.1 | NA | Intergenic |
| 3G33 | chr2:227736886-227737286 | 227737086 | 76 | 0.80 | Y | 2q36.3 | COL4A4 | 5' end |
| 3G38 | chr4:123967438-123967838 | 123967638 | 80 | 0.92 | Y | 4q27 | FGF2 | 5' end |
| 3G39 | chr13:71338371-71338771 | 71338571 | 75 | 0.96 | Y | 13q21.33 | DACH1 | 5' end |
| 3G42 | chr1:110950525-110950925 | 110950725 | 72 | 0.73 | Y | 1p13.3 | KCNA2 | 5' end |
| 3G45 | chr5:81083328-81083728 | 81083528 | 71 | 0.71 | Y | 5q14.1 | SSBP2 | 5' end |
| 3G46 | chr11:118477654-118478054 | 118477854 | 51 | 0.68 | Y | 11q23.3 | DPAGT1 | 5' end |
| 3G47 | chr6:139737187-139737587 | 139737387 | 64 | 1.01 | Y | 6q24.1 | CITED2 | 5' end |
| 3G66 | chr10:111673186-111673586 | 111673386 | 68 | 0.94 | Y | 10q25.1 | XPNPEP1 | 5' end |
| 3G67 | chr1:46686359-46686759 | 46686559 | 71 | 0.99 | Y | 1p33 | BC021746 | Body |
| 3G69 | chr1:226394464-226394864 | 226394664 | 71 | 0.97 | Y | 1q42.13 | GUK1 | 5' end |
| 3G70 | chr17:14148221-14148621 | 14148421 | 55 | 0.73 | Y | 17p12 | HS3ST3B1 | Body |
| 3G76_77 | chr10:28327614-28328014 | 28327814 | 75 | 0.92 | Y | 10p12.1 | ARMC4 | 5' end |
| 3G78 | chr1:47463932-47464332 | 47464132 | 72 | 0.88 | Y | 1p33 | TAL1 | Body |
| 3G91 | chr22:42910049-42910449 | 42910249 | low | low | N | 22q13.31 | AL590887 | 5' end |
| 4A01 | chr3:198865500-198865900 | 198865700 | 79 | 0.84 | Y | 3q29 | spawfoybo | 5' end |
| 4A04 | chr6:1555564-1555964 | 1555764 | 76 | 1.00 | Y | 6p25.3 | FOXC1 | 5' end |
| 4A07 | chr6:1555564-1555964 | 1555764 | 77 | 1.02 | Y | 6p25.3 | FOXC1 | 5' end |
| 4B01 | chr2:222996966-222997366 | 222997166 | 64 | 0.69 | Y | 2q36.1 | SGPP2 | 5' end |
| 4B02 | chr7:27231331-27231731 | 27231531 | 58 | 0.64 | Y | 7p15.2 | NA | Intergenic |
| 4B03 | chr18:74840513-74840913 | 74840713 | 53 | 1.16 | Y | 18q23 | SALL3 | 5' end |
| 4B04 | chr20:30634903-30635303 | 30635103 | 81 | 0.79 | Y | 20q11.21 | BC023657 | 5' end |
| 4B06 | chr14:92650798-92651198 | 92650998 | 80 | 1.03 | Y | 14q32.12 | ITPK1 | Body |
| 4B10 | chr15:32181122-32181522 | 32181322 | 64 | 0.72 | Y | 15q14 | LOC56851 | 5' end |
| 4B12 | chr6:91061487-91061887 | 91061687 | 73 | 0.98 | Y | 6q15 | BACH2 | 5' end |
| 4B13 | chr14:74663182-74663582 | 74663382 | 70 | 0.98 | Y | 14q24.3 | NEK9 | 5' end |
| 4B14 | chr2:95556132-95556532 | 95556332 | 73 | 0.65 | Y | 2q11.1 | LOC390234 | 5' end |
| 4B15 | chr16:23954123-23954523 | 23954323 | 62 | 0.54 | N | 16p12.2 | PRKCB1 | Body |
| 4B18 | chr7:5536431-5536831 | 5536631 | 78 | 1.01 | Y | 7p22.1 | ACTB | 5' end |
| 4B19 | chr2:72967810-72968210 | 72968010 | 74 | 0.85 | Y | 2p13.2 | SPR | 5' end |
| 4B21 | chr17:34157245-34157645 | 34157445 | 62 | 0.52 | N | 17q12 | PCGF2 | 5' end |
| 4B23 | chr5:95323035-95323435 | 95323235 | 70 | 0.93 | Y | 5q15 | ELL2 | 5' end |
| 4B25 | chr3:195971257-195971657 | 195971457 | 52 | 0.47 | N | 3q29 | BC042533 | Body |
| 4B26 | chr2:102338354-102338754 | 102338554 | 66 | 0.80 | Y | 2q11.2 | IL18R1 | 5' end |
| 4B28 | chr14:103057671-103058071 | 103057871 | 76 | 1.00 | Y | 14q32.32 | CKB | Body |
| 4B29 | chr8:124241818-124242218 | 124242018 | 73 | 0.76 | Y | 8q24.13 | NA | Intergenic |
| 4B30 | chr5:43156579-43156979 | 43156779 | 68 | 0.93 | Y | 5p12 | ZNF131 | 5' end |
| 4B31 | chr17:39786450-39786850 | 39786650 | 76 | 0.97 | Y | 17q21.31 | BC030200 | Body |
| 4B33 | chr18:59240151-59240551 | 59240351 | 58 | 0.89 | Y | 18q21.33 | VPS4B | Body |
| 4B35_36 | chr5:175017577-175017977 | 175017777 | 77 | 0.89 | Y | 5q35.2 | HRH2 | Body |
| 4B37 | chrX:10547824-10548224 | 10548024 | 49 | 0.74 | N | Xp22.22 | MID1 | 5' end |
| 4B38 | chr10:105871436-105871836 | 105871636 | 67 | 1.06 | Y | 10q25.1 | C10orf78 | 5' end |
| 4B39 | chr10:131660896-131661296 | 131661096 | 78 | 0.92 | Y | 10q26.3 | DKFZp667B0210 | 5' end |
| 4B40 | chr19:47124354-47124754 | 47124554 | 67 | 1.00 | Y | 19q13.2 | ARHGEF1 | Body |
| 4B42 | chr10:28072177-28072577 | 28072377 | 70 | 0.84 | Y | 10p12.1 | C10orf48 | 5' end |
| 4B43 | chr5:151131688-151132088 | 151131888 | 74 | 1.04 | Y | 5q33.1 | G3BP | 5' end |
| 4B44 | chr3:127725699-127726099 | 127725899 | 77 | 0.83 | Y | 3q21.3 | CHST13 | 5' end |
| 4B45 | chr7:148589919-148590319 | 148590119 | 77 | 1.02 | Y | 7q36.1 |  | 5' end |
| 4B46 | chr11:19323158-19323558 | 19323358 | 70 | 0.71 | Y | 11p15.1 | AY499135 | Body |
| 4B48 | chr12:12769019-12769419 | 12769219 | 67 | 0.81 | Y | 12p13.2 | AL049239 | 5' end |
| 4B53 | chr2:23461579-23461979 | 23461779 | 67 | 0.66 | Y | 2p24.1 | KBTBD9 | 5' end |
| 4B54 | chr16:1403636-1404036 | 1403836 | 64 | 0.84 | Y | 16p13.3 | UNKL | 5' end |
| 4B55 | chr16:11256117-11256517 | 11256317 | 66 | 0.97 | Y | 16p13.13 | SOCS1 | Body |
| 4B56 | chr3:45812697-45813097 | 45812897 | 69 | 1.08 | Y | 3p21.31 | SLC6A20 | 5' end |
| 4C03 | chr2:11402000-11402400 | 11402200 | 54 | 1.10 | Y | 2p25.1 | ROCK2 | 5' end |
| 4C05 | chr16:55836524-55836924 | 55836724 | 73 | 0.94 | Y | 16q13 | TM4SF11 | 5' end |
| 4C06 | chr10:64895252-64895652 | 64895452 | 67 | 1.02 | Y | 10q21.3 | JMJD1C | 5' end |
| 4C09 | chr3:9568877-9569277 | 9569077 | 63 | 0.65 | Y | 3p25.3 | LOC375323 | Body |
| 4C10 | chr11:74740061-74740461 | 74740261 | 77 | 0.89 | Y | 11q13.4 | ARRB1 | Body |
| 4C11 | chr11:13255545-13255945 | 13255745 | 70 | 0.89 | Y | 11p15.3 | ARNTL | 5' end |
| 4C14 | chr6:127878429-127878829 | 127878629 | 71 | 0.81 | Y | 6q22.33 | AK091416 | 5' end |
| 4C16 | chr16:88565887-88566287 | 88566087 | 64 | 0.73 | Y | 16q24.3 | AFG3L1 | 5' end |
| 4C17 | chr5:64434420-64434820 | 64434620 | 66 | 0.96 | Y | 5q12.3 | vojor | 5' end |
| 4C20 | chr2:15618749-15619149 | 15618949 | 54 | 0.67 | Y | 2p24.3 | NAG | 5' end |
| 4C21 | chr13:20533600-20534000 | 20533800 | 67 | 0.95 | Y | 13q12.11 | LATS2 | 5' end |
| 4C22 | chr16:84202917-84203317 | 84203117 | 74 | 0.75 | Y | 16q24.1 | D80004 | 5' end |
| 4C23 | chr2:16552914-16553314 | 16553114 | 74 | 0.79 | Y | 2p24.3 | NA | Intergenic |
| 4C25 | chr19:61317685-61318085 | 61317885 | 51 | 0.57 | N | 19q13.43 | BC012495 | 5' end |
| 4C26 | chr10:123862144-123862544 | 123862344 | 69 | 0.88 | Y | 10q26.13 | TACC2 | Body |
| 4C28 | chr4:82354966-82355366 | 82355166 | 73 | 0.98 | Y | 4q21.21 | PRKG2 | 5' end |
| 4C29 | chr20:36095256-36095656 | 36095456 | 66 | 0.97 | Y | 20q11.23 | C20orf77 | 5' end |
| 4C30 | chr19:38978001-38978401 | 38978201 | 72 | 0.87 | Y | 19q13.11 | KCTD15 | 5' end |
| 4C31 | chr19:19357639-19358039 | 19357839 | 76 | 1.11 | Y | 19p13.11 | AK025285 | Body |
| 4C32 | chr15:66510306-66510706 | 66510506 | 72 | 0.74 | Y | 15q23 | ITGA11 | Body |
| 4C33 | chr2:228045673-228046073 | 228045873 | 75 | 0.82 | Y | 2q36.3 | HRB | Body |
| 4C34 | chr16:52094237-52094637 | 52094437 | 74 | 0.76 | Y | 16q12.2 | FTS | 5' end |
| 4C42 | chr5:112851804-112852204 | 112852004 | 71 | 1.04 | Y | 5q22.2 | MCC | 5' end |
| 4C45 | chr17:44157680-44158080 | 44157880 | 59 | 0.72 | Y | 17q21.32 | HOXB13 | 5' end |
| 4C46 | chr4:166097583-166097983 | 166097783 | 63 | 0.76 | Y | 4q32.3 | FLJ31659 | 5' end |
| 4D01 | chr15:82978257-82978657 | 82978457 | 63 | 1.04 | Y | 15q25.3 | SCAND2 | Body |
| 4D07 | chr14:69109378-69109778 | 69109578 | 74 | 0.96 | Y | 14q24.1 | LOC387994 | 5' end |
| 4D08 | chr17:72952782-72953182 | 72952982 | low | low | N | 17q25.3 | SEPT9 | Body |
| 4D09 | chr6:31810939-31811339 | 31811139 | 51 | 0.77 | Y | 6p21.33 | CLIC1 | 5' end |
| 4D10 | chr7:32897345-32897745 | 32897545 | 80 | 0.93 | Y | 7p14.3 | KBTBD2 | 5' end |
| 4D11 | chr4:124538939-124539339 | 124539139 | 80 | 0.91 | Y | 4q28.1 | SPRY1 | 5' end |
| 4D12 | chr11:63531848-63532248 | 63532048 | 66 | 0.73 | Y | 11q13.1 | LRP16 | Body |
| 4D13 | chr6:105734505-105734905 | 105734705 | 69 | 1.02 | Y | 6q21 | POPDC3 | 5' end |
| 4D16 | chr10:5766716-5767116 | 5766916 | 69 | 0.92 | Y | 10p15.1 | AK126513 | 5' end |
| 4D17 | chr13:22938549-22938949 | 22938749 | 68 | 0.84 | Y | 13q12.12 | AL833146 | 5' end |
| 4D19 | chr19:1156744-1157144 | 1156944 | 62 | 0.98 | Y | 19p13.3 | STK11 | 5' end |
| 4D20 | chr20:31541560-31541960 | 31541760 | 77 | 1.15 | Y | 20q11.22 | AF052211 | 5' end |
| 4D22 | chr2:72998593-72998993 | 72998793 | 74 | 0.94 | Y | 2p13.2 | EMX1 | 5' end |
| 4D23 | chr2:70271107-70271507 | 70271307 | 62 | 0.78 | Y | 2p13.3 | FLJ20558 | 5' end |
| 4D26 | chr16:48446206-48446606 | 48446406 | 73 | 0.83 | Y | 16q12.1 | THC2063522 | 5' end |
| 4D29 | chr1:115985850-115986250 | 115986050 | 75 | 0.97 | Y | 1p13.1 | VANGL1 | 5' end |
| 4D31 | chr3:50629407-50629807 | 50629607 | 74 | 0.75 | Y | 3p21.31 | MAPKAPK3 | 5' end |
| 4D32 | chr12:64422062-64422462 | 64422262 | 70 | 0.97 | Y | 12q14.3 | BC001368 | Body |
| 4D33 | chr12:55148348-55148748 | 55148548 | -- | -- | N | 12q13.3 | SPRYD4 | 5' end |
| 4D34 | chr4:1394701-1395101 | 1394901 | 58 | 0.60 | Y | 4p16.3 | NA | Intergenic |
| 4D39 | chr4:171183820-171184220 | 171184020 | 79 | 0.98 | Y | 4q33 | MFAP3L | 5' end |
| 4D45 | chr22:37431977-37432377 | 37432177 | 77 | 0.94 | Y | 22q13.1 | GTPBP1 | 5' end |
| 4D47 | chr5:2801549-2801949 | 2801749 | 76 | 0.91 | Y | 5p15.33 | IRX2 | 5' end |
| 4D50 | chr16:31133540-31133940 | 31133740 | 74 | 0.77 | Y | 16p11.2 | PYC1 | Body |
| 4E01 | chr14:102638235-102638635 | 102638435 | 73 | 1.15 | Y | 14q32.32 | C14orf73 | 5' end |
| 4E02 | chr15:43601976-43602376 | 43602176 | 75 | 1.01 | Y | 15q21.1 | SLC30A4 | 5' end |
| 4E03 | chr9:95254516-95254916 | 95254716 | 63 | 0.73 | Y | 9q22.31 | C9orf10 | 5' end |
| 4E04 | chr15:67493807-67494207 | 67494007 | 76 | 0.87 | Y | 15q23 | KIF23 | Body |
| 4E05 | chr6:43247051-43247451 | 43247251 | 78 | 0.85 | Y | 6p21.1 | SRF | 5' end |
| 4E07 | chr1:161058702-161059102 | 161058902 | 60 | 0.83 | Y | 1q23.3 | THC2117393 | 5' end |
| 4E08 | chr7:95789001-95789401 | 95789201 | 73 | 0.82 | Y | 7q21.3 | SLC25A13 | 5' end |
| 4E11 | chr14:60817427-60817827 | 60817627 | 72 | 0.83 | Y | 14q23.1 | AL080078 | 5' end |
| 4E12 | chr3:195341994-195342394 | 195342194 | 80 | 0.77 | Y | 3q29 | NA | Intergenic |
| 4E14 | chr5:140996386-140996786 | 140996586 | 69 | 0.77 | Y | 5q31.3 | HDAC3 | 5' end |
| 4E15 | chr5:131774450-131774850 | 131774650 | 71 | 0.82 | Y | 5q23.3 | AK128882 | Body |
| 4E16 | chr4:174326770-174327170 | 174326970 | 82 | 0.97 | Y | 4q34.1 | GALNT7 | Body |
| 4E17 | chr15:78483554-78483954 | 78483754 | 78 | 0.94 | Y | 15q25.1 | BC051335 | 5' end |
| 4E18 | chr7:97748870-97749270 | 97749070 | 81 | 0.99 | Y | 7q21.3 | BRI3 | 5' end |
| 4E19 | chr17:37941873-37942273 | 37942073 | 78 | 0.95 | Y | 17q21.2 | NAGLU | 5' end |
| 4E20 | chr12:67366647-67367047 | 67366847 | 58 | 0.81 | Y | 12q15 | NUP107 | 5' end |
| 4E21 | chr12:22669179-22669579 | 22669379 | 70 | 0.90 | Y | 12p12.1 | ETNK1 | 5' end |
| 4E22 | chr7:92300444-92300844 | 92300644 | 72 | 1.09 | Y | 7q21.2 | CDK6 | 5' end |
| 4E24 | chr4:85637773-85638173 | 85637973 | 76 | 0.87 | Y | 4q21.23 | NKX6-1 | 5' end |
| 4E25 | chr9:10602741-10603141 | 10602941 | 74 | 0.89 | Y | 9p23 | PTPRD | 5' end |
| 4E34 | chr2:70870638-70871038 | 70870838 | 65 | 0.69 | Y | 2p13.3 | FIGLA | Body |
| 4E35 | chr20:2031520-2031920 | 2031720 | 72 | 1.03 | Y | 20p13 | STK35 | 5' end |
| 4E36 | chr1:210672738-210673138 | 210672938 | 65 | 0.89 | Y | 1q32.3 | NENF | 5' end |
| 4E37 | chr13:95540497-95540897 | 95540697 | 56 | 1.00 | Y | 13q32.1 | HS6ST3 | 5' end |
| 4E38 | chr11:66251424-66251824 | 66251624 | 67 | 0.75 | Y | 11q13.2 | AF079569 | Body |
| 4E39 | chr19:59386629-59387029 | 59386829 | 71 | 0.90 | Y | 19q13.42 | LENG5 | 5' end |
| 4E41 | chr14:103098722-103099122 | 103098922 | 77 | 1.11 | Y | 14q32.32 | BAG5 | 5' end |
| 4E44 | chr15:38520878-38521278 | 38521078 | 59 | 1.14 | Y | 15q15.1 | BAHD1 | 5' end |
| 4E45 | chr15:50191855-50192255 | 50192055 | 70 | 0.88 | Y | 15q21.2 | BCL2L10 | 5' end |
| 4E48 | chr14:89237535-89237935 | 89237735 | 77 | 0.88 | Y | 14q32.11 | NA | Intergenic |
| 4E49 | chr1:16212010-16212410 | 16212210 | 70 | 0.60 | Y | 1p36.13 | SPEN | Body |
| 4E50 | chr15:83326046-83326446 | 83326246 | 74 | 1.11 | Y | 15q25.3 | PDE8A | 5' end |
| 4E51 | chr22:29420737-29421137 | 29420937 | 78 | 1.05 | Y | 22q12.2 | OSBP2 | 5' end |
| 4E52 | chr8:144838569-144838969 | 144838769 | 63 | 0.79 | Y | 8q24.3 | LOC286075 | 5' end |
| 4E53 | chr13:27450721-27451121 | 27450921 | 58 | 0.73 | Y | 13q12.2 | NA | Intergenic |
| 4E55 | chr5:133589475-133589875 | 133589675 | 72 | 1.02 | Y | 5q31.1 | PPP2CA | 5' end |
| 4E56 | chr21:35183160-35183560 | 35183360 | 74 | 0.94 | Y | 21q22.12 | RUNX1 | 5' end |
| 4F02 | chr10:21863645-21864045 | 21863845 | 74 | 1.01 | Y | 10p12.31 | MLLT10 | Body |
| 4F03 | chr11:129125845-129126245 | 129126045 | 64 | 0.83 | Y | 11q24.3 | NA | Intergenic |
| 4F07 | chr19:38409442-38409842 | 38409642 | 76 | 0.78 | Y | 19q13.11 | SLC7A10 | 5' end |
| 4F08 | chr20:21029994-21030394 | 21030194 | 65 | 0.71 | Y | 20p11.23 | gleeto.cNov04 | 5' end |
| 4F11 | chr15:70197045-70197445 | 70197245 | 73 | 0.80 | Y | 15q23 | SENP8; MYO9A | 5' end |
| 4F15 | chr10:48058377-48058777 | 48058577 | 72 | 0.66 | Y | 10q11.22 | GDF10 | 5' end |
| 4F17 | chr1:44656027-44656427 | 44656227 | 71 | 0.90 | Y | 1p34.1 | AK056424 | Body |
| 4F20 | chr9:5822758-5823158 | 5822958 | 74 | 1.06 | Y | 9p24.1 | AK093217 | 5' end |
| 4F22 | chr3:9570136-9570536 | 9570336 | 78 | 0.88 | Y | 3p25.3 | LOC375323 | 5' end |
| 4F24 | chr3:17758970-17759370 | 17759170 | 71 | 1.02 | Y | 3p24.3 | TBC1D5 | 5' end |
| 4F26 | chr8:125809779-125810179 | 125809979 | 77 | 1.06 | Y | 8q24.13 | MTSS1 | 5' end |
| 4F30 | chr16:2767231-2767631 | 2767431 | 56 | 1.23 | Y | 16p13.3 | TCEB2 | 5' end |
| 4F31 | chr3:197923635-197924035 | 197923835 | 78 | 0.97 | Y | 3q29 | FLJ20522 | 5' end |
| 4F32 | chr10:131655171-131655571 | 131655371 | 71 | 0.79 | Y | 10q26.3 | AK097335 | 5' end |
| 4F35 | chr10:106004354-106004754 | 106004554 | 63 | 0.67 | Y | 10q25.1 | GSTO1 | 5' end |
| 4F36 | chr1:44655757-44656157 | 44655957 | 67 | 0.82 | Y | 1p34.1 | AK056424 | Body |
| 4F37 | chr1:33494607-33495007 | 33494807 | 83 | 0.98 | Y | 1p35.1 | FLJ25476 | 5' end |
| 4F42 | chr1:229623673-229624073 | 229623873 | 76 | 0.94 | Y | 1q42.2 | EGLN1 | 5' end |
| 4F43 | chr4:113372293-113372693 | 113372493 | 68 | 0.82 | Y | 4q25 | BX647702 | 5' end |
| 4F46 | chr6:166675887-166676287 | 166676087 | 68 | 0.87 | Y | 6q27 | C6orf83 | 5' end |
| 4F47 | chr18:45341699-45342099 | 45341899 | 62 | 0.76 | Y | 18q21.1 | LIPG | 5' end |
| 4F48 | chr7:94374815-94375215 | 94375015 | 75 | 0.90 | Y | 7q21.3 | PPP1R9A | 5' end |
| 4F51 | chr6:138524614-138525014 | 138524814 | 75 | 0.87 | Y | 6q23.3 | PBOV1 | Body |
| 4F52 | chr1:176778514-176778914 | 176778714 | 71 | 0.70 | Y | 1q25.2 | AK092849 | 5' end |
| 4F54 | chr4:84249569-84249969 | 84249769 | 63 | 0.72 | Y | 4q21.22 | PLAC8 | 5' end |
| 4F57 | chr2:233777759-233778159 | 233777959 | 70 | 0.74 | Y | 2q37.1 | INPP5D | 5' end |
| 4F58 | chr12:73887531-73887931 | 73887731 | 71 | 0.98 | Y | 12q21.1 | KCNC2 | Body |
| 4F62 | chr2:15649494-15649894 | 15649694 | 67 | 0.75 | Y | 2p24.3 | DDX1 | Body |
| 4F67 | chr17:45283569-45283969 | 45283769 | 72 | 0.96 | Y | 17q21.33 | TAC4 | 5' end |
| 4F69 | chr10:22669345-22669745 | 22669545 | 73 | 0.89 | Y | 10p12.2 | SPAG6 | 5' end |
| 4G21 | chr12:48712830-48713230 | 48713030 | 69 | 0.77 | Y | 12q13.12 | NA | Intergenic |
| 4G53 | chr1:50659481-50659881 | 50659681 | 71 | 1.00 | Y | 1p32.3 | DMRTA2 | 5' end |
| 4G57 | chr10:112317727-112318127 | 112317927 | 72 | 0.59 | N | 10q25.2 | CSPG6 | Body |
| 4G77 | chr4:17122681-17123081 | 17122881 | 77 | 0.94 | Y | 4p15.32 | QDPR | 5' end |
| 4G84 | chr13:20532565-20532965 | 20532765 | 78 | 1.02 | Y | 13q12.11 | LATS2 | 5' end |
| 4G88_89 | chr18:46748167-46748567 | 46748367 | 71 | 0.91 | Y | 18q21.1 | ELAC1 | 5' end |
| 4G90 | chr14:34168460-34168860 | 34168660 | 68 | 0.78 | Y | 14q13.1 | SNX6 | 5' end |
| 4G91 | chr9:134454755-134455155 | 134454955 | 73 | 0.72 | Y | 9q34.13 | BARHL1 | Body |
| 4G99 | chr5:178219361-178219761 | 178219561 | 66 | 0.76 | Y | 5q35.3 | ZNF354B | 5' end |
| 5A05 | chr17:27795427-27795827 | 27795627 | 72 | 0.93 | Y | 17q11.2 | PSMD11 | 5' end |
| 5A08 | chr8:56848397-56848797 | 56848597 | 66 | 0.97 | Y | 8q12.1 | NCOA6IP | 5' end |
| 5A12 | chr6:7671203-7671603 | 7671403 | 75 | 0.76 | Y | 6p24.3 | BMP6 | 5' end |
| 5A20 | chr19:40273556-40273956 | 40273756 | 65 | 0.71 | Y | 19q13.12 | AK124779 | Body |
| 5A21 | chr21:14017845-14018245 | 14018045 | 76 | 0.62 | Y | 21q11.2 | publor | 5' end |
| 5B02 | chr3:51964883-51965283 | 51965083 | 79 | 0.89 | Y | 3p21.2 | GPR62 | 5' end |
| 5B03 | chr3:198766702-198767102 | 198766902 | 65 | 0.93 | Y | 3q29 | BDH | 5' end |
| 5B04 | chr6:91063383-91063783 | 91063583 | 75 | 0.87 | Y | 6q15 | BACH2 | 5' end |
| 5B05 | chr3:31997675-31998075 | 31997875 | 71 | 0.79 | Y | 3p24.1 | OSBPL10 | 5' end |
| 5B07 | chr7:150604924-150605324 | 150605124 | 76 | 0.81 | Y | 7q36.1 | SMARCD3 | 5' end |
| 5B08 | chr2:238814109-238814509 | 238814309 | 73 | 0.84 | Y | 2q37.3 | HES6 | 5' end |
| 5B09 | chr20:3696219-3696619 | 3696419 | 74 | 0.83 | Y | 20p13 | C20orf27 | 5' end |
| 5B11 | chr18:29411959-29412359 | 29412159 | 76 | 1.03 | Y | 18q12.1 | BX648108 | 5' end |
| 5B12 | chr12:12605765-12606165 | 12605965 | 70 | 0.94 | Y | 12p13.2 | DUSP16 | 5' end |
| 5B14 | chr2:104641903-104642303 | 104642103 | 64 | 0.88 | Y | 2q12.1 | NA | Intergenic |
| 5B15 | chr2:111594625-111595025 | 111594825 | 75 | 0.97 | Y | 2q13 | BCL2L11 | 5' end |
| 5B16 | chr9:138497872-138498272 | 138498072 | 67 | 1.00 | Y | 9q34.3 | FLJ36779 | 5' end |
| 5B17 | chr4:113663612-113664012 | 113663812 | 56 | 0.89 | Y | 4q25 | bloygaw.b | Body |
| 5B18 | chr21:33773415-33773815 | 33773615 | 65 | 0.97 | Y | 21q22.11 | C21orf4 | 5' end |
| 5B19 | chr1:8406140-8406540 | 8406340 | 80 | 0.83 | Y | 1p36.23 | RERE | Body |
| 5B20 | chr13:94417783-94418183 | 94417983 | 65 | 0.79 | Y | 13q32.1 | THC2102987 | 5' end |
| 5B22 | chr12:51371089-51371489 | 51371289 | 65 | 0.87 | Y | 12q13.13 | KRT1B | Body |
| 5B27 | chr2:232924116-232924516 | 232924316 | 71 | 0.73 | Y | 2q37.1 | NA | Intergenic |
| 5B30 | chr2:43307653-43308053 | 43307853 | 69 | 0.82 | Y | 2p21 | ZFP36L2 | 5' end |
| 5B34 | chr8:17398828-17399228 | 17399028 | 81 | 0.87 | Y | 8p22 | SLC7A2, | 5' end |
| 5B35 | chr16:32159872-32160272 | 32160072 | 54 | 0.71 | Y | 16p11.2 | THC2215073_4 | 5' end |
| 5B37 | chr1:210849253-210849653 | 210849453 | 72 | 0.81 | Y | 1q32.3 | ATF3 | 5' end |
| 5C01 | chr22:19388335-19388735 | 19388535 | 76 | 0.92 | Y | 22q11.21 | DKFZp434N035 | Body |
| 5C02 | chr17:25280867-25281267 | 25281067 | 62 | 1.13 | Y | 17q11.2 | SSH2 | 5' end |
| 5C03 | chr5:179218729-179219129 | 179218929 | 64 | 0.88 | Y | 5q35.3 | BC050714 | 5' end |
| 5C04 | chr2:67477752-67478152 | 67477952 | 59 | 0.82 | Y | 2p14 | ETAA16 | 5' end |
| 5C06 | chr14:54947867-54948267 | 54948067 | 67 | 0.85 | Y | 14q22.3 | BC028015 | Body |
| 5C08 | chr11:63524334-63524734 | 63524534 | 76 | 0.78 | Y | 11q13.1 | LRP16 | Body |
| 5C11 | chr15:89338218-89338618 | 89338418 | 67 | 0.73 | Y | 1q41 | RAB3-GAP150 | 5' end |
| 5C11 | chr15:89338218-89338618 | 89338418 | 76 | 0.95 | Y | 15q26.1 | PRC1 | Body |
| 5C15 | chr1:183552370-183552770 | 183552570 | 64 | 1.01 | Y | 1q25.3 | IVNS1ABP | 5' end |
| 5C17 | chr9:72224869-72225269 | 72225069 | 66 | 0.92 | Y | 9q21.11 | THC2062998 | 5' end |
| 5C18 | chr5:178352801-178353201 | 178353001 | 76 | 1.06 | Y | 5q35.3 | GRM6 | Body |
| 5C20 | chr2:219574607-219575007 | 219574807 | 71 | 0.85 | Y | 2q35 | AK092605 | 5' end |
| 5C22 | chr6:150353555-150353955 | 150353755 | 63 | 0.85 | Y | 6q25.1 | THC2078290 | 5' end |
| 5C23 | chr18:51140645-51141045 | 51140845 | 59 | 0.74 | Y | 18q21.2 | TCF4 | Body |
| 5C24 | chr19:10202749-10203149 | 10202949 | 76 | 0.75 | Y | 19p13.2 | EDG5 | 5' end |
| 5C25 | chr6:105734134-105734534 | 105734334 | 73 | 0.96 | Y | 6q21 | POPDC3 | 5' end |
| 5C27 | chr13:40137854-40138254 | 40138054 | 67 | 0.98 | Y | 13q14.11 | FOXO1A | 5' end |
| 5C28 | chr15:65963990-65964390 | 65964190 | 69 | 0.80 | Y | 15q23 | NA | Intergenic |
| 5C29 | chr4:10067712-10068112 | 10067912 | 79 | 0.97 | Y | 4p16.1 | BC040874 | 5' end |
| 5C30 | chr5:92941524-92941924 | 92941724 | 67 | 0.89 | Y | 5q15 | NR2F1 | 5' end |
| 5C31 | chr20:2974588-2974988 | 2974788 | 74 | 0.95 | Y | 20p13 | MRPS26 | 5' end |
| 5C32 | chr1:206150742-206151142 | 206150942 | 71 | 0.79 | Y | 1q32.2 | CD34 | Body |
| 5C34 | chr17:52024852-52025252 | 52025052 | 68 | 0.77 | Y | 17q23.2 | NOG | 5' end |
| 5C35 | chr1:77520093-77520493 | 77520293 | 77 | 0.88 | Y | 1p31.1 | AK5 | 5' end |
| 5C36 | chr5:177949426-177949826 | 177949626 | 79 | 1.05 | Y | 5q35.3 | COL23A1 | 5' end |
| 5C37 | chr6:144370630-144371030 | 144370830 | 75 | 1.02 | Y | 6q24.2 | PLAGL1 | 5' end |
| 5C38 | chr16:67038296-67038696 | 67038496 | 71 | 0.87 | Y | 16q22.1 | SMPD3 | 5' end |
| 5C39 | chr19:52803912-52804312 | 52804112 | 61 | 1.01 | Y | 19q13.33 | GLTSCR1 | Body |
| 5C40 | chr6:53624786-53625186 | 53624986 | 62 | 0.98 | Y | 6p12.1 | KLHL | Body |
| 5C41 | chr12:51727435-51727835 | 51727635 | 78 | 0.90 | Y | 12q13.13 | TENC1 | 5' end |
| 5C42 | chr10:118754219-118754619 | 118754419 | 68 | 0.89 | Y | 10q26.11 | AB046818 | Body |
| 5D01 | chr13:31503461-31503861 | 31503661 | 67 | 0.78 | Y | 13q13.1 | 13CDNA73 | 5' end |
| 5D02 | chr1:68071132-68071532 | 68071332 | 72 | 0.71 | Y | 1p31.2 | GNG12 | 5' end |
| 5D03 | chr3:196462033-196462433 | 196462233 | 75 | 0.90 | Y | 3q29 | FLJ35155 | Body |
| 5D04 | chr1:242278446-242278846 | 242278646 | 80 | 0.70 | Y | 1q44 | ZNF238 | 5' end |
| 5D06 | chr15:47125455-47125855 | 47125655 | 71 | 0.72 | Y | 15q21.1 | KIAA0256 | Body |
| 5D07 | chr10:129898599-129898999 | 129898799 | 54 | 0.69 | Y | 10q26.2 | AK124226 | Body |
| 5D08 | chr18:31210950-31211350 | 31211150 | 73 | 0.83 | Y | 18q12.2 | ZNF396 | 5' end |
| 5D09 | chr2:233500069-233500469 | 233500269 | 70 | 0.75 | Y | 2q37.1 | NGEF | Body |
| 5D11 | chr1:67291264-67291664 | 67291464 | 48 | 0.73 | N | 1p31.2 | SLC35D1 | Body |
| 5D12 | chr2:97978246-97978646 | 97978446 | 79 | 0.99 | Y | 2q11.2 | AF119842 | 5' end |
| 5D13 | chr9:128427836-128428236 | 128428036 | 66 | 0.83 | Y | 9q33.3 | LMX1B | Body |
| 5D15 | chr2:9480488-9480888 | 9480688 | 72 | 0.81 | Y | 2p25.1 | CPSF3; ITGB1BP1 | 5' end |
| 5D16 | chr5:170671197-170671597 | 170671397 | 70 | 0.91 | Y | 5q35.1 | TLX3 | Body |
| 5D17 | chr11:13255538-13255938 | 13255738 | 70 | 0.89 | Y | 11p15.3 | ARNTL | 5' end |
| 5D19 | chr1:1811120-1811520 | 1811320 | 67 | 0.94 | Y | 1p36.33 | GNB1 | 5' end |
| 5D25 | chr19:15480230-15480630 | 15480430 | 75 | 0.95 | Y | 19p13.12 | FLJ39501 | 5' end |
| 5D29 | chr5:121675463-121675863 | 121675663 | 81 | 0.96 | Y | 5q23.2 | SNCAIP | 5' end |
| 5D30 | chr4:107036107-107036507 | 107036307 | 69 | 0.84 | Y | 4q24 | LOC255743 | 5' end |
| 5D31 | chr4:37131725-37132125 | 37131925 | 73 | 1.03 | Y | 4p14 | BC037906 | 5' end |
| 5D32 | chr10:126595308-126595708 | 126595508 | 76 | 0.88 | Y | 10q26.13 | ZRANB1 | 5' end |
| 5D34 | chr7:50828106-50828506 | 50828306 | 83 | 1.05 | Y | 7p12.2 | GRB10 | 5' end |
| 5D39 | chr4:152549714-152550114 | 152549914 | 68 | 0.76 | Y | 4q31.3 | THC2176498 | 5' end |
| 5E01 | chr11:19690676-19691076 | 19690876 | 74 | 1.00 | Y | 11p15.1 | NAV2 | 5' end |
| 5E02 | chr16:85982647-85983047 | 85982847 | 69 | 0.81 | Y | 16q24.2 | MAP1LC3B | 5' end |
| 5E03 | chr6:7535462-7535862 | 7535662 | 71 | 0.84 | Y | 6p24.3 | C6orf151 | Body |
| 5E04 | chr2:98805454-98805854 | 98805654 | 78 | 0.97 | Y | 2q11.2 | MGC42367 | Body |
| 5E05 | chr8:31010134-31010534 | 31010334 | 78 | 0.83 | Y | 8p12 | WRN;PURG | 5' end |
| 5E06 | chr3:184454142-184454542 | 184454342 | 68 | 1.00 | Y | 3q27.1 | B3GNT5 | 5' end |
| 5E07 | chr2:228045093-228045493 | 228045293 | 75 | 1.00 | Y | 2q36.3 | HRB | 5' end |
| 5E08 | chr15:60243387-60243787 | 60243587 | 74 | 0.9 | Y | 15q22.2 | NLF2 | Body |
| 5E09 | chr2:130846413-130846813 | 130846613 | 74 | 0.88 | Y | 2q21.1 | PTPN18 | Body |
| 5E11 | chr18:55515418-55515818 | 55515618 | 69 | 0.67 | Y | 18q21.32 | CCBE1 | 5' end |
| 5E13 | chr15:73533729-73534129 | 73533929 | 74 | 0.95 | Y | 15q24.2 | SIN3A | 5' end |
| 5E14 | chr11:101644441-101644841 | 101644641 | 65 | 0.57 | N | 11q22.1 | NA | Intergenic |
| 5E15 | chr22:45071775-45072175 | 45071975 | 58 | 0.58 | N | 22q13.31 | GTSE1 | 5' end |
| 5E16 | chr9:132578069-132578469 | 132578269 | 73 | 0.85 | Y | 9q34.12 | ABL1 | 5' end |
| 5E18 | chr20:25318829-25319229 | 25319029 | 77 | 0.79 | Y | 20p11.21 | C20orf22 | Body |
| 5E20 | chr15:43601664-43602064 | 43601864 | 63 | 1.08 | Y | 15q21.1 | SLC30A4 | 5' end |
| 5E22 | chr12:50603467-50603867 | 50603667 | 65 | 0.56 | N | 12q13.13 | THC2089122 | Body |
| 5E25 | chr1:238323016-238323416 | 238323216 | 70 | 0.60 | Y | 1q43 | FMN2 | 5' end |
| 5E27 | chr7:21549376-21549776 | 21549576 | 66 | 0.81 | Y | 7p15.3 | DNAH11 | 5' end |
| 5E28 | chr19:50374567-50374967 | 50374767 | 77 | 1.00 | Y | 19q13.32 | BLOC1S3 | 5' end |
| 5E31 | chr12:46499598-46499998 | 46499798 | 81 | 0.77 | Y | 12q13.11 | HDAC7A | Body |
| 5E32 | chr17:44046380-44046780 | 44046580 | 66 | 0.87 | Y | 17q21.32 | HOXB8 | 5' end |
| 5E33 | chr21:37005479-37005879 | 37005679 | 70 | 0.7 | Y | 21q22.13 | SIM2 | Body |
| 5E34 | chr11:32417065-32417465 | 32417265 | 64 | 0.87 | Y | 11p13 | WIT-1 | 5' end |
| 5E35 | chr5:114908255-114908655 | 114908455 | 72 | 0.92 | Y | 5q22.3 | FEM1C | 5' end |
| 5E36 | chr2:219751072-219751472 | 219751272 | 75 | 0.90 | Y | 2q35 | MGC3035 | 5' end |
| 5E40 | chr6:34468071-34468471 | 34468271 | 77 | 1.03 | Y | 6p21.31 | NUDT3 | 5' end |
| 5E46 | chr1:92123606-92124006 | 92123806 | 73 | 0.77 | Y | 1p22.1 | TGFBR3 | 5' end |
| 5E49 | chr1:16174513-16174913 | 16174713 | 71 | 0.90 | Y | 1p36.13 | ZNF151 | 5' end |
| 5F02 | chr10:43389543-43389943 | 43389743 | 71 | 0.91 | Y | 10q11.21 | ZNF239 | Body |
| 5F03 | chr4:119173791-119174191 | 119173991 | 71 | 0.82 | Y | 4q26 | NDST3 | 5' end |
| 5F05 | chr17:77280746-77281146 | 77280946 | 73 | 0.90 | Y | 17q25.3 | MRPL12 | 5' end |
| 5F07 | chr2:23461083-23461483 | 23461283 | 73 | 0.86 | Y | 2p24.1 | KBTBD9 | 5' end |
| 5F13 | chr18:71296405-71296805 | 71296605 | 74 | 0.96 | Y | 18q22.3 | bloploy | 5' end |
| 5F21 | chr2:185171136-185171536 | 185171336 | 67 | 0.90 | Y | 2q32.1 | C2orf10 | 5' end |
| 5F26 | chr15:56144819-56145219 | 56145019 | 69 | 0.93 | Y | 15q21.3 | ALDH1A2 | Body |
| 5F28 | chr10:88270885-88271285 | 88271085 | 66 | 0.77 | Y | 10q23.2 | KIAA0261 | 5' end |
| 5F32 | chr10:118598884-118599284 | 118599084 | 61 | 0.74 | Y | 10q25.3 | BX647301 | 5' end |
| 5F35 | chr10:132999979-133000379 | 133000179 | 83 | 0.87 | Y | 10q26.3 | TCERG1L | 5' end |
| 5F37 | chr3:148588517-148588917 | 148588717 | 51 | 0.88 | Y | 3q24 | ZIC4 | Body |
| 5F38 | chr22:48966214-48966614 | 48966414 | 80 | 0.98 | Y | 22q13.33 | PP2447 | 5' end |
| 5F46 | chr5:90711486-90711886 | 90711686 | 62 | 1.21 | Y | 5q14.3 | ARRDC3 | Body |
| 5F51 | chr7:26382650-26383050 | 26382850 | 68 | 0.81 | Y | 7p15.2 | THC2052841 | 5' end |
| 5F57 | chr16:66613909-66614309 | 66614109 | 65 | 0.81 | Y | 16q22.1 | DDX28 | 5' end |
| 5F58 | chr2:39517570-39517970 | 39517770 | 74 | 0.95 | Y | 2p22.1 | MAP4K3 | 5' end |
| 5F60 | chr6:150226743-150227143 | 150226943 | 79 | 0.95 | Y | 6q25.1 | LRP11 | 5' end |
| 5G03 | chr3:142940033-142940433 | 142940233 | 64 | 0.77 | Y | 3q23 | RNF7 | Body |
| 5G08 | chr4:108965441-108965841 | 108965641 | 75 | 0.96 | Y | 4q25 | AF452717 | Body |
| 5G18 | chr11:59279315-59279715 | 59279515 | 77 | 0.95 | Y | 11q12.1 | STX3A | 5' end |
| 5G20 | chr5:132327253-132327653 | 132327453 | 73 | 0.74 | Y | 5q31.1 | AF5Q31 | 5' end |
| 5G28 | chr2:96556892-96557292 | 96557092 | 70 | 0.61 | Y | 2q11.2 | chodoy | 5' end |
| 5G29 | chr20:21054913-21055313 | 21055113 | 60 | 0.59 | N | 20p11.23 | C20orf19 | Body |
| 5G35 | chr8:66916297-66916697 | 66916497 | 76 | 0.91 | Y | 8q13.1 | PDE7A | 5' end |
| 5G48 | chr11:11820183-11820583 | 11820383 | 70 | 0.76 | Y | 11p15.3 | USP47 | Body |
| 5G64 | chr22:25209670-25210070 | 25209870 | 74 | 1.01 | Y | 22q12.1 | HPS4 | 5' end |
| 6B06 | chr1:47469090-47469490 | 47469290 | 66 | 0.84 | Y | 1p33 | TAL1 | 5' end |
| 6B08 | chr12:63801434-63801834 | 63801634 | 73 | 0.82 | Y | 12q14.3 | WIF1 | 5' end |
| 6B13 | chr1:63562519-63562919 | 63562719 | 70 | 0.87 | Y | 1p31.3 | FOXD3 | 5' end |
| 6B16 | chr8:132123575-132123975 | 132123775 | 68 | 0.76 | Y | 8q24.22 | ADCY8 | 5' end |
| 6B17 | chr2:105312867-105313267 | 105313067 | 64 | 0.68 | Y | 2q12.1 | TGFBRAP1 | 5' end |
| 6C02 | chr5:131859343-131859743 | 131859543 | 68 | 0.58 | N | 5q23.3 | SLC22A4.1 | 5' end |
| 6C04 | chr19:44689497-44689897 | 44689697 | 77 | 1.06 | Y | 19q13.2 | DLL3 | Body |
| 6C06 | chr8:119702535-119702935 | 119702735 | 61 | 0.48 | N | 8q24.12 | SAMD12 | Body |
| 6C10 | chr4:103484596-103484996 | 103484796 | 73 | 0.85 | Y | 4q24 | SLC39A8 | 5' end |
| 6C12 | chr7:50828591-50828991 | 50828791 | 79 | 0.92 | Y | 7p12.2 | GRB10 | 5' end |
| 6C14 | chr4:152548552-152548952 | 152548752 | 58 | 0.64 | Y | 4q31.3 | THC2096148 | Body |
| 6C21 | chr14:59407123-59407523 | 59407323 | 67 | 0.79 | Y | 14q23.1 | RTN1 | 5' end |
| 6D03 | chr12:30739976-30740376 | 30740176 | 61 | 0.86 | Y | 12p11.21 | IPO8 | 5' end |
| 6D04 | chr11:86060919-86061319 | 86061119 | 65 | 0.76 | Y | 11q14.2 | ME3 | 5' end |
| 6D06 | chr10:75581251-75581651 | 75581451 | 64 | 0.70 | Y | 10q22.2 | ADK | 5' end |
| 6D08 | chr18:55177408-55177808 | 55177608 | 71 | 0.76 | Y | 18q21.32 | LMAN1 | 5' end |
| 6D10 | chr2:19413601-19414001 | 19413801 | 65 | 0.67 | Y | 2p24.1 | NA | Intergenic |
| 6D11 | chr9:22437015-22437415 | 22437215 | 71 | 0.87 | Y | 9p21.3 | DMRTA1 | 5' end |
| 6D12 | chr13:78073998-78074398 | 78074198 | 84 | 1.06 | Y | 13q31.1 | POU4F1 | Body |
| 6D14 | chr10:63804031-63804431 | 63804231 | 74 | 0.85 | Y | 10q21.2 | ZNF365 | 5' end |
| 6D15 | chr18:45593711-45594111 | 45593911 | 70 | 0.76 | Y | 18q21.1 | ACAA2 | 5' end |
| 6D16 | chr3:23823425-23823825 | 23823625 | 61 | 0.62 | Y | 3p24.2 | UBE2E1 | 5' end |
| 6D17 | chr5:126141273-126141673 | 126141473 | 68 | 1.03 | Y | 5q23.2 | LMNB1 | Body |
| 6D20 | chr9:135009581-135009981 | 135009781 | 70 | 0.76 | Y | 9q34.2 | RALGDS | Body |
| 6D22 | chr22:41582853-41583253 | 41583053 | 75 | 0.89 | Y | 22q13.2 | ARFGAP3 | Body |
| 6D24 | chr14:29466730-29467130 | 29466930 | 80 | 0.89 | Y | 14q12 | PRKCM | 5' end |
| 6D25 | chr18:43028565-43028965 | 43028765 | 73 | 0.95 | Y | 18q21.1 | AF119875 | Body |
| 6D28 | chr11:67130971-67131371 | 67131171 | 67 | 0.78 | Y | 11q13.2 | NDUFV1 | 5' end |
| 6E08 | chr13:41512356-41512756 | 41512556 | 77 | 0.92 | Y | 13q14.11 | NA | Intergenic |
| 6E14 | chr13:96884509-96884909 | 96884709 | 75 | 1.07 | Y | 13q32.1 | RAP2A | 5' end |
| 6F03 | chr1:155281885-155282285 | 155282085 | 75 | 1.05 | Y | 1q23.1 | ARHGEF11 | 5' end |
| 6F05 | chr7:140420098-140420498 | 140420298 | 74 | 0.79 | Y | 7q34 | THC2186277 | 5' end |
| 6F07 | chr6:30632752-30633152 | 30632952 | 57 | 0.71 | Y | 6p21.33 | GNL1 | 5' end |
| 6F09 | chr5:150807104-150807504 | 150807304 | 63 | 0.59 | N | 5q33.1 | SLC36A1 | 5' end |
| 6F10 | chr6:143309232-143309632 | 143309432 | 62 | 0.87 | Y | 6q24.2 | HIVEP2 | 5' end |
| 6F17 | chr16:2894901-2895301 | 2895101 | 71 | 0.78 | Y | 16p13.3 | LOC440333 | 5' end |
| 6G02 | chr1:88922351-88922751 | 88922551 | 69 | 0.95 | Y | 1p22.2 | PRKCL2 | 5' end |
| 6G35 | chr15:57067453-57067853 | 57067653 | 61 | 0.70 | Y | 15q22.1 | RNF111 | 5' end |
