# Supplementary material for: Restriction Landmark Genomic Scanning (RLGS) spot identification by second generation virtual RLGS in multiple genomes with multiple enzyme combinations
Source: BMC Genomics. 2007 Nov 30;8:446. doi: 10.1186/1471-2164-8-446 (PMC2235865; doi:10.1186/1471-2164-8-446)
Supplement: Additional File 6 — Mouse RLGS spot clones (mm8). Annotation of all cloned mouse RLGS spots from enzyme combination NotI-EcoRV-HinfI. [file 1471-2164-8-446-S6.doc]

Supplementary Table 4 Mouse RLGS spot clones (mm8)

| **Spot** | **NotI site +/- 200bp (Feb '06)** | **NotI site** | **%GC** | **O:E** | **CpG count** | **CpG island** | **Cytogeneitc mapping** | **Gene Homology** | **Context** |
| --- | --- | --- | --- | --- | --- | --- | --- | --- | --- |
| 1C09 | chr16:4502026-4502425 | 4502226 | 61 | 0.61 | 23 | Y | 16qA1 | AK145691 | Body |
| 1C10 | chr19:38901862-38902261 | 38902062 | 76 | 1 | 58 | Y | 19qC3 | Tbc1d12 | 5'end |
| 1C12 | chr4:136628996-136629395 | 136629196 | 78 | 0.82 | 50 | Y | 4qD3 | Cdc42 | 5'end |
| 1C13 | chr16:10308574-10308973 | 10308774 | 63 | 0.63 | 25 | Y | 16qA1 | AK014352 | 5'end |
| 1D01 | chr13:33943320-33943719 | 33943520 | 58 | 0.47 | 16 | N | 13qA3.3 | Serpinb6a | 5'end |
| 1D06 | chr13:48882859-48883258 | 48883059 | 83 | 0.95 | 65 | Y | 13qA5 | Phf2 | 5'end |
| 1D14 | chr3:34841732-34842131 | 34841932 | 73 | 1.02 | 54 | Y | 3qA3 | Sox2 | 5'end |
| 1D16 | chr7:36406688-36407087 | 36406888 | 76 | 0.87 | 50 | Y | 7qB2 | Zfp537 | 5'end |
| 1D20 | chr6:4550876-4551275 | 4551076 | 73 | 0.81 | 43 | Y | 6qA1 | Cast1 | 5'end |
| 1D22 | chr7:109751304-109751703 | 109751504 | 81 | 1.03 | 67 | Y | 7qE3 | Rab6ip1 | 5'end |
| 1D26 | chr8:37717071-37717470 | 37717271 | 78 | 0.99 | 61 | Y | 8qA4 | Lonrf1 | 5'end |
| 1D27 | chr4:130224689-130225088 | 130224889 | 54 | 0.58 | 17 | N | 4qD2.3 | Matn1 | Body |
| 1D28 | chr3:87006127-87006526 | 87006327 | 71 | 1.02 | 52 | Y | 3qF1 | Dcamkl2 | 5'end |
| 1E05 | chr1:193838696-193839095 | 193838896 | 77 | 0.89 | 54 | Y | 1qH6 | Rcor3 | 5'end |
| 1E09 | chr6:49145179-49145578 | 49145379 | 54 | 0.58 | 16 | N | 6qB2.3 | Igf2bp3 | 5'end |
| 1E16 | chr18:83048737-83049136 | 83048937 | 74 | 0.72 | 39 | Y | 18qE4 | CJ103567 | 5'end |
| 1E19 | chr4:13670109-13670508 | 13670309 | 73 | 0.96 | 51 | Y | 4qA1 | AK032132 | 5'end |
| 1E20 | chr8:73013672-73014071 | 73013872 | 63 | 0.83 | 33 | Y | 8qB3.3 | Hapln4 | 5'end |
| 1E22 | chr10:22420535-22420934 | 22420735 | 66 | 0.92 | 40 | Y | 10qA3 | Tbpl1 | 5'end |
| 1E23 | chr18:53598678-53599080 | 53598878 | 66 | 0.74 | 32 | Y | 18qD2 | DQ340564 | 5'end |
| 1E24 | chr16:22180779-22181178 | 22180979 | 69 | 1.08 | 51 | Y | 16qB1 | Sfrs10 | 5'end |
| 1E25 | chr13:114108680-114109079 | 114108880 | 68 | 0.98 | 46 | Y | 13qD2.2 | AK053890 | 5'end |
| 1E27 | chr6:92056641-92057040 | 92056841 | 69 | 1.03 | 49 | Y | 6qD1 | AK157305 | 5'end |
| 1E30 | chr15:102838605-102839004 | 102838805 | 57 | 0.77 | 25 | Y | 15qF3 | Hoxc5 | 5'end |
| 1F02 | chr2:74469312-74469711 | 74469512 | 67 | 1.06 | 48 | Y | 2qC3 | Hoxd13 | 5'end |
| 1F06 | chr9:65258863-65259262 | 65259063 | 68 | 0.87 | 41 | Y | 9qC | Spg21 | 5'end |
| 1F08 | chr5:129334280-129334679 | 129334480 | 69 | 0.96 | 46 | Y | 5qG1.3 | Ran | 5'end |
| 1F11 | chr2:58962568-58962967 | 58962768 | 65 | 0.96 | 41 | Y | 2qC1.1 | Pkp4 | 5'end |
| 1F14 | chr8:12396761-12397160 | 12396961 | 75 | 1.01 | 58 | Y | 8qA1.1 | Sox1 | 5'end |
| 1F15 | chr13:25277302-25277701 | 25277502 | 67 | 0.85 | 37 | Y | 13qA3.1 | Vmp | 5'end |
| 1F21 | chrX:54377906-54378305 | 54378106 | 65 | 0.78 | 32 | Y | XqA5 | Zic3 | 5'end |
| 1F25 | chr6:92056642-92057041 | 92056842 | 69 | 1.03 | 49 | Y | 6qD1 | AK157305 | 5'end |
| 1F27 | chr2:179972233-179972632 | 179972433 | 73 | 0.82 | 43 | Y | 2qH4 | Psma7/Ss18l1 | 5'end |
| 1F30 | chr11:8524470-8524869 | 8524670 | 70 | 0.89 | 44 | Y | 11qA1 | AK089717 | Body |
| 1F33 | chr2:74469112-74469511 | 74469312 | 76 | 1 | 58 | Y | 2qC3 | Hoxd13 | 5'end |
| 1F40 | chr11:102133197-102133596 | 102133397 | 76 | 0.95 | 54 | Y | 11qE1 | Ubtf | 5'end |
| 1G05 | chr15:79260277-79260676 | 79260477 | 53 | 0.63 | 18 | N | 15qE1 | Csnk1e | Body |
| 1G09 | chr3:148927154-148927553 | 148927354 | 70 | 0.82 | 41 | Y | 3qH3 | AK142828 | 5'end |
| 1G11 | chr9:30961077-30961476 | 30961277 | 70 | 1 | 49 | Y | 9qA4 | Aplp2 | 5'end |
| 1G14 | chr7:109494970-109495369 | 109495170 | 65 | 0.67 | 27 | Y | 7qE3 | ICRFP703B1614Q5.6 | 5'end |
| 1G31 | chrX:104018442-104018841 | 104018642 | 60 | 0.49 | 17 | N | XqD | 2610002M06Rik | 5'end |
| 1G32 | chr10:53318045-53318444 | 53318245 | 71 | 1.12 | 57 | Y | 10qB3 | Mcmdc1 | 5'end |
| 1G34 | chr4:59097047-59097446 | 59097247 | 74 | 0.94 | 51 | Y | 4qB3 | AK015285 | 5'end |
| 1G37 | chr13:105041120-105041519 | 105041320 | 78 | 1.07 | 66 | Y | 13qD1 | Erbb2ip | 5'end |
| 1G50 | chr7:80561795-80562194 | 80561995 | 77 | 1.02 | 59 | Y | 7qD2 | Crtc3 | 5'end |
| 1G51 | chr9:49550472-49550871 | 49550672 | 62 | 0.78 | 30 | Y | 9qA5.3 | Ncam1 | 5'end |
| 1H21 | chr14:62561265-62561664 | 62561465 | 70 | 0.64 | 32 | Y | 14qD1 | AY534252 | 5'end |
| 2B02 | chr1:162743215-162743614 | 162743415 | 75 | 0.91 | 49 | Y | 1qH2.1 | Rc3h1 | 5'end |
| 2B07 | chr3:18246769-18247168 | 18246969 | 70 | 0.99 | 49 | Y | 3qA1 | Bhlhb5 | 5'end |
| 2B13 | chr10:25049316-25049715 | 25049516 | 80 | 0.96 | 61 | Y | 10qA4 | AK144088 | 5'end |
| 2B16 | chr7:37727971-37728370 | 37728171 | 75 | 1.12 | 61 | Y | 7qB2 | C80913 | 5'end |
| 2B17 | chr8:95247334-95247733 | 95247534 | 70 | 1.01 | 49 | Y | 8qC5 | Irx5 | 5'end |
| 2B18 | chr18:77063637-77064036 | 77063837 | 69 | 0.81 | 39 | Y | 18qE3 | Intergenic | Intergenic |
| 2B19 | chr2:163173056-163173455 | 163173256 | 66 | 0.41 | 18 | N | 2qH3 | Intergenic | Intergenic |
| 2B21 | chr13:8202197-8202596 | 8202397 | 55 | 0.46 | 14 | N | 13qA1 | Adarb2 | 5'end |
| 2B23 | chr11:43280698-43281097 | 43280898 | 49 | 0.47 | 11 | N | 11qB1.1 | D11Ertd730e | 5'end |
| 2B27 | chr7:114206103-114206502 | 114206303 | 77 | 1.06 | 63 | Y | 7qF1 | Pde3b | 5'end |
| 2B31 | chr10:80885668-80886067 | 80885868 | 68 | 0.8 | 37 | Y | 10qC1 | BC079845 | 5'end |
| 2B32 | chr5:112583164-112583563 | 112583364 | 77 | 1.07 | 64 | Y | 5qF | Hps4 | 5'end |
| 2B33 | chr17:4951276-4951675 | 4951476 | 75 | 0.92 | 52 | Y | 17qA1 | Arid1b | 5'end |
| 2B40 | chr11:118857480-118857879 | 118857680 | 65 | 0.72 | 29 | Y | 11qE2 | Cbx8 | 5'end |
| 2B45 | chr16:18002389-18002788 | 18002589 | 68 | 0.86 | 40 | Y | 16qA3 | Prodh | 5'end |
| 2C06 | chr1:183856245-183856644 | 183856445 | 81 | 1.14 | 76 | Y | 1qH5 | Enah | 5'end |
| 2C07 | chr10:79897779-79898178 | 79897979 | 67 | 0.66 | 30 | Y | 10qC1 | Onecut3 | 5'end |
| 2C08 | chr2:179904764-179905163 | 179904964 | 83 | 0.91 | 63 | Y | 2qH4 | AY038601 | 5'end |
| 2C09 | chr4:99148377-99148776 | 99148577 | 71 | 0.96 | 49 | Y | 4qC6 | Foxd3 | 5'end |
| 2C14 | chrX:96022785-96023184 | 96022985 | 73 | 0.8 | 43 | Y | XqC3 | S59969 | Body |
| 2C15 | chr2:19575607-19576006 | 19575807 | 77 | 1.08 | 63 | Y | 2qA3 | AK076525 | 5'end |
| 2C19 | chr6:85477443-85477842 | 85477643 | 73 | 1.01 | 52 | Y | 6qC3 | Egr4 | 5'end |
| 2C22 | chr13:54747424-54747824 | 54747624 | 72 | 0.97 | 57 | Y | 13qB1 | Gprin1 | 3'end |
| 2C25 | chr5:117618206-117618605 | 117618406 | 80 | 0.96 | 62 | Y | 5qF | Wsb2 | 5'end |
| 2C27 | chr9:61167559-61167958 | 61167759 | 60 | 0.6 | 22 | Y | 9qB | Tle3 | 5'end |
| 2C28 | chr8:72811437-72811836 | 72811637 | 69 | 1.04 | 50 | Y | 8qB3.3 | AK004006 | 5'end |
| 2C29 | chr13:41231148-41231547 | 41231348 | 81 | 1.09 | 71 | Y | 13qA4 | Elovl2 | 5'end |
| 2C31 | chr3:57935393-57935792 | 57935593 | 80 | 1.03 | 65 | Y | 3qD | Pfn2 | 5'end |
| 2C33 | chr13:60186247-60186646 | 60186447 | 74 | 0.99 | 55 | Y | 13qB3 | Gas1 | 5'end |
| 2C34 | chr10:122388364-122388763 | 122388564 | 67 | 1.16 | 52 | Y | 10qD2 | AK052706 | 5'end |
| 2C35 | chr9:85123570-85123969 | 85123770 | 73 | 0.97 | 51 | Y | 9qE3.1 | AK135132 | 5'end |
| 2C36 | chr7:131334125-131334524 | 131334325 | 73 | 0.94 | 47 | Y | 7qF3 | Hmx3 | 5'end |
| 2C37 | chr13:9833600-9834000 | 9,833,800 |  |  |  | N |  | -- | Intergenic |
| 2C39 | chr4:47374231-47374630 | 47374431 | 78 | 0.97 | 56 | Y | 4qB1 | Tgfbr1 | 5'end |
| 2C41 | chr8:73456575-73456974 | 73456775 | 72 | 0.92 | 46 | Y | 8qB3.3 | Fkbp8 | 5'end |
| 2C46 | chr18:80484927-80485326 | 80485127 | 70 | 0.99 | 49 | Y | 18qE3 | AK039137 | 5'end |
| 2C49 | chr3:87006074-87006473 | 87006274 | 69 | 1.04 | 49 | Y | 3qF1 | Dcamkl2 | 5'end |
| 2C51 | chr10:53439336-53439735 | 53439536 | 78 | 1 | 62 | Y | 10qB3 | AK015334 | 5'end |
| 2C52 | chr10:57842336-57842735 | 57842536 | 74 | 1.02 | 56 | Y | 10qB4 | Ranbp2 | 5'end |
| 2C53 | chr9:63725635-63726034 | 63725835 | 67 | 0.59 | 27 | N | 9qC | AA017743 | 5'end |
| 2C55 | chr18:14826011-14826410 | 14826211 | 67 | 0.92 | 42 | Y | 18qA1 | Ss18 | 5'end |
| 2D03 | chr3:116243386-116243785 | 116243586 | 67 | 0.66 | 30 | Y | 3qG1 | Gpr88 | 3'end |
| 2D06 | chr3:68581885-68582284 | 68582085 | 67 | 0.75 | 34 | Y | 3qE1 | AK146930 | 5'end |
| 2D07 | chr9:49550465-49550864 | 49550665 | 61 | 0.77 | 29 | Y | 9qA5.3 | Ncam1 | Body |
| 2D17 | chr13:48274703-48275102 | 48274903 | 52 | 0.58 | 16 | N | 13qA5 | Id4 | 3'end |
| 2D21 | chr11:102253386-102253785 | 102253586 | 69 | 0.77 | 36 | Y | 11qE1 | BC025575 | 3'end |
| 2D25 | chr19:43493568-43493967 | 43493768 | 72 | 0.71 | 37 | Y | 19qC3 | Cnnm1 | 5'end |
| 2D27 | chr2:143961760-143962159 | 143961960 | 75 | 0.87 | 49 | Y | 2qG1 | Snx5/8430406I07Rik | 5'end |
| 2D29 | chr9:105253343-105253742 | 105253543 | 65 | 0.99 | 43 | Y | 9qF1 | Aste1 | 5'end |
| 2D30 | chr10:39881446-39881845 | 39881646 | 59 | 0.31 | 11 | N | 10qB1 | Intergenic | Intergenic |
| 2D39 | chr5:60519547-60519946 | 60519747 | 60 | 0.61 | 22 | Y | 5qC2 | CG869761 | 5'end |
| 2D41 | chr17:25866171-25866570 | 25866371 | 77 | 0.8 | 47 | Y | 17qA3.3 | Axin1 | 5'end |
| 2D43 | chr10:88873560-88873959 | 88873760 | 61 | 0.91 | 34 | Y | 10qC2 | Gas2l3 | 5'end |
| 2D47 | chr18:23453251-23453650 | 23453451 | 74 | 0.87 | 47 | Y | 18qA2 | AK147279 | 5'end |
| 2D48 | chr4:6380704-6381103 | 6380904 | 73 | 0.92 | 50 | Y | 4qA1 | Nsmaf | 5'end |
| 2E03 | chr4:145700274-145700673 | 145700474 | 76 | 0.77 | 45 | Y | 4qE1 | BC066875 | Body |
| 2E06 | chr13:91935454-91935853 | 91935654 | 78 | 1 | 60 | Y | 13qC3 | Ssbp2 | 5'end |
| 2E09 | chr1:155967178-155967577 | 155967378 | 65 | 0.76 | 31 | Y | 1qG3 | Intergenic | Intergenic |
| 2E10 | chr6:24905217-24905616 | 24905417 | 68 | 0.86 | 39 | Y | 6qA3.1 | 6332401O19Rik | 5'end |
| 2E14 | chr15:68192586-68192985 | 68192786 | 77 | 1.21 | 73 | Y | 15qD2 | BB645359 | 5'end |
| 2E25 | chr17:63184559-63184959 | 63184759 | 58 | 1.22 | 41 | Y | 17qE1.1 | AF176529 | 5'end |
| 2E30 | chr19:45713552-45713951 | 45713752 | 70 | 0.89 | 41 | Y | 19qC3 | Fbxw4 | 5'end |
| 2E34 | chr18:51242751-51243150 | 51242951 | 75 | 0.88 | 50 | Y | 18qD1 | AK046456 | 5'end |
| 2E37 | chr17:68741940-68742339 | 68742140 | 71 | 0.85 | 43 | Y | 17qE1.2 | AK041052 | 5'end |
| 2E38 | chr2:58961979-58962378 | 58962179 | 77 | 0.98 | 57 | Y | 2qC1.1 | Pkp4 | 5'end |
| 2E39 | chr17:33566371-33566770 | 33566571 | 58 | 0.71 | 24 | Y | 17qB1 | Rps18 | 5'end |
| 2E43 | chrX:148109835-148110234 | 148110035 | 69 | 0.84 | 39 | Y | XqF3 | DQ358971 | 5'end |
| 2E44 | chr16:46415663-46416062 | 46415863 | 74 | 1.02 | 57 | Y | 16qB5 | Pvrl3 | 5'end |
| 2E51 | chr13:63574097-63574496 | 63574297 | 60 | 0.65 | 23 | Y | 13qB3 | Ptch1 | 5'end |
| 2F08 | chr12:104932563-104932962 | 104932763 | 59 | 0.34 | 12 | N | 12qE | Intergenic | Intergenic |
| 2F12 | chr18:51242751-51243150 | 51242951 | 75 | 0.88 | 50 | Y | 18qD1 | AK046456 | 5'end |
| 2F16 | chr18:59300585-59300984 | 59300785 | 76 | 0.88 | 52 | Y | 18qD3 | AK044099 | 5'end |
| 2F19 | chr9:110377222-110377621 | 110377422 | 74 | 0.91 | 50 | Y | 9qF2 | AK045151 | 5'end |
| 2F21 | chr10:5633687-5634086 | 5633887 | 70 | 0.92 | 44 | Y | 10qA1 | Esr1 | Body |
| 2F28 | chr4:109174754-109175153 | 109174954 | 68 | 0.95 | 44 | Y | 4qC7 | Faf1 | 5'end |
| 2F31 | chr15:98924879-98925278 | 98925079 | 68 | 0.75 | 35 | Y | 15qF1 | 2810451A06Rik | 5'end |
| 2F36 | chr13:23429910-23430309 | 23430110 | 54 | 0.55 | 16 | N | 13qA3.1 | Abt1 | Body |
| 2F37 | chrX:57152859-57153258 | 57153059 | 76 | 1.01 | 57 | Y | XqA6 | Sox3 | 5'end |
| 2F39 | chr4:54965965-54966364 | 54966165 | 64 | 0.75 | 31 | Y | 4qB3 | AK044806 | 5'end |
| 2F53 | chr16:35076080-35076479 | 35076280 | 73 | 0.92 | 48 | Y | 16qB3 | Adcy5 | 5'end |
| 2F57 | chr14:75149540-75149939 | 75149740 | 79 | 0.86 | 54 | Y | 14qD3 | Tsc22d1 | 5'end |
| 2F61 | chr12:36552564-36552963 | 36552764 | 70 | 0.78 | 39 | Y | 12qA3 | Tspan13 | 5'end |
| 2F64 | chr14:62194702-62195101 | 62194902 | 74 | 0.72 | 40 | Y | 14qD1 | Gata4 | 5'end |
| 2F69 | chr12:72988848-72989247 | 72989048 | 68 | 0.89 | 41 | Y | 12qC3 | Gpr135 | 5'end |
| 2F72 | chr15:63827307-63827706 | 63827507 | 80 | 1.02 | 65 | Y | 15qD1 | 0910001A06Rik | Body |
| 2F73 | chr11:58992681-58993080 | 58992881 | 70 | 1 | 50 | Y | 11qB1.3 | Gja12 | 3'end |
| 2F79 | chr15:82181792-82182191 | 82181992 | 67 | 0.9 | 41 | Y | 15qE1 | Ndufa6 | 5'end |
| 2F89 | chr4:152325927-152326326 | 152326127 | 79 | 1.04 | 65 | Y | 4qE2 | Intergenic | Intergenic |
| 2F90 | chr4:11118207-11118606 | 11118407 | 72 | 0.94 | 49 | Y | 4qA1 | Ccne2 | 5'end |
| 2G05 | chr5:149680336-149680735 | 149680536 | 64 | 0.55 | 23 | N | 5qG3 | 6330406I15Rik | 5'end |
| 2G09 | chr10:9364758-9365157 | 9364958 | 66 | 0.71 | 31 | Y | 10qA1 | E130306M17Rik | 5'end |
| 2G14 | chr6:23789042-23789441 | 23789242 | 76 | 1.08 | 63 | Y | 6qA3.1 | Cadps2 | 5'end |
| 2G31 | chr7:143270419-143270818 | 143270619 | 74 | 0.96 | 53 | Y | 7NA | Cdkn1c | 5'end |
| 2G34 | chrX:22521793-22522192 | 22521993 | 69 | 0.54 | 25 | N | XqA2 | Klhl13 | 5'end |
| 2G35 | chr10:33313680-33314079 | 33313880 | 63 | 0.64 | 26 | Y | 10qA4 | A330019N05Rik | 5'end |
| 2G41 | chr13:48273380-48273779 | 48273580 | 70 | 1.03 | 51 | Y | 13qA5 | Id4 | 5'end |
| 2G48 | chr12:111893305-111893704 | 111893505 | 73 | 0.89 | 44 | Y | 12qF1 | Tnfaip2 | Body |
| 2G50 | chr12:68140755-68141154 | 68140955 | 71 | 0.87 | 43 | Y | 12qC2 | Mamdc1 | 5'end |
| 2G63 | chr5:136136750-136137150 | ######### | 72 | 0.87 | 26 | Y | 5qG2 | BC051947 | Body |
| 2G75 | chr2:19167456-19167855 | 19167656 | 49 | 0.51 | 12 | N | 2qA3 | AK041744 | Body |
| 2G81 | chr2:30426928-30427327 | 30427128 | 62 | 0.46 | 18 | N | 2qB | Cstad | Body |
| 2G93 | chr15:85562433-85562832 | 85562633 | 66 | 0.59 | 25 | N | 15qE2 | Ppara | 5'end |
| 2G95 | chr6:55132853-55133252 | 55133053 | 60 | 0.57 | 21 | N | 6qB3 | AK142385 | 5'end |
| 2G96 | chr14:13496425-13496824 | 13496625 | 76 | 1.05 | 61 | Y | 14qA1 | Slc4a7 | 5'end |
| 2H54 | chr11:66341793-66342192 | 66341993 | 76 | 1.13 | 65 | Y | 11qB3 | AK147504 | 5'end |
| 3A01 | chr10:128048519-128048918 | 128048719 | 65 | 0.58 | 25 | N | 10qD3 | Zfpn1a4 | 5'end |
| 3A03 | chr4:8617786-8618185 | 8617986 | 80 | 1.14 | 74 | Y | 4qA1 | Chd7 | 5'end |
| 3A04 | chr19:29317282-29317681 | 29317482 | 80 | 1.02 | 62 | Y | 19qC1 | Jak2 | 5'end |
| 3A10 | chr1:193838180-193838579 | 193838380 | 71 | 1.03 | 51 | Y | 1qH6 | Rcor3 | 5'end |
| 3B05 | chr9:88279595-88279994 | 88279795 | 73 | 0.99 | 52 | Y | 9qE3.1 | Syncrip | 5'end |
| 3B07 | chr2:20885857-20886256 | 20886057 | 73 | 0.9 | 48 | Y | 2qA3 | BC076629 | 5'end |
| 3B09 | chr4:128308720-128309119 | 128308920 | 75 | 0.81 | 45 | Y | 4qD2.2 | Intergenic | Intergenic |
| 3B13 | chr13:63574090-63574489 | 63574290 | 60 | 0.66 | 24 | Y | 13qB3 | Ptch1 | 5'end |
| 3B15 | chr13:31815321-31815720 | 31815521 | 74 | 0.97 | 53 | Y | 13qA3.2 | Foxc1 | 5'end |
| 3B16 | chr6:8728002-8728401 | 8728202 | 74 | 0.77 | 43 | Y | 6qA1 | Ica1 | 5'end |
| 3B24 | chr5:34137848-34138247 | 34138048 | 78 | 1.08 | 63 | Y | 5qB1 | AK038286 | 5'end |
| 3B25 | chr3:31285848-31286247 | 31286048 | 79 | 1.11 | 63 | Y | 3qA3 | Skil | 5'end |
| 3B27 | chr11:23156709-23157108 | 23156909 | 65 | 1.05 | 40 | Y | 11qA3.2 | Xpo1 | 5'end |
| 3B29 | chr11:107731178-107731577 | 107731378 | 54 | 0.3 | 9 | N | 11qE1 | Cacng5 | 5'end |
| 3B30 | chr10:25767617-25768016 | 25767817 | 68 | 0.93 | 43 | Y | 10qA4 | C030003D03Rik | 5'end |
| 3B35 | chr5:108607069-108607468 | 108607269 | 68 | 0.85 | 40 | Y | 5qF | AK165889 | 5'end |
| 3B38 | chr15:85498919-85499318 | 85499119 | 74 | 1.08 | 59 | Y | 15qE2 | BE988697 | 5'end |
| 3B45 | chr16:18002389-18002788 | 18002589 | 68 | 0.86 | 40 | Y | 16qA3 | Prodh | 5'end |
| 3B46 | chr2:68271588-68271987 | 68271788 | 63 | 0.77 | 31 | Y | 2qC2 | Stk39 | 5'end |
| 3C01 | chr5:101904820-101905219 | 101905020 | 70 | 1.1 | 55 | Y | 5qE5 | Nkx6-1 | 5'end |
| 3C02 | chr18:5593115-5593514 | 5593315 | 63 | 0.77 | 31 | Y | 18qA1 | Zfhx1a | 5'end |
| 3C03 | chr11:98610857-98611256 | 98611057 | 72 | 0.91 | 45 | Y | 11qD | 4121402D02Rik | 5'end |
| 3C06 | chr1:15271805-15272204 | 15272005 | 76 | 0.9 | 53 | Y | 1qA3 | Intergenic | Intergenic |
| 3C09 | chr9:87527664-87528063 | 87527864 | 69 | 0.65 | 30 | Y | 9qE3.1 | Tbx18 | 5'end |
| 3C10 | chr2:84515789-84516188 | 84515989 | 76 | 1.22 | 68 | Y | 2qD | Zdhhc5 | 5'end |
| 3C13 | chr2:105804009-105804408 | 105804209 | 53 | 0.82 | 23 | Y | 2qE3 | Zcsl3 | 5'end |
| 3C16 | chr6:49248997-49249396 | 49249197 | 61 | 0.7 | 26 | Y | 6qB2.3 | 6330407D12Rik | 5'end |
| 3C20 | chr15:68758058-68758457 | 68758258 | 80 | 1.07 | 69 | Y | 15qD3 | Khdrbs3 | 5'end |
| 3C21 | chr19:6428416-6428815 | 6428616 | 70 | 1.02 | 51 | Y | 19qA | Nrxn2 | 5'end |
| 3C24 | chr9:87528799-87529198 | 87528999 | 56 | 0.85 | 26 | Y | 9qE3.1 | Tbx18 | 5'end |
| 3C27 | chr2:74456451-74456850 | 74456651 | 77 | 0.99 | 59 | Y | 2qC3 | Evx2 | 5'end |
| 3C38 | chr17:24743200-24743599 | 24743400 | 70 | 1.09 | 54 | Y | 17qA3.3 | Ift140 | 5'end |
| 3C39 | chrX:9874085-9874484 | 9874285 | 67 | 0.62 | 28 | Y | XqA1.1 | Mid1ip1 | 5'end |
| 3C41 | chr4:47373082-47373481 | 47373282 | 45 | 0.7 | 14 | N | 4qB1 | Tgfbr1 | 5'end |
| 3D03 | chr2:63898991-63899390 | 63899191 | 82 | 0.98 | 66 | Y | 2qC1.3 | Fign | 5'end |
| 3D04 | chr8:109945584-109945983 | 109945784 | 64 | 1.03 | 43 | Y | 8qD3 | Cog8 | 5'end |
| 3D07 | chr3:138679484-138679883 | 138679684 | 81 | 1.12 | 73 | Y | 3qH1 | Tspan5 | 5'end |
| 3D10 | chr5:43523429-43523828 | 43523629 | 58 | 0.68 | 23 | Y | 5qB3 | Cpeb2 | 5'end |
| 3D19 | chr5:135678371-135678770 | 135678571 | 61 | 0.93 | 32 | Y | 5qG2 | Pom121 | 5'end |
| 3D22 | chr4:88745566-88745965 | 88745766 | 43 | 0.54 | 10 | N | 4qC4 | Cdkn2a | 3'end |
| 3D27 | chr4:133432906-133433305 | 133433106 | 57 | 0.61 | 19 | N | 4qD3 | Ccdc21 | Body |
| 3D28 | chr12:74465011-74465410 | 74465211 | 75 | 0.84 | 48 | Y | 12qC3 | Tmem30b | 5'end |
| 3D29 | chr10:117247863-117248262 | 117248063 | 49 | 0.57 | 14 | N | 10qD2 | Rap1b | Body |
| 3D30 | chr11:63739893-63740292 | 63740093 | 51 | 0.61 | 16 | N | 11qB3 | Hs3st3b1 | 5'end |
| 3D31 | chr3:10070577-10070976 | 10070777 | 55 | 1.26 | 39 | Y | 3qA1 | BC060946 | 5'end |
| 3D36 | chr16:32982109-32982508 | 32982309 | 76 | 1.04 | 60 | Y | 16qB3 | Lmln | 5'end |
| 3D38 | chr1:158078410-158078809 | 158078610 | 47 | 0.58 | 13 | N | 1qG3 | Intergenic | Intergenic |
| 3D44 | chr18:34496992-34497391 | 34497192 | 49 | 0.41 | 10 | N | 18qB1 | Dp1 | Body |
| 3D46 | chr11:29273814-29274213 | 29274014 | 72 | 0.81 | 42 | Y | 11qA3.3 | A430106J12Rik | 5'end |
| 3D47 | chr1:39422796-39423195 | 39422996 | 71 | 0.96 | 49 | Y | 1qB | Tbc1d8 | 5'end |
| 3D49 | chr6:38228592-38228991 | 38228792 | 75 | 0.84 | 47 | Y | 6qB1 | B130055L09Rik | 5'end |
| 3D55 | chr17:55959803-55960202 | 55960003 | 71 | 0.99 | 49 | Y | 17qC | Jmjd2b | 5'end |
| 3D59 | chr5:143518564-143518963 | 143518764 | 72 | 1.17 | 61 | Y | 5qG2 | Zfp316 | 3'end |
| 3D62 | chr14:69370823-69371222 | 69371023 | 69 | 0.58 | 26 | N | 14qD1 | AK158744 | 5'end |
| 3D63 | chr12:102870010-102870409 | 102870210 | 75 | 0.94 | 53 | Y | 12qE | Golga5 | 5'end |
| 3D67 | chr4:131385508-131385907 | 131385708 | 66 | 0.81 | 35 | Y | 4qD2.3 | Oprd1 | 3'end |
| 3E03 | chr2:118064626-118065025 | 118064826 | 72 | 0.67 | 35 | Y | 2qE5 | Gpr176 | 5'end |
| 3E07 | chr11:22872237-22872636 | 22872437 | 58 | 0.86 | 29 | Y | 11qA3.2 | U2af1-rs1 | 5'end |
| 3E09 | chr6:58836507-58836906 | 58836707 | 60 | 0.69 | 25 | Y | 6qB3 | Nap1l5 | 5'end |
| 3E11 | chr9:51920748-51921147 | 51920948 | 78 | 1 | 59 | Y | 9qA5.3 | BC042784 | 3'end |
| 3E13 | chr19:7449715-7450114 | 7449915 | 72 | 1.01 | 51 | Y | 19qA | AK163590 | 5'end |
| 3E30 | chr12:104872338-104872737 | 104872538 | 61 | 0.84 | 31 | Y | 12qE | Gsc | 3'end |
| 3E33 | chr19:45713552-45713951 | 45713752 | 70 | 0.89 | 41 | Y | 19qC3 | Fbxw4 | 5'end |
| 3E34 | chr6:99658668-99659067 | 99658868 | 71 | 0.88 | 45 | Y | 6qD3 | Gpr27 | 5'end |
| 3E44 | chr10:59920325-59920724 | 59920525 | 53 | 0.28 | 8 | N | 10qB4 | Cdh23 | Body |
| 3E52 | chr10:127814519-127814918 | 127814719 | 65 | 0.69 | 28 | Y | 10qD3 | Rnf41 | 5'end |
| 3E54 | chr10:66931259-66931658 | 66931459 | 63 | 0.84 | 34 | Y | 10qB5.1 | Egr2 | 5'end |
| 3E55 | chr1:37485347-37485746 | 37485547 | 78 | 0.96 | 56 | Y | 1qB | Mgat4a | 5'end |
| 3E59 | chr1:22087203-22087602 | 22087403 | 36 | 0.09 | 1 | N | 1qA5 | Intergenic | Intergenic |
| 3E60 | chr16:18534327-18534726 | 18534527 | 72 | 0.99 | 51 | Y | 16qA3 | Gp1bb | 5'end |
| 3E61 | chr8:81035641-81036040 | 81035841 | 47 | 0.81 | 18 | N | 8qC1 | Intergenic | Intergenic |
| 3E74 | chr7:133614756-133615155 | 133614956 | 71 | 0.85 | 42 | Y | 7qF3 | Dhx32/Fank1 | 5'end |
| 3E75 | chr14:28549455-28549854 | 28549655 | 69 | 0.91 | 43 | Y | 14qA3 | Cacna2d3 | 5'end |
| 3E77 | chr10:94118467-94118866 | 94118667 | 73 | 0.85 | 46 | Y | 10qC2 | 4921537D05Rik | 5'end |
| 3F01 | chr11:114496517-114496916 | 114496717 | 55 | 0.23 | 7 | N | 11qE2 | Ttyh2 | Body |
| 3F03 | chr11:118871946-118872345 | 118872146 | 57 | 0.73 | 24 | Y | 11qE2 | Intergenic | Intergenic |
| 3F06 | chr1:88985243-88985642 | 88985443 | 70 | 1.08 | 52 | Y | 1qC5 | Ecel1 | 5'end |
| 3F09 | chr3:45477569-45477968 | 45477769 | 58 | 0.71 | 24 | Y | 3qB | Pcdh10 | 5'end |
| 3F14 | chr2:140087095-140087494 | 140087295 | 76 | 0.92 | 53 | Y | 2qF3 | 2900006F19Rik | 5'end |
| 3F18 | chr19:4000554-4000953 | 4000754 | 66 | 0.88 | 39 | Y | 19qA | Nudt8 | 5'end |
| 3F19 | chr5:106117052-106117451 | 106117252 | 73 | 0.89 | 48 | Y | 5qE5 | Zfp326 | 5'end |
| 3F21 | chr3:57935393-57935792 | 57935593 | 80 | 1.03 | 65 | Y | 3qD | Pfn2 | 5'end |
| 3F27 | chr10:80855923-80856322 | 80856123 | 56 | 0.62 | 19 | N | 10qC1 | Intergenic | Intergenic |
| 3F33 | chr16:96232211-96232609 | 96232411 | 72 | 1.04 | 53 | Y | 16qC4 | Hmgn1 | 5'end |
| 3F41 | chr3:57935416-57935815 | 57935616 | 80 | 1.06 | 66 | Y | 3qD | Pfn2 | 5'end |
| 3F42 | chr1:4482348-4482747 | 4482548 | 69 | 0.84 | 40 | Y | 1qA1 | Sox17 | 3'end |
| 3F45 | chr2:30426921-30427320 | 30427121 | 63 | 0.46 | 18 | N | 2qB | Cstad | Body |
| 3F55 | chr11:79809502-79809901 | 79809702 | 74 | 0.99 | 51 | Y | 11qB5 | Suz12 | 5'end |
| 3F56 | chr12:50259599-50259998 | 50259799 | 72 | 0.92 | 48 | Y | 12qB3 | Foxg1 | 5'end |
| 3F60 | chr6:116303338-116303737 | 116303538 | 63 | 1.01 | 40 | Y | 6qE3 | March8 | 5'end |
| 3F61 | chr12:23491868-23492267 | 23492068 | 72 | 0.98 | 52 | Y | 12qA1.3 | CJ089436 | 5'end |
| 3F63 | chr12:74827367-74827766 | 74827567 | 63 | 0.93 | 37 | Y | 12qC3 | Hif1a | 5'end |
| 3F70 | chr14:115809252-115809651 | 115809452 | 68 | 0.71 | 33 | Y | 14qE4 | Gpc6 | 5'end |
| 3F72 | chr2:86299604-86300003 | 86299804 | 51 | 0.38 | 10 | N | 2qD | Intergenic | Intergenic |
| 3F76 | chr2:74456251-74456650 | 74456451 | 75 | 0.94 | 52 | Y | 2qC3 | Evx2 | 3'end |
| 3F84 | chr12:45139365-45139764 | 45139565 | 73 | 0.96 | 50 | Y | 12qB1 | 1200006O19Rik | 5'end |
| 3G10 | chr18:74804859-74805258 | 74805059 | 53 | 0.19 | 5 | N | 18qE3 | Myo5b | Body |
| 3G102 | chr2:144859250-144859649 | 144859450 | 77 | 1.06 | 62 | Y | 2qG1 | AF314821 | 5'end |
| 3G118 | chr17:35857032-35857431 | 35857232 | 63 | 0.62 | 25 | Y | 17qB1 | AK138323 | 5'end |
| 3G119 | chr4:77683102-77683501 | 77683302 | 75 | 0.88 | 50 | Y | 4qC3 | AK034145 | 5'end |
| 3G30 | chr9:58622031-58622430 | 58622231 | 69 | 0.88 | 42 | Y | 9qB | AF064874 | 5'end |
| 3G35 | chr5:74476549-74476948 | 74476749 | 71 | 0.79 | 40 | Y | 5qC3.3 | BC083068 | 5'end |
| 3G37 | chr4:123189237-123189636 | 123189437 | 78 | 0.93 | 57 | Y | 4qD2.2 | DQ067088 | 5'end |
| 3G60 | chr3:62426508-62426907 | 62426708 | 66 | 0.76 | 34 | Y | 3qE1 | AK087060 | 5'end |
| 3G72 | chr2:102201596-102201995 | 102201796 | 76 | 1.06 | 60 | Y | 2qE2 | Trim44 | 5'end |
| 3G77 | chr11:95821838-95822237 | 95822038 | 64 | 0.86 | 36 | Y | 11qD | Igf2bp1 | 5'end |
| 3G87 | chr17:36555250-36555649 | 36555450 | 68 | 0.83 | 39 | Y | 17qB1 | BC051578 | 3'end |
| 3G89 | chr17:68741933-68742332 | 68742133 | 71 | 0.82 | 42 | Y | 17qE1.2 | AK041052 | 5'end |
| 3G90 | chr2:173753333-173753732 | 173753533 | 78 | 0.71 | 44 | Y | 2qH4 | Npepl1 | 5'end |
| 3G91 | chr7:143270419-143270818 | 143270619 | 74 | 0.96 | 53 | Y | 7NA | Cdkn1c | 5'end |
| 3G92 | chr7:109494970-109495369 | 109495170 | 65 | 0.67 | 27 | Y | 7qE3 | ICRFP703B1614Q5.6 | 5'end |
| 3G99 | chrX:105037023-105037422 | 105037223 | 60 | 0.5 | 17 | N | XqD | AK050539 | 5'end |
| 3H06 | chr11:45823678-45824077 | 45823878 | 80 | 0.98 | 62 | Y | 11qB1.1 | Sox30 | 5'end |
| 3H13 | chr8:12397011-12397410 | 12397211 | 75 | 1.09 | 61 | Y | 8qA1.1 | Sox1 | 5'end |
| 3H19 | chr13:23746275-23746674 | 23746475 | 65 | 0.73 | 31 | Y | 13qA3.1 | Hist1h1c | 5'end |
| 4A12 | chr10:78957527-78957926 | 78957727 | 70 | 0.94 | 45 | Y | 10qC1 | 2700087H15Rik | 5'end |
| 4A18 | chr11:102253794-102254193 | 102253994 | 70 | 1.05 | 51 | Y | 11qE1 | Grn | 3'end |
| 4A23 | chr17:87015978-87016377 | 87016178 | 76 | 1.02 | 59 | Y | 17qE4 | Socs5 | 5'end |
| 4B03 | chr12:109240106-109240504 | 109240306 | 69 | 1.26 | 60 | Y | 12qF1 | Yy1 | 5'end |
| 4B05 | chr5:124801099-124801498 | 124801299 | 68 | 1.02 | 48 | Y | 5qG1.1 | Tmed2 | 5'end |
| 4B07 | chr3:144507356-144507755 | 144507556 | 74 | 1 | 51 | Y | 3qH2 | Hs2st1/Sep15 | 5'end |
| 4B09 | chr3:121418521-121418920 | 121418721 | 70 | 1.01 | 50 | Y | 3qG1 | Cnn3 | 5'end |
| 4B11 | chr2:139369458-139369857 | 139369658 | 74 | 0.74 | 41 | Y | 2qF3 | AK090153 | 5'end |
| 4B12 | chr15:96114419-96114818 | 96114619 | 64 | 0.97 | 40 | Y | 15qF1 | AK007269 | 5'end |
| 4B16 | chr2:68272435-68272834 | 68272635 | 76 | 0.91 | 53 | Y | 2qC2 | Stk39 | 5'end |
| 4B18 | chr2:92285827-92286226 | 92286027 | 48 | 0.76 | 17 | N | 2qE1 | AK036400 | body |
| 4B19 | chr11:61224349-61224748 | 61224549 | 69 | 0.76 | 36 | Y | 11qB2 | AK086839 | 5'end |
| 4B20 | chr11:61224349-61224748 | 61224549 | 69 | 0.76 | 36 | Y | 11qB2 | AK086839 | 5'end |
| 4B21 | chr7:34923479-34923878 | 34923679 | 81 | 0.88 | 58 | Y | 7qB1 | AK149337 | 5'end |
| 4B22 | chr5:131822050-131822449 | 131822250 | 67 | 0.57 | 26 | N | 5qG2 | BC066072 | 5'end |
| 4B27 | chr13:99454698-99455097 | 99454898 | 76 | 1.01 | 59 | Y | 13qD1 | Foxd1 | 5'end |
| 4B28 | chr16:94677914-94678314 | 94678114 | 53 | 1.35 | 195 | Y | 16qC4 | Dyrk1a | 5'end |
| 4B29 | chr10:79387247-79387646 | 79387447 | 71 | 1.09 | 55 | Y | 10qC1 | ORF61 | 5'end |
| 4B38 | chr12:109240106-109240504 | 109240306 | 69 | 1.26 | 60 | Y | 12qF1 | Yy1 | 5'end |
| 4B41 | chr11:43280592-43280991 | 43280792 | 47 | 0.51 | 11 | N | 11qB1.1 | D11Ertd730e | 5'end |
| 4B42 | chr3:62426516-62426915 | 62426716 | 66 | 0.77 | 34 | Y | 3qE1 | AK087060 | 5'end |
| 4C01 | chr11:115675186-115675585 | 115675386 | 65 | 0.62 | 26 | Y | 11qE2 | AK044818 | 5'end |
| 4C10 | chr13:31815328-31815727 | 31815528 | 74 | 0.97 | 53 | Y | 13qA3.2 | Foxc1 | 5'end |
| 4C11 | chr16:10552747-10553146 | 10552947 | 53 | 0.39 | 11 | N | 16qA1 | 4932416N17Rik | Body |
| 4C12 | chr10:70743084-70743483 | 70743284 | 76 | 0.92 | 54 | Y | 10qB5.3 | Ipmk | 5'end |
| 4C13 | chr7:79382100-79382499 | 79382300 | 70 | 0.97 | 48 | Y | 7qD2 | AK039621 | 5'end |
| 4C14 | chr14:62194710-62195109 | 62194910 | 75 | 0.69 | 39 | Y | 14qD1 | Gata4 | Body |
| 4C18 | chrX:69926449-69926848 | 69926649 | 67 | 0.8 | 36 | Y | XqA7.3 | Slc6a8 | 5'end |
| 4C19 | chr9:61168783-61169182 | 61168983 | 61 | 1.02 | 38 | Y | 9qB | Tle3 | 5'end |
| 4C22 | chr16:91294960-91295359 | 91295160 | 71 | 0.66 | 32 | Y | 16qC4 | Il10rb | 5'end |
| 4C26 | chr5:129334273-129334672 | 129334473 | 68 | 0.97 | 45 | Y | 5qG1.3 | Ran | 5'end |
| 4C31 | chr16:35151792-35152191 | 35151992 | 50 | 0.28 | 7 | N | 16qB3 | Adcy5 | Body |
| 4D01 | chr4:130889810-130890209 | 130890010 | 51 | 0.11 | 3 | N | 4qD2.3 | BC040767 | Body |
| 4D02 | chr11:84580689-84581088 | 84580889 | 64 | 0.43 | 18 | N | 11qC | Intergenic | Intergenic |
| 4D09 | chr4:145945780-145946179 | 145945980 | 78 | 1.14 | 70 | Y | 4qE1 | BC066875 | Body |
| 4D11 | chr14:104782709-104783109 | 104782909 | 50 | 1.14 | 29 | Y | 14qE2.3 | Spry2 | 5'end |
| 4D15 | chr15:82181785-82182184 | 82181985 | 68 | 0.93 | 43 | Y | 15qE1 | Ndufa6 | 5'end |
| 4D17 | chrX:104018442-104018841 | 104018642 | 60 | 0.49 | 17 | N | XqD | 2610002M06Rik | 5'end |
| 4D26 | chr10:18007718-18008117 | 18007918 | 78 | 0.85 | 52 | Y | 10qA3 | Intergenic | Intergenic |
| 4D27 | chr6:52093816-52094215 | 52094016 | 63 | 0.89 | 35 | Y | 6qB3 | Hoxa2 | 5'end |
| 4D28 | chr10:126714323-126714722 | 126714523 | 62 | 0.89 | 35 | Y | 10qD3 | Mars | 5'end |
| 4D29 | chr8:74309407-74309806 | 74309607 | 65 | 0.69 | 29 | Y | 8qB3.3 | Nr2f6 | 5'end |
| 4D36 | chr18:36413425-36413824 | 36413625 | 66 | 0.83 | 36 | Y | 18qB2 | Pura | 5'end |
| 4D44 | chr5:112348267-112348666 | 112348467 | 52 | 0.26 | 7 | N | 5qF | AK139722 | 5'end |
| 4D45 | chr2:71329684-71330083 | 71329884 | 69 | 0.58 | 27 | N | 2qC2 | Dlx1 | 5'end |
| 4D49 | chr13:25277309-25277708 | 25277509 | 67 | 0.85 | 37 | Y | 13qA3.1 | Vmp | 5'end |
| 4D54 | chr10:107736937-107737336 | 107737137 | 77 | 1.03 | 62 | Y | 10qD1 | Pawr | 5'end |
| 4D57 | chr4:28983956-28984355 | 28984156 | 72 | 0.79 | 41 | Y | 4qA4 | Epha7 | 5'end |
| 4D58 | chr5:28401958-28402357 | 28402158 | 72 | 1.01 | 53 | Y | 5qB1 | Insig1 | 5'end |
| 4D65 | chr5:121921077-121921476 | 121921277 | 74 | 1.03 | 57 | Y | 5qF | Brap | 5'end |
| 4D69 | chr10:82193259-82193658 | 82193459 | 82 | 1 | 68 | Y | 10qC1 | Nfyb | 5'end |
| 4E01 | chr9:105252493-105252892 | 105252693 | 45 | 0.24 | 5 | N | 9qF1 | Aste1 | 5'end |
| 4E02 | chr8:122796598-122796997 | 122796798 | 73 | 0.98 | 53 | Y | 8qE1 | Usp10 | 5'end |
| 4E04 | chr4:43544490-43544889 | 43544690 | 65 | 0.62 | 83 | Y | 4qB1 | Tpm2 | 5'end |
| 4E05 | chr13:63574097-63574496 | 63574297 | 60 | 0.65 | 23 | Y | 13qB3 | Ptch1 | 5'end |
| 4E15 | chr5:106693621-106694020 | 106693821 | 67 | 0.67 | 30 | Y | 5qE5 | E130309B19Rik | 3'end |
| 4E18 | chr4:115460820-115461219 | 115461020 | 68 | 1.11 | 51 | Y | 4qD1 | Intergenic | Intergenic |
| 4E21 | chr4:126470007-126470406 | 126470207 | 82 | 0.89 | 59 | Y | 4qD2.2 | AK129137 | 5'end |
| 4E24 | chr11:70030560-70030959 | 70030760 | 72 | 0.68 | 35 | Y | 11qB3 | Slc16a11 | 5'end |
| 4E26 | chr9:98765713-98766112 | 98765913 | 76 | 0.97 | 54 | Y | 9qE3.3 | Foxl2 | 5'end |
| 4E27 | chr9:68452837-68453236 | 68453037 | 67 | 0.94 | 42 | Y | 9qC | Rora | 5'end |
| 4E29 | chr6:52105294-52105693 | 52105494 | 77 | 0.89 | 52 | Y | 6qB3 | 5730596B20Rik | 5'end |
| 4E34 | chr5:104699920-104700319 | 104700120 | 72 | 1 | 52 | Y | 5qE5 | Pkd2 | 5'end |
| 4E40 | chr3:32900078-32900477 | 32900278 | 63 | 0.96 | 39 | Y | 3qA3 | Actl6a | 5'end |
| 4E41 | chr15:79260277-79260676 | 79260477 | 53 | 0.63 | 18 | N | 15qE1 | Csnk1e | Body |
| 4E44 | chr18:89028277-89028676 | 89028477 | 76 | 0.77 | 44 | Y | 18qE4 | Socs6 | 5'end |
| 4E47 | chr6:91082022-91082421 | 91082222 | 75 | 0.97 | 55 | Y | 6qD1 | Nup210 | 5'end |
| 4E58 | chr8:95744404-95744803 | 95744604 | 68 | 0.88 | 41 | Y | 8qC5 | Aytl1 | 5'end |
| 4E70 | chr11:74988494-74988893 | 74988694 | 67 | 0.98 | 44 | Y | 11qB5 | Hic1 | 5'end |
| 4E72 | chr11:11586102-11586501 | 11586302 | 66 | 0.91 | 40 | Y | 11qA1 | Zfpn1a1 | 5'end |
| 4E73 | chr2:155433626-155434025 | 155433826 | 52 | 0.4 | 11 | N | 2qH1 | Intergenic | Intergenic |
| 4F06 | chr14:29182577-29182976 | 29182777 | 80 | 0.95 | 60 | Y | 14qA3 | Cacna1d | 5'end |
| 4F09 | chr1:91772267-91772666 | 91772467 | 51 | 0.45 | 12 | N | 1qD | Intergenic | Intergenic |
| 4F21 | chr10:80962508-80962907 | 80962708 | 77 | 0.94 | 56 | Y | 10qC1 | Aes | 5'end |
| 4F28 | chr11:118869070-118869469 | 118869270 | 56 | 0.81 | 26 | Y | 11qE2 | Intergenic | Intergenic |
| 4F43 | chr5:106695789-106696188 | 106695989 | 53 | 0.35 | 10 | N | 5qE5 | E130309B19Rik | Body |
| 4F44 | chr11:53194404-53194803 | 53194604 | 66 | 1.06 | 47 | Y | 11qB1.3 | Aff4 | 5'end |
| 4F46 | chr11:53194404-53194803 | 53194604 | 66 | 1.06 | 47 | Y | 11qB1.3 | Aff4 | 5'end |
| 4F52 | chr11:90454413-90454812 | 90454613 | 71 | 0.95 | 48 | Y | 11qD | Stxbp4/Cox11 | 5'end |
| 4F55 | chr4:134705457-134705856 | 134705657 | 68 | 0.75 | 35 | Y | 4qD3 | AK155270 | 5'end |
| 4F59 | chr16:21118547-21118946 | 21118747 | 69 | 0.67 | 32 | Y | 16qB1 | Ephb3 | 5'end |
| 4F83 | chr4:46159538-46159937 | 46159738 | 71 | 1.11 | 56 | Y | 4qB1 | BC057893 | 5'end |
| 4F84 | chr17:34585383-34585782 | 34585583 | 57 | 0.87 | 29 | Y | 17qB1 | Lsm2 | 5'end |
| 4F94 | chr15:98924579-98924978 | 98924779 | 64 | 0.77 | 32 | Y | 15qF1 | 2810451A06Rik | 5'end |
| 4G04 | chr2:76207191-76207590 | 76207391 | 77 | 0.97 | 57 | Y | 2qC3 | Osbpl6 | 5'end |
| 4G102 | chr10:43266789-43267188 | 43266989 | 67 | 0.81 | 36 | Y | 10qB2 | Cd24a | 5'end |
| 4G103 | chr12:88867733-88868132 | 88867933 | 59 | 0.82 | 28 | Y | 12_randomNA | Cables2 | 5'end |
| 4G106 | chr1:16604060-16604459 | 16604260 | 73 | 1.03 | 54 | Y | 1qA3 | AK050923 | 5'end |
| 4G11 | chr19:24097705-24098104 | 24097905 | 83 | 0.96 | 66 | Y | 19qB | Gm967 | 5'end |
| 4G27 | chr11:97838503-97838902 | 97838703 | 74 | 0.94 | 50 | Y | 11qD | Cacnb1 | 5'end |
| 4G43 | chr10:39881501-39881900 | 39881701 | 56 | 0.28 | 9 | N | 10qB1 | Intergenic | Intergenic |
| 4G54 | chr12:5400922-5401321 | 5401122 | 69 | 0.66 | 31 | Y | 12qA1.1 | BC065168 | 5'end |
| 4G63 | chr1:19096139-19096538 | 19096339 | 45 | 0.86 | 18 | N | 1qA3 | Tcfap2d | Body |
| 4G67 | chr16:59542945-59543344 | 59543145 | 78 | 0.96 | 58 | Y | 16qC1.3 | BF462921 | 5'end |
| 4G73 | chr8:94688652-94689051 | 94688852 | 68 | 0.77 | 36 | Y | 8qC5 | D230002A01Rik | 5'end |
| 4G84 | chr2:19575249-19575648 | 19575449 | 73 | 0.98 | 52 | Y | 2qA3 | AK076525 | 5'end |
| 4G96 | chr2:109718751-109719150 | 109718951 | 76 | 1.01 | 59 | Y | 2qE3 | CJ093786 | 5'end |
| 4G97 | chr18:46324044-46324443 | 46324244 | 72 | 0.88 | 45 | Y | 18qC | Trim36 | Body |
| 5A03 | chr7:34826607-34827006 | 34826807 | 62 | 0.85 | 33 | Y | 7qB1 | Cebpa | 5'end |
| 5A05 | chr7:34827142-34827541 | 34827342 | 62 | 0.77 | 30 | Y | 7qB1 | Cebpa | 5'end |
| 5A13 | chr17:87015978-87016377 | 87016178 | 76 | 1.02 | 59 | Y | 17qE4 | Socs5 | 5'end |
| 5A19 | chr13:58817284-58817683 | 58817484 | 66 | 0.81 | 35 | Y | 13qB2 | Ntrk2 | 5'end |
| 5B12 | chr11:23155934-23156333 | 23156134 | 67 | 0.78 | 34 | Y | 11qA3.2 | Xpo1 | 5'end |
| 5B13 | chr4:99143138-99143537 | 99143338 | 57 | 0.9 | 30 | Y | 4qC6 | Foxd3 | 5'end |
| 5B15 | chr4:11118214-11118613 | 11118414 | 72 | 0.93 | 49 | Y | 4qA1 | Ccne2 | 5'end |
| 5B17 | chr11:3232117-3232516 | 3232317 | 68 | 0.91 | 42 | Y | 11qA1 | 1500004A08Rik | body |
| 5B21 | chr13:106414318-106414717 | 106414518 | 77 | 0.86 | 51 | Y | 13qD2.1 | AK151379 | 5'end |
| 5B27 | chr10:28835928-28836327 | 28836128 | 57 | 0.64 | 21 | Y | 10qA4 | 6330407J23Rik | 5'end |
| 5B28 | chr13:99454698-99455097 | 99454898 | 76 | 1.01 | 59 | Y | 13qD1 | Foxd1 | 5'end |
| 5B30 | chr7:100805075-100805474 | 100805275 | 67 | 0.84 | 38 | Y | 7qE2 | ARHGEF17 | 5'end |
| 5B31 | chrX:105037023-105037422 | 105037223 | 60 | 0.5 | 17 | N | XqD | AK050539 | 5'end |
| 5B33 | chr7:63817181-63817580 | 63817381 | 76 | 1.05 | 60 | Y | 7qC | Klf13 | 5'end |
| 5B38 | chr3:144507356-144507755 | 144507556 | 74 | 1 | 51 | Y | 3qH2 | Hs2st1 | 5'end |
| 5B40 | chr3:31285090-31285489 | 31285290 | 83 | 0.81 | 55 | Y | 3qA3 | Skil | 5'end |
| 5B44 | chr11:118857480-118857879 | 118857680 | 65 | 0.72 | 29 | Y | 11qE2 | Cbx8 | 5'end |
| 5B45 | chr15:96114419-96114818 | 96114619 | 64 | 0.97 | 40 | Y | 15qF1 | AK007269 | 5'end |
| 5B51 | chr15:68758051-68758450 | 68758251 | 81 | 1.06 | 69 | Y | 15qD3 | Khdrbs3 | 5'end |
| 5C01 | chr11:98610857-98611256 | 98611057 | 72 | 0.91 | 45 | Y | 11qD | 4121402D02Rik | 5'end |
| 5C04 | chr16:17710712-17711111 | 17710912 | 79 | 0.87 | 55 | Y | 16qA3 | Scarf2 | 5'end |
| 5C10 | chr17:27350648-27351047 | 27350848 | 75 | 1.03 | 59 | Y | 17qA3.3 | Nudt3 | 5'end |
| 5C16 | chr10:62120954-62121353 | 62121154 | 71 | 1 | 51 | Y | 10qB4 | AK028920 | 5'end |
| 5C17 | chr12:55780623-55781022 | 55780823 | 75 | 0.98 | 56 | Y | 12qC1 | Cfl2 | 5'end |
| 5C19 | chr5:101904820-101905219 | 101905020 | 70 | 1.1 | 55 | Y | 5qE5 | Nkx6-1 | 5'end |
| 5C23 | chr5:48271189-48271588 | 48271389 | 69 | 0.96 | 43 | Y | 5qB3 | Slit2 | 5'end |
| 5C25 | chr16:84718020-84718419 | 84718220 | 77 | 1.08 | 62 | Y | 16qC3.3 | Atp5j/Gabpa | 5'end |
| 5C29 | chr15:98921468-98921867 | 98921668 | 66 | 0.81 | 33 | Y | 15qF1 | 2810451A06Rik | Body |
| 5C30 | chr13:60185347-60185746 | 60185547 | 70 | 0.95 | 47 | Y | 13qB3 | Gas1 | 5'end |
| 5C32 | chr10:122389322-122389721 | 122389522 | 69 | 1.11 | 53 | Y | 10qD2 | AK052706 | 5'end |
| 5C43 | chr11:115066553-115066952 | 115066753 | 58 | 0.73 | 25 | Y | 11qE2 | Grin2c | Body |
| 5D07 | chr4:41872193-41872592 | 41872393 | 81 | 0.92 | 57 | Y | 4qA5 | Cntfr | Body |
| 5D11 | chr17:31917613-31918012 | 31917813 | 59 | 0.69 | 24 | Y | 17qA3.3 | Abhd9 | 5'end |
| 5D15 | chr11:63741419-63741818 | 63741619 | 66 | 0.66 | 29 | Y | 11qB3 | Hs3st3b1 | 5'end |
| 5D23 | chr4:108278527-108278926 | 108278727 | 78 | 1 | 61 | Y | 4qC7 | AK011275 | 5'end |
| 5D25 | chr7:36405144-36405543 | 36405344 | 72 | 0.75 | 39 | Y | 7qB2 | Zfp537 | 5'end |
| 5D36 | chr4:48072835-48073234 | 48073035 | 70 | 0.67 | 32 | Y | 4qB1 | Nr4a3 | 5'end |
| 5D37 | chr2:68272743-68273142 | 68272943 | 78 | 1.01 | 61 | Y | 2qC2 | Stk39 | 5'end |
| 5D38 | chr7:144706443-144706842 | 144706643 | 56 | 0.9 | 29 | Y | 7NA | Fgf15 | 5'end |
| 5D40 | chr13:48273373-48273772 | 48273573 | 71 | 1.02 | 52 | Y | 13qA5 | Id4 | 5'end |
| 5D42 | chr5:52854386-52854785 | 52854586 | 77 | 0.86 | 52 | Y | 5qC1 | Lgi2 | 5'end |
| 5D43 | chr2:76207198-76207597 | 76207398 | 77 | 0.99 | 59 | Y | 2qC3 | Osbpl6 | 5'end |
| 5D45 | chr3:41660156-41660555 | 41660356 | 56 | 1.27 | 39 | Y | 3qB | AK147466 | 5'end |
| 5D50 | chr4:11249044-11249443 | 11249244 | 73 | 0.82 | 42 | Y | 4qA1 | AK144417 | 5'end |
| 5D51 | chr4:59720416-59720815 | 59720616 | 76 | 0.89 | 51 | Y | 4qB3 | E130308A19Rik | 5'end |
| 5D52 | chr5:72860069-72860468 | 72860269 | 62 | 0.64 | 25 | Y | 5qC3.2 | Zar1 | 5'end |
| 5E01 | chr11:118869070-118869469 | 118869270 | 56 | 0.81 | 26 | Y | 11qE2 | Intergenic | Intergenic |
| 5E02 | chr15:91400313-91400712 | 91400513 | 77 | 1.07 | 63 | Y | 15qE3 | A630029G22Rik | 5'end |
| 5E04 | chr6:140386444-140386843 | 140386644 | 76 | 0.99 | 57 | Y | 6qG2 | CJ170110 | 5'end |
| 5E05 | chr19:11885692-11886091 | 11885892 | 77 | 0.93 | 55 | Y | 19qA | Stx3 | 5'end |
| 5E16 | chr19:56874888-56875287 | 56875088 | 67 | 1.08 | 48 | Y | 19qD2 | Tdrd1 | 5'end |
| 5E17 | chr19:10456278-10456677 | 10456478 | 82 | 0.88 | 60 | Y | 19qA | Syt7 | 5'end |
| 5E24 | chr10:28836690-28837089 | 28836890 | 67 | 0.65 | 30 | Y | 10qA4 | AK044134 | 5'end |
| 5E26 | chr5:130193389-130193788 | 130193589 | 63 | 0.81 | 32 | Y | 5qG1.3 | 4930579G22Rik | 5'end |
| 5E31 | chr6:55132853-55133252 | 55133053 | 60 | 0.57 | 21 | N | 6qB3 | C330043M08Rik | 5'end |
| 5E42 | chr2:91764299-91764698 | 91764499 | 78 | 0.9 | 54 | Y | 2qE1 | Dgkz | 5'end |
| 5E47 | chr10:79897786-79898185 | 79897986 | 68 | 0.67 | 31 | Y | 10qC1 | Onecut3 | 5'end |
| 5E52 | chr8:72811437-72811836 | 72811637 | 69 | 1.04 | 50 | Y | 8qB3.3 | AK004006 | 5'end |
| 5E57 | chr17:83123151-83123550 | 83123351 | 79 | 0.77 | 48 | Y | 17qE4 | AW548124 | 5'end |
| 5E61 | chr11:119758961-119759360 | 119759161 | 67 | 0.9 | 41 | Y | 11qE2 | Baiap2 | 5'end |
| 5E65 | chr1:57914379-57914778 | 57914579 | 71 | 0.93 | 47 | Y | 1qC1.3 | 6530404F10Rik | 5'end |
| 5F09 | chr8:87504602-87505001 | 87504802 | 47 | 0.37 | 8 | N | 8qC2 | Cacna1a | Body |
| 5F30 | chr15:76775588-76775987 | 76775788 | 60 | 0.86 | 31 | Y | 15qD3 | 1110038F14Rik | 5'end |
| 5F34 | chr12:84810767-84811166 | 84810967 | 63 | 0.6 | 22 | Y | 12qD1 | BC033459 | 5'end |
| 5F49 | chr2:25913732-25914131 | 25913932 | 65 | 0.56 | 24 | N | 2qA3 | Btbd14a | 5'end |
| 5F52 | chr1:133966852-133967251 | 133967052 | 69 | 0.73 | 35 | Y | 1qE4 | Pctk3 | 5'end |
| 5F57 | chr15:76537605-76538004 | 76537805 | 68 | 0.93 | 44 | Y | 15qD3 | Recql4/Lrrc14 | 5'end |
| 5F58 | chr4:59720409-59720808 | 59720609 | 76 | 0.89 | 52 | Y | 4qB3 | E130308A19Rik | 5'end |
| 5F59 | chr9:47281964-47282363 | 47282164 | 75 | 0.91 | 51 | Y | 9qA5.3 | Igsf4a | 5'end |
| 5F63 | chr11:118853582-118853981 | 118853782 | 58 | 0.79 | 27 | Y | 11qE2 | Cbx8 | 3'end |
| 5F66 | chr11:88004174-88004573 | 88004374 | 53 | 0.61 | 17 | N | 11qC | Cuedc1 | Body |
| 5F70 | chr18:47493914-47494313 | 47494114 | 65 | 0.82 | 35 | Y | 18qC | Sema6a | 5'end |
| 5F75 | chr14:69625594-69625993 | 69625794 | 53 | 0.69 | 20 | Y | 14qD1 | Gfra2 | 5'end |
| 5F77 | chr9:96173120-96173519 | 96173320 | 72 | 0.95 | 50 | Y | 9qE3.3 | Atp1b3 | 5'end |
| 5F78 | chr2:130255096-130255495 | 130255296 | 71 | 0.95 | 48 | Y | 2qF1 | Mrps26 | 5'end |
| 5G109 | chr11:97842874-97843273 | 97843074 | 61 | 1 | 36 | Y | 11qD | Rpl19 | 5'end |
| 5G11 | chr13:23717981-23718380 | 23718181 | 56 | 0.44 | 14 | N | 13qA3.1 | Hfe | 5'end |
| 5G16 | chr3:84345456-84345855 | 84345656 | 60 | 0.88 | 32 | Y | 3qF1 | AK147275 | 5'end |
| 5G21 | chr7:65249574-65249973 | 65249774 | 78 | 0.94 | 57 | Y | 7qC | Tjp1 | 5'end |
| 5G22 | chr11:96280581-96280980 | 96280781 | 67 | 0.75 | 34 | Y | 11qD | Scap1 | 5'end |
| 5G39 | chr2:74459760-74460159 | 74459960 | 64 | 0.87 | 35 | Y | 2qC3 | Evx2 | 5'end |
| 5G43 | chr3:104916769-104917168 | 104916969 | 73 | 0.6 | 32 | Y | 3qF2.2 | Rhoc | 5'end |
| 5G45 | chr11:106428803-106429202 | 106429003 | 78 | 0.86 | 52 | Y | 11qE1 | Tex2 | 5'end |
| 5G51 | chr11:106428803-106429202 | 106429003 | 78 | 0.86 | 52 | Y | 11qE1 | Tex2 | 5'end |
| 5G61 | chr9:45181066-45181465 | 45181266 | 69 | 0.65 | 31 | Y | 9qA5.2 | AF487346 | 5'end |
| 5G63 | chr12:98975898-98976297 | 98976098 | 71 | 0.86 | 44 | Y | 12qE | 1700024D23Rik | 5'end |
| 5G67 | chr16:35991088-35991487 | 35991288 | 74 | 0.93 | 51 | Y | 16qB3 | AI413631 | 5'end |
| 5G68 | chr8:24440063-24440462 | 24440263 | 83 | 1.03 | 71 | Y | 8qA2 | AK158131 | 5'end |
| 5G90 | chr12:81051477-81051876 | 81051677 | 70 | 0.89 | 44 | Y | 12qD1 | AK160272 | 5'end |
| 5H01 | chr11:120489066-120489465 | 120489266 | 76 | 0.94 | 53 | Y | 11qE2 | Aspscr1 | 5'end |
| 5H105 | chr11:98753989-98754388 | 98754189 | 63 | 0.73 | 29 | Y | 11qD | Rara | 5'end |
| 5H33 | chr4:120147337-120147736 | 120147537 | 73 | 0.82 | 43 | Y | 4qD2.2 | M10062 | Body |
| 5H45 | chr11:115675186-115675585 | 115675386 | 65 | 0.62 | 26 | Y | 11qE2 | AK044818 | 5'end |
| 6A04 | chr11:102253794-102254193 | 102253994 | 70 | 1.05 | 51 | Y | 11qE1 | Grn | 3'end |
| 6A19 | chr10:78957527-78957926 | 78957727 | 70 | 0.94 | 45 | Y | 10qC1 | 2700087H15Rik | 5'end |
| 6B09 | chr3:76042183-76042582 | 76042383 | 66 | 0.95 | 41 | Y | 3qE3 | Golph4 | 5'end |
| 6B15 | chr18:47493921-47494320 | 47494121 | 65 | 0.81 | 35 | Y | 18qC | Sema6a | 5'end |
| 6B16 | chr11:118856606-118857005 | 118856806 | 68 | 0.65 | 30 | Y | 11qE2 | Cbx8 | 5'end |
| 6B24 | chr5:131822050-131822449 | 131822250 | 67 | 0.57 | 26 | N | 5qG2 | BC066072 | 5'end |
| 6B29 | chr9:106744809-106745208 | 106745009 | 80 | 1.17 | 76 | Y | 9qF1 | Rbm15b | 5'end |
| 6B31 | chr7:63817181-63817580 | 63817381 | 76 | 1.05 | 60 | Y | 7qC | Klf13 | 5'end |
| 6B37 | chr5:108607650-108608049 | 108607850 | 78 | 0.94 | 58 | Y | 5qF | AK165889 | 5'end |
| 6B38 | chr16:11047491-11047890 | 11047691 | 60 | 0.69 | 25 | Y | 16qA1 | Txndc11 | 5'end |
| 6C06 | chr19:9056415-9056814 | 9056615 | 60 | 0.93 | 33 | Y | 19qA | Ahnak | 5'end |
| 6C17 | chr4:99149817-99150216 | 99150017 | 70 | 0.74 | 36 | Y | 4qC6 | Foxd3 | 5'end |
| 6C19 | chr18:5592211-5592610 | 5592411 | 80 | 1.17 | 74 | Y | 18qA1 | Zfhx1a | 5'end |
| 6C21 | chr9:85123570-85123969 | 85123770 | 73 | 0.97 | 51 | Y | 9qE3.1 | AK135132 | 5'end |
| 6C22 | chr13_random:32110-32509 | 32310 | 70 | 0.84 | 41 | Y | 13_randomNA | Zfp131 | 5'end |
| 6C23 | chr16:96186281-96186680 | 96186481 | 68 | 0.7 | 33 | Y | 16qC4 | Brwd1 | 5'end |
| 6C24 | chr7:79383207-79383606 | 79383407 | 54 | 0.34 | 10 | N | 7qD2 | AK039621 | 5'end |
| 6C25 | chr10:27764309-27764708 | 27764509 | 64 | 0.8 | 33 | Y | 10qA4 | Ptprk | 5'end |
| 6C26 | chr7:131334125-131334524 | 131334325 | 73 | 0.94 | 47 | Y | 7qF3 | Hmx3 | 5'end |
| 6C29 | chr6:124930864-124931263 | 124931064 | 71 | 1 | 50 | Y | 6qF2 | Cops7a | 5'end |
| 6C31 | chr1:64671811-64672210 | 64672011 | 80 | 0.94 | 57 | Y | 1qC2 | Fzd5 | 5'end |
| 6C33 | chr1:64670600-64670999 | 64670800 | 64 | 0.71 | 29 | Y | 1qC2 | Fzd5 | 5'end |
| 6C34 | chr4:140810442-140810841 | 140810642 | 80 | 1.06 | 69 | Y | 4qE1 | AK137647 | 5'end |
| 6D01 | chr17:26443116-26443515 | 26443316 | 73 | 1.07 | 57 | Y | 17qA3.3 | A930001N09Rik | 5'end |
| 6D04 | chr5:32411688-32412087 | 32411888 | 66 | 1.05 | 46 | Y | 5qB1 | Fosl2 | 5'end |
| 6D06 | chr16:8653286-8653688 | 8653486 | 55 | 1.3 | 40 | Y | 16qA1 | AK135814 | Body |
| 6D07 | chr10:90512392-90512791 | 90512592 | 72 | 1.02 | 52 | Y | 10qC2 | Apaf1 | 5'end |
| 6D08 | chr19:4756289-4756688 | 4756489 | 70 | 0.92 | 46 | Y | 19qA | 4921506I22Rik | 5'end |
| 6D10 | chr19:45729217-45729616 | 45729417 | 48 | 0.82 | 19 | N | 19qC3 | Intergenic | Intergenic |
| 6D12 | chr2:135350773-135351172 | 135350973 | 79 | 1.02 | 63 | Y | 2qF3 | AK036896 | 5'end |
| 6D16 | chrX:52721516-52721915 | 52721716 | 64 | 0.79 | 32 | Y | XqA5 | 6330419J24Rik | 5'end |
| 6D20 | chr4:141511227-141511626 | 141511427 | 74 | 0.98 | 52 | Y | 4qE1 | AK052809 | 5'end |
| 6D24 | chr6:86815079-86815478 | 86815279 | 70 | 0.98 | 48 | Y | 6qD1 | Aak1 | 5'end |
| 6D30 | chr7:122761267-122761666 | 122761467 | 76 | 0.87 | 49 | Y | 7qF3 | Rbbp6 | 5'end |
| 6D39 | chr4:28982620-28983019 | 28982820 | 72 | 0.99 | 51 | Y | 4qA4 | Epha7 | 5'end |
| 6D46 | chrX:6871016-6871415 | 6871216 | 66 | 0.9 | 40 | Y | XqA1.1 | BY268211 | 5'end |
| 6E02 | chr6:86815079-86815478 | 86815279 | 70 | 0.98 | 48 | Y | 6qD1 | Aak1 | 5'end |
| 6E05 | chr2:25084481-25084880 | 25084681 | 66 | 0.91 | 39 | Y | 2qA3 | C430004E15Rik | 5'end |
| 6E07 | chr6:61109818-61110217 | 61110018 | 78 | 1.05 | 65 | Y | 6qB3 | BC107398 | 5'end |
| 6E14 | chr8:129950137-129950536 | 129950337 | 74 | 1 | 56 | Y | 8qE2 | Pard3 | 5'end |
| 6E22 | chr14:60602286-60602685 | 60602486 | 76 | 1.01 | 57 | Y | 14qC3 | AK033134 | 5'end |
| 6E36 | chr18:38223806-38224205 | 38224006 | 55 | 0.42 | 13 | N | 18qB3 | Intergenic | Intergenic |
| 6E38 | chr4:145945780-145946179 | 145945980 | 78 | 1.14 | 70 | Y | 4qE1 | BC066875 | Body |
| 6E40 | chr11:98361693-98362092 | 98361893 | 68 | 1.14 | 53 | Y | 11qD | AK036657 | 5'end |
| 6E41 | chr10:74410801-74411200 | 74411001 | 80 | 0.95 | 61 | Y | 10qB5.3 | Gnaz | 5'end |
| 6E43 | chr6:87816251-87816650 | 87816451 | 71 | 0.95 | 48 | Y | 6qD1 | Cnbp1 | 5'end |
| 6E44 | chr5:106693622-106694021 | 106693822 | 67 | 0.67 | 30 | Y | 5qE5 | E130309B19Rik | 3'end |
| 6F08 | chr15:76537605-76538004 | 76537805 | 68 | 0.93 | 44 | Y | 15qD3 | Recql4 | 5'end |
| 6F27 | chr12:70905421-70905820 | 70905621 | 79 | 1.13 | 71 | Y | 12qC2 | Sav1 | 5'end |
| 6F31 | chr11:114605218-114605617 | 114605418 | 63 | 0.63 | 25 | Y | 11qE2 | BF451207 | 3'end |
| 6F58 | chr16:17122152-17122551 | 17122352 | 69 | 0.86 | 41 | Y | 16qA3 | Gm603 | 5'end |
| 6F59 | chr9:40481648-40482047 | 40481848 | 57 | 0.49 | 16 | N | 9qA5.1 | 9030425E11Rik | Body |
| 6G04 | chr6:61109818-61110217 | 61110018 | 78 | 1.05 | 65 | Y | 6qB3 | BC107398 | 5'end |
| 6G05 | chr10:79119562-79119961 | 79119762 | 77 | 0.85 | 50 | Y | 10qC1 | Hcn2 | 5'end |
| 6G20 | chr14:29258594-29258993 | 29258794 | 54 | 0.27 | 8 | N | 14qA3 | Cacna1d | Body |
| 6G23 | chr19:45284562-45284961 | 45284762 | 65 | 1.02 | 43 | Y | 19qC3 | Lbx1 | 3'end |
| 6G24 | chr19:45288161-45288560 | 45288361 | 68 | 0.87 | 41 | Y | 19qC3 | Lbx1 | 5'end |
| 6G25 | chr16:20646855-20647254 | 20647055 | 67 | 0.72 | 31 | Y | 16qA3 | Chrd | 5'end |
| 6G28 | chr11:113883099-113883498 | 113883299 | 71 | 0.82 | 42 | Y | 11qE2 | Sdk2 | 5'end |
| 6G33 | chr6:146127577-146127976 | 146127777 | 47 | 0.54 | 12 | N | 6qG3 | Itpr2 | Body |
| 6G41 | chr10:80158365-80158764 | 80158565 | 76 | 0.99 | 57 | Y | 10qC1 | Dot1l | 5'end |
| 6G46 | chr14:61509649-61510048 | 61509849 | 74 | 1.04 | 57 | Y | 14qD1 | Intergenic | Intergenic |
| 6G67 | chr7:64380668-64381067 | 64380868 | 73 | 1.05 | 56 | Y | 7qC | Apba2 | 5'end |
| 6G69 | chr15:98916279-98916678 | 98916479 | 67 | 0.68 | 30 | Y | 15qF1 | C1ql4 | 5'end |
| 6H15 | chr2:145594484-145594883 | 145594684 | 63 | 1.03 | 41 | Y | 2qG1 | Nat5 | 5'end |
| 7B01 | chr2:139370023-139370422 | 139370223 | 67 | 0.95 | 43 | Y | 2qF3 | AK090153 | 5'end |
| 7B04 | chr19:4000554-4000953 | 4000754 | 66 | 0.88 | 39 | Y | 19qA | Nudt8 | 5'end |
| 7B15 | chr10:41963831-41964230 | 41964031 | 72 | 0.91 | 48 | Y | 10qB2 | Foxo3a | 5'end |
| 7D10 | chr1:191622947-191623346 | 191623147 | 78 | 0.91 | 55 | Y | 1qH6 | Smyd2 | 5'end |
| 7E09 | chr4:101048601-101049000 | 101048801 | 62 | 0.83 | 32 | Y | 4qC6 | Dnajc6 | 5'end |
| 7F24 | chr19:16200052-16200451 | 16200252 | 76 | 1.07 | 62 | Y | 19qA | Gnaq | 5'end |
| 7G10 | chr12:86967413-86967812 | 86967613 | 50 | 0.5 | 12 | N | 12qD2 | Tgfb3 | 5'end |
